# A comprehensive AI‐driven analysis of large‐scale omic datasets reveals novel dual‐purpose targets for the treatment of cancer and aging

**DOI:** 10.1111/acel.14017

**Published:** 2023-10-27

**Authors:** Frank W. Pun, Geoffrey Ho Duen Leung, Hoi Wing Leung, Jared Rice, Tomas Schmauck‐Medina, Sofie Lautrup, Xi Long, Bonnie Hei Man Liu, Chun Wai Wong, Ivan V. Ozerov, Alex Aliper, Feng Ren, Ari J. Rosenberg, Nishant Agrawal, Evgeny Izumchenko, Evandro F. Fang, Alex Zhavoronkov

**Affiliations:** ^1^ Insilico Medicine Hong Kong Ltd. Hong Kong China; ^2^ Department of Clinical Molecular Biology University of Oslo and Akershus University Hospital Lørenskog Norway; ^3^ Insilico Medicine AI Ltd. Masdar City United Arab Emirates; ^4^ Insilico Medicine Shanghai Ltd. Shanghai China; ^5^ Department of Medicine, Section of Hematology and Oncology University of Chicago Chicago Illinois USA; ^6^ Department of Surgery University of Chicago Chicago Illinois USA; ^7^ The Norwegian Centre On Healthy Ageing (NO‐Age) Oslo Norway; ^8^ Buck Institute for Research on Aging Novato California USA

**Keywords:** anti‐aging, artificial intelligence, cancer, hallmarks of aging, longevity, multiomics, target discovery

## Abstract

As aging and tumorigenesis are tightly interconnected biological processes, targeting their common underlying driving pathways may induce dual‐purpose anti‐aging and anti‐cancer effects. Our transcriptomic analyses of 16,740 healthy samples demonstrated tissue‐specific age‐associated gene expression, with most tumor suppressor genes downregulated during aging. Furthermore, a large‐scale pan‐cancer analysis of 11 solid tumor types (11,303 cases and 4431 control samples) revealed that many cellular processes, such as protein localization, DNA replication, DNA repair, cell cycle, and RNA metabolism, were upregulated in cancer but downregulated in healthy aging tissues, whereas pathways regulating cellular senescence were upregulated in both aging and cancer. Common cancer targets were identified by the AI‐driven target discovery platform—PandaOmics. Age‐associated cancer targets were selected and further classified into four groups based on their reported roles in lifespan. Among the 51 identified age‐associated cancer targets with anti‐aging experimental evidence, 22 were proposed as dual‐purpose targets for anti‐aging and anti‐cancer treatment with the same therapeutic direction. Among age‐associated cancer targets without known lifespan‐regulating activity, 23 genes were selected based on predicted dual‐purpose properties. Knockdown of histone demethylase KDM1A, one of these unexplored candidates, significantly extended lifespan in *Caenorhabditis elegans*. Given KDM1A's anti‐cancer activities reported in both preclinical and clinical studies, our findings propose KDM1A as a promising dual‐purpose target. This is the first study utilizing an innovative AI‐driven approach to identify dual‐purpose target candidates for anti‐aging and anti‐cancer treatment, supporting the value of AI‐assisted target identification for drug discovery.

## Graphical abstract

This study identified common dysregulated genes and pathways in aging and cancer, revealing tissue‐specific age‐associated gene expression and cellular processes. Using an AI‐driven approach, we identified multiple novel dual‐purpose targets for cancer and aging treatment. Notably, we highlighted KDM1A as a promising dual‐purpose target, with our findings demonstrating lifespan extension in *C. elegans* and opening new avenues for therapeutic advancements.

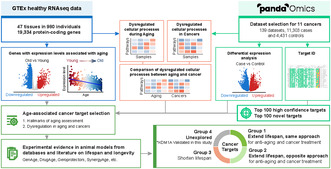

**Abbreviations**
AI: Artificial Intelligence
*C. elegans*
: *Caenorhabditis elegans*
CHG: Cancer Hallmark Genes
COSMIC: Catalogue of Somatic Mutations in Cancer
ECM: Extracellular matrix
FDA: U.S. Food and Drug Administration
FDR: False discovery rate
GO: Gene Ontology
GTEx: Genotype‐Tissue Expression
iPANDA: In silico Pathway Activation Network Decomposition Analysis
KDM1A: Lysine‐specific demethylase 1A
LogFC: Log‐fold change
NCG: Network of Cancer Genes
RNAi: RNA interference
SASP: Senescence‐associated secretory phenotype
SEER: Surveillance Epidemiology and End Results
TSG: Tumor suppressor gene

## 1 INTRODUCTION

Aging is an irreversible process with progressive functional decline, which can lead to multiple chronic age‐related diseases including cancer. According to the World Health Organization, the proportion of the world's population over 60 years old will reach 22% by 2050. Cancer incidence increases dramatically with age, showing sharp elevation after 55 years old and reaching the peak at around 80 based on the National Cancer Institute's Surveillance, Epidemiology, and End Results (SEER) program. Recent studies have gained important insights into the interplay of aging and carcinogenesis by studying proteomic and epigenetic changes underlying a complex interrelationship between both processes (Daniel & Tollefsbol, 2015; Gong et al., 2021). Several aging hallmarks (Lopez‐Otin et al., 2013; Lopez‐Otin, Blasco, et al., 2023), including chronic inflammation, dysregulated nutrient sensing, dysbiosis, epigenetic alterations, and genomic instability, are overlapping with hallmarks of cancer and may contribute to the development of malignant disease (Lopez‐Otin, Pietrocola, et al., 2023). While gene expression analyses have also been used to study the link between aging and cancer (Chatsirisupachai et al., 2019; Saul & Kosinsky, 2021), the biological mechanisms underlying the age‐related increased risk of cancer remain poorly understood.

Gerotherapeutic drugs aim to reduce the burden of age‐related diseases and increase healthspan and lifespan. For example, the mTOR inhibitor rapamycin is currently among the most promising anti‐aging drugs. It can extend the lifespan by up to 60% and improves the healthspan in vivo (Bitto et al., 2016). In addition, rapamycin and other mTOR inhibitors such as everolimus and temsirolimus have also been approved for the treatment of cancer by the U.S. Food and Drug Administration (FDA) (Ballou & Lin, 2008; Populo et al., 2012), implying that targeting shared signaling pathways or mechanisms underlying both processes may suggest a promising dual‐purpose therapeutic intervention for anti‐aging and anti‐cancer.

The hallmarks of aging (Lopez‐Otin et al., 2013; Lopez‐Otin, Blasco, et al., 2023) have been widely used as a starting point to study geriatric pathologies. The hallmarks that represent common characteristics of aging include altered intercellular communication, cellular senescence, deregulated nutrient signaling, epigenetic shift, genomic instability, impaired proteostasis, mitochondrial dysfunction, stem cell exhaustion, and telomere attrition (Lopez‐Otin et al., 2013; Pun, Leung, et al., 2022), as well as compromised autophagy, microbiome disturbance, altered mechanical properties, splicing dysregulation, processes associated with extracellular matrix (ECM) stiffness, inflammation, and retrotransposition (Pun, Leung, et al., 2022; Schmauck‐Medina et al., 2022). Notably, a subset of these hallmarks such as genomic instability, ECM stiffness, inflammation, cellular senescence, and deregulated nutrient signaling are also attributed to tumorigenesis (Aunan et al., 2017; Blagosklonny, 2022; Collado et al., 2007; Yun & Lee, 2018), suggesting their intersected function in aging and proliferative processes.

Genomic instability, attributable to unrepaired DNA damage, is progressively increasing during aging and carcinogenesis (Aunan et al., 2017). ECM damage is suggested to be driven through various modifications to the ECM constituents, including fragmentation, glycation, cross‐linking, and accumulation of protein aggregation, leading to ECM stiffness (Selman & Pardo, 2021). Abnormal ECM remodeling also creates a microenvironment that promotes cellular transformation, tumorigenesis, and metastasis (Winkler et al., 2020). Furthermore, chronic aging‐related inflammation (inflammaging) is frequently observed in older adults due to the dysregulated immune system (Fulop et al., 2023). Chronic inflammatory microenvironment promotes cancer cell proliferation, survival, and migration by triggering the release of pro‐inflammatory cytokines and chemokines (Greten & Grivennikov, 2019). In addition, cellular senescence can also be triggered in aging and cancer by various mechanisms such as DNA damage, tumor suppressor loss‐of‐function, and epigenomic damage (Collado et al., 2007). As aging and cancer share multiple interconnected biological processes, targeting the mechanisms underlying these common hallmarks may potentially delay aging in healthy individuals and cure cancer in patients.

Recently, our group developed and used PandaOmics, an AI‐driven target discovery engine (Pun et al., 2023), to identify a list of dual‐purpose target candidates for delaying the onset of age‐related pathologies, including neurological, metabolic, fibrotic, and inflammatory diseases (Pun, Leung, et al., 2022). As this comprehensive platform was also proven capable of discovering novel therapeutic targets and biomarkers for cancer and amyotrophic lateral sclerosis (Mkrtchyan et al., 2022; Pun, Liu, et al., 2022), here we aimed to identify potential dual‐purpose targets that could delay aging and increase lifespan among healthy individuals as well as inhibit cancer using a list of common cancer targets derived from PandaOmics. By analyzing the age‐associated genes and common cancer targets prioritized by AI, we predicted a list of potential dual‐purpose candidates and validated an unreported dual‐purpose candidate, KDM1A (lysine‐specific demethylase 1A), in C. *elegans*.

## 2 METHODS

### 2.1 Disease and dataset selection

Access to Genotype‐Tissue Expression (GTEx) V9‐protected data (phs000424.v9.p2) was authorized by the Data Access Committee of NCBI dbGAP. Raw tissue‐specific RNA‐sequencing (available at https://gtexportal.org/home/) and sample attribute data were downloaded. All samples were considered as non‐diseased by GTEx. Only the tissues with 100 or more samples were selected for analysis in this study. Cancers were included to this study based on the following criteria: (1) The peak incidence is at 50 years old or older based on literature and NCI SEER; (2) it is a primary solid tumor; and (3) the tumor site matches at least 1 GTEx healthy tissue with 100 or more sequenced samples. Consequently, 11 solid cancers and 11 site‐matched healthy tissues were selected for studying the relationships between aging and cancer. In cases (i.e., colon and esophagus) where multiple regions of an organ were available, we selected the regions that are more prone to develop tumors. An additional collection of 36 GTEx healthy tissues was analyzed to study the relationships between gene expression and aging. As a result, a total of 47 healthy tissues consisting of 16,740 samples were included (Table S1). For the selected cancers, datasets were obtained from different public repositories. A total of 131 transcriptomics datasets, including microarray and RNA‐sequencing, were retrieved from TCGA, GEO and ArrayExpress, while eight proteomics datasets were retrieved from Proteomic Data Commons (PDC) and Proteomics Identification Database (PRIDE). Datasets were selected for each cancer based on the following criteria: (1) The dataset contains at least two cancer samples and two control samples; (2) no prior treatment was given to the patients before obtaining the sample; and (3) only human samples were included. As a result, 139 cancer datasets consisting of 11,303 cases and 4431 controls were included (Table S1). As detailed metadata was not available for all individuals across multiple datasets used in this study, confounders such as smoking, alcohol consumption, and geographical location were not considered for the analysis. Raw microarray and RNA‐sequencing data were normalized by quantile and upper‐quartile normalization, respectively, and filtered for protein‐coding genes using R package *biomaRt*. Genes with zero coverage in ≥25% of samples were removed. Variance‐stabilizing normalization was used to normalize the proteomics datasets. Proteins with zero abundance in ≥25% of samples were removed. A total of 252 protein‐coding TSGs were retrieved from the Network of Cancer Genes Home (NCG) v7.0 (available at http://ncg.kcl.ac.uk/, Table S3). Hallmark(s) of cancer for each AI‐derived cancer target was/were retrieved from the Catalogue of Somatic Mutations in Cancer (COSMIC) and Cancer Hallmark Genes (CHG) databases (available at https://cancer.sanger.ac.uk/cosmic and http://bio‐bigdata.hrbmu.edu.cn/CHG/, respectively).

### 2.2 Identification of age‐associated genes

For each tissue, age‐associated genes were identified by the combination of two analyses using the GTEx dataset. Two‐sided partial Pearson's correlation analysis was first performed to calculate the correlation coefficient between age and expression level for each gene while controlling for the effects exerted by sex:

$$r_{\mathrm{ae},s}=\frac{r_{\mathrm{ae}}-r_{\mathrm{as}}∙r_{\mathrm{es}}}{\sqrt{\left(1-r_{\mathrm{as}}^{2}\right)∙\left(1-r_{\mathrm{es}}^{2}\right)}}$$

where *r*_*ae*_ is the correlation between age and expression level, *r*_*as*_ is the correlation between age and sex, and *r*_*es*_ is the correlation between expression level and sex. *p*‐values for partial correlation were calculated based on the t‐distribution with *n*‐2 degrees of freedom. Linear regression analysis using R package *Limma* was also performed to identify differentially expressed genes between the old (age ≥ 60) and young (age < 50) individuals:

$$Y_{\mathrm{ij}}=\beta_{1}{\mathrm{Age}}_{i}+\beta_{2}{\mathrm{Sex}}_{i}+\varepsilon_{\mathrm{ij}}$$

where *Y*_*ij*_ is the expression level of gene *j* in sample *i*, *Age*_*i*_ is the age of sample *i*, *Sex*_*i*_ is the sex of sample *i*, and *ε*_*ij*_ is the error term. *p*‐values for differential expression analysis were calculated by moderated *t*‐test implemented in *Limma*. Only the genes with statistically significant results (*p* < 0.05) from both analyses were considered as age‐associated in a tissue.

### 2.3 Identification of dysregulated cellular processes in cancers and aging

To study the dysregulation of cellular processes during aging, iPANDA was performed using data from each of the 139 cancer datasets and 11 corresponding GTEx tissues to identify activated or inhibited pathways between the cases and the controls (i.e., cancer vs noncancerous samples for cancers; old vs young samples for aging). iPANDA calculates the activation or inhibition score for each pathway by combining precalculated gene coexpression data with gene importance factors based on the degree of differential gene expression and pathway topology decomposition. Dysregulated pathways with iPANDA score >0.01 or <−0.01 were considered as activated and inhibited, respectively, and extracted. For pathways that were dysregulated in multiple datasets in each cancer, only those that were dysregulated in ≥50% of datasets in a unidirectional manner were selected. Arithmetic mean was then applied to the extracted iPANDA scores and was further selected if the mean iPANDA score was >0.01 or <−0.01. These pathways were then mapped to a total of 27 main cellular processes based on the hierarchical structure of pathways in the Reactome database. The percentage of each main cellular process that was activated or inhibited was calculated based on the total number of pathways involved in that cellular process.

### 2.4 Meta‐analysis

For each cancer dataset, case and control samples from the same tissue source were selected and compared, resulting in a total of 141 comparisons (Table S1). All of the performed comparisons were pooled into a single meta‐analysis for each cancer, yielding a total of 11 meta‐analyses subjected to target identification.

### 2.5 AI models and filter settings used for target identification

Targets were prioritized by PandaOmics (available at https://pandaomics.com/) using its AI hypothesis generation models based on 23 scores from Omics, Text‐based, Financial, and KOL scores. The background of PandaOmics was previously described^24^. The loss of novelty would be a trade‐off for the abundance of evidence connecting a target to a disease. Regarding this, a list of targets in (1) high confidence setting and (2) novel setting based on the volume of related publications proposed by PandaOmics' proprietary AI engine was identified, respectively. To identify high confidence targets for each cancer, 8 omics scores, including Network neighbors, Causal inference, Pathways, Interactome community, Relevance, Expression, Heterogeneous graph walk, and Matrix factorization, were applied, along with 2 Text‐based scores (Evidence and Attention score), 1 Financial score (Grant funding), and 2 KOL scores (Credible attention index and Impact factor). Additionally, only the targets that belong to druggable classes, accessible by small molecules, and were not considered as essential genes in the Online GEne Essentiality database were selected. The description of each scoring model and filter used was included in Table S9. The genes were then ranked based on the selected scores and filters, and the top 100 ranked genes obtained for each cancer were extracted as high confidence druggable targets. Similarly, novel targets were identified using the same omics scores and druggability filter settings, with the novelty level being set to a higher degree while all Text‐based, Financial, and KOL scores were disabled. The top 100 ranked genes for each cancer were then selected as novel targets for further analysis.

### 2.6 Identification of common cancer targets and expression analysis

For each of the two novelty settings, a list of the top 100 ranked genes in each of the 11 cancers was extracted, generating a combined list of 301 high confidence and 319 novel druggable targets. The genes were then prioritized by their (1) descending occurrence and (2) ascending average ranking across multiple cancers, and those top 100 genes were selected as the common cancer targets. As a result, a total of 200 common cancer targets were identified. For each of the 200 common cancer targets, its LogFC values in each case–control comparison were extracted. Since there were multiple datasets for each cancer, a combined LogFC value was calculated for each gene by multiplying with standard deviation while *p*‐values were combined by Stouffer's method. False discovery rate (FDR) was calculated to correct for multiple comparisons.

### 2.7 Hallmarks of aging assessment

To assess the hallmark(s) of aging for each gene, a list of keywords that could be found on the Gene Ontology Resource (available at http://geneontology.org/) was constructed and linked to each hallmark based on Lopez‐Otin et al. (2013) and Pun, Leung, et al. (2022). Gene Ontology (GO) terms, including biological process, cellular component, and molecular function for each of the 200 genes, were retrieved using R package *biomaRt* and mapped to the corresponding hallmarks. The list of genes associated with the hallmark(s) of aging was then manually examined.

### 2.8 Age‐associated cancer target selection

The two hundred top 100 high confidence and novel common cancer targets were first filtered based on their expression changes during aging and association with the hallmark(s) of aging. Targets were selected if they were considered as age‐associated in more than 10% of the 47 healthy tissues and associated with at least one hallmark of aging. After filtering, the targets were subjected to literature search for experimental evidence demonstrating their roles in regulating lifespan in animal models including *Caenorhabditis elegan*s, *Drosophila melanogaster,* and *Mus musculus* (Table S6). Publicly available databases were used to retrieve the evidence including GenAge, DrugAge, Geroprotectors, and SynergyAge. For compounds obtained from Geroprotectors and DrugAge that were shown to affect lifespan, their target genes were retrieved from ChEMBL, a manually curated database of bioactive molecules with drug‐like properties, and mapped to the targets to infer their associations with lifespan regulation. Drugs or geroprotectors targeting specific genes (i.e., ≤2 genes) only were considered. The targets were then classified into four target groups, including (i) with evidence in extending lifespan and same direction of therapeutic inhibition or activation for anti‐aging and anti‐cancer treatment; (ii) with evidence in extending lifespan and opposite direction of therapeutic inhibition or activation for anti‐aging and anti‐cancer treatment; (iii) with evidence in shortening lifespan only; and (iv) without any evidence or no effect in regulating lifespan. Therapeutic approach for each of the targets for cancer treatment was proposed primarily based on their expression changes across the 11 studied cancers, with additional consideration of their mechanism of action suggested by literature to assess whether the proposed approach could deliver anti‐tumor effects. For anti‐aging treatment, the therapeutic approach was proposed by considering their mechanism of action, known longevity‐related pathways and their role in age‐associated diseases according to literature review.

### 2.9 Prediction of potential dual‐purpose candidates based on gene ontology enrichment analysis

GO terms associated with each of the protein‐coding genes were retrieved by R package *biomaRt*. Only the terms belonging to “biological process” were selected. Hypergeometric test was performed using the *enricher* function from R package *clusterProfiler* on the three sets of genes based on their evidence of regulating lifespan: (i) Group 1 and Group 2 (lifespan extension), (ii) Group 3 (lifespan reduction), and (iii) Group 4 (unexplored in lifespan studies) to calculate the statistical significance of enrichment of the genes in each GO term. GO terms with FDR <0.05 were considered as statistically significantly enriched. GO terms enriched in the lifespan‐extending genes (Group 1 and Group 2) were overlapped with those enriched in Group 3 and Group 4 separately. GO terms that were commonly enriched in the lifespan‐extending genes and Group 4 genes only were extracted. Genes associated with these terms were retrieved and assessed for their potential as being dual‐purpose targets based on their mechanisms of action, longevity‐related pathways and their roles in age‐associated diseases.

### 2.10 Lifespan experiment

Standard *C. elegans* strain maintenance procedures were followed in all experiments (Brenner, 1974). Nematode rearing temperature was kept between 18 and 20°C. N2 (wild‐type Bristol isolate) was used in all experiments, and RNAi knockdown of *lsd‐1* (*C. elegans* homolog of the mammalian *KDM1A*) was performed using standard protocol (Fang et al., 2016). The knockdown efficiency was assessed in three biological repeats by comparing the RNA expression level to the control. RNAi‐expressing bacteria were provided by The University of Oslo, Norway. The worms were treated with the *lsd‐1* RNAi‐expressing bacteria or the empty vector L4440 from egg stage onwards. Lifespan examination was initiated at late L4 stage, and performed on Nematode Growth Media plates with 50 μM 5′‐fluorodeoxyuridine (5‐FudR) and seeded with 250 μL of RNAi expressing bacteria. Worms were scored every day and scored as dead when unresponsive to touch. Lifespan experiments were performed with 90 worms/group in three biological repeats. The data from the three biological repeats were pooled into a single Kaplan–Meier plot with *p*‐value calculated using logrank test.

## 3 RESULTS

**FIGURE 1:**
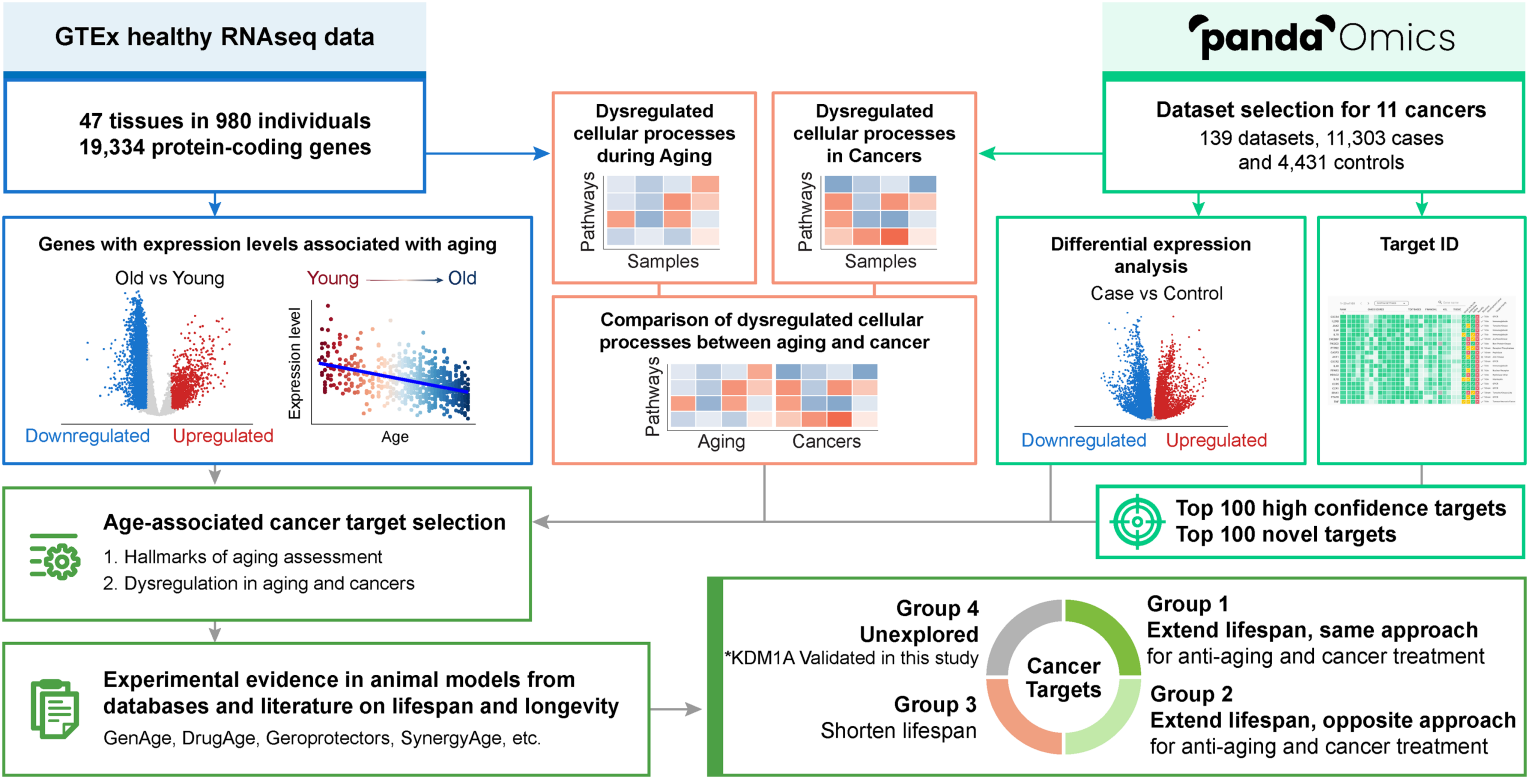
Study workflow. GTEx healthy RNA‐sequencing data for 47 tissues were downloaded and processed. Only protein‐coding genes were extracted and subjected to downstream analyses. Datasets were selected for 11 solid cancers on PandaOmics. For GTEx data, the associations with aging of each gene were assessed by (1) differential expression analysis between old versus young individuals and (2) Pearson's correlation with age. Only those with *p* < 0.05 in both analyses were considered as age‐associated. Differential expression analysis was also performed between the case and control samples for each of the cancer datasets. iPANDA was used to study the dysregulated pathways and cellular processes in the case–control and old‐young comparisons for cancer and GTEx datasets, respectively. Target identification was performed on PandaOmics using the cancer datasets, yielding an overall top 100 targets list for each novelty setting across 11 selected cancers. These targets were then assessed by the involvement in the hallmark(s) of aging, and their differential expression in aging and cancers. The age‐associated cancer targets were selected and classified into four groups, based on the experimental evidence in animal models from databases and literature on lifespan.

The study workflow is illustrated in Figure 1. To identify age‐associated targets, RNA‐sequencing data from 47 healthy tissues of 980 individuals were retrieved from the Genotype‐Tissue Expression (GTEx) database. A total of 19,334 protein‐coding genes were subjected to (1) differential expression analysis between old and young individuals, and (2) correlation analysis between expression level and age (Figure 1, left blue panel). In parallel, to identify cancer‐associated target candidates, we used 139 datasets (131 transcriptomics and 8 proteomics) consisting of 11,303 cases and 4431 control samples selected from 11 age‐associated solid cancers (Table S1). Dysregulated cellular processes were studied across the old‐young and case–control comparisons in GTEx and cancer datasets, respectively (Figure 1, middle orange panel). Target identification was performed by PandaOmics using the cancer datasets, yielding the top 100 high confidence and top 100 novel targets (Figure 1, right green panel). Common pan‐cancer targets were selected based on their occurrence and ranking among the 11 solid cancer types. The age‐associated cancer targets were further selected by (1) their connections to the hallmarks of aging, and (2) dysregulated expression in aging and cancers. The common cancer targets were then classified into four groups based on their association with lifespan extension in several in vivo experimental models, including *Caenorhabditis elegans (C. elegans)*, *Drosophila melanogaster (D. melanogaster)*, and *Mus musculus (M. musculus)*, reported in the existing literature*,* denoting a cluster of genes with dual‐purpose properties. Finally, based on the pathway enrichment analysis of the common cancer targets associated with lifespan extension, mechanisms of action, and evidence of their association with age‐related diseases, we predicted the potential dual‐purpose candidates among those cancer targets that were unexplored in prior lifespan studies. The most promising gene, *KDM1A*, was further validated in the nematode *C. elegans*, further supporting its potential dual‐purpose properties.

### 3.1 Age‐associated genes are tissue‐specific and downregulated with age

**FIGURE 2:**
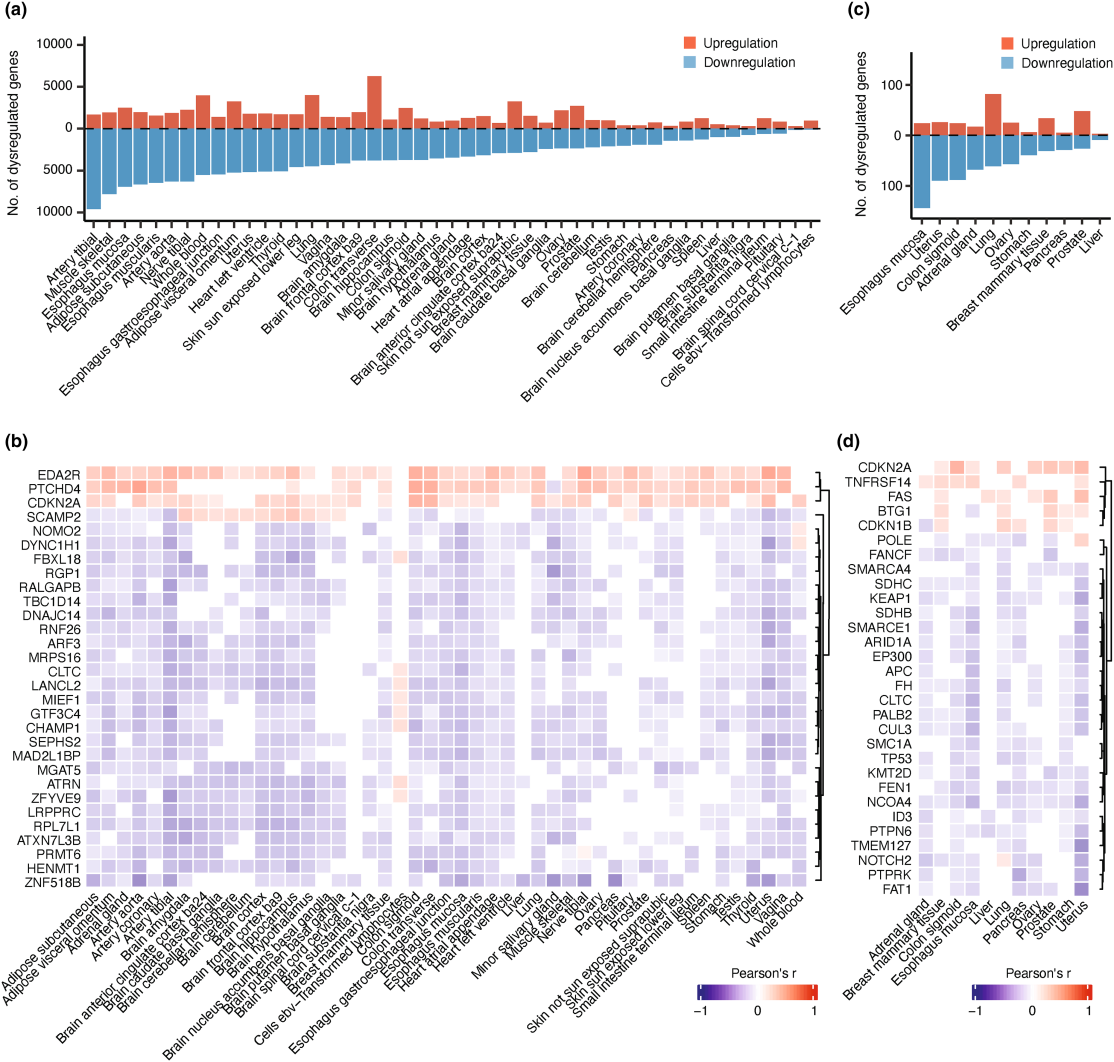
The expression changes of protein‐coding genes and tumor suppressor genes in aging tissue. (a) The number of age‐associated protein‐coding genes in 47 GTEx tissues. Genes were considered dysregulated if they were significantly differentially expressed (*p* < 0.05) in tissues from the old (aged 60–79) compared to the young (aged 20–49) individuals and significantly correlated with age by Pearson's correlation (*p* < 0.05). (b) Correlation between gene expression and age for the top 30 age‐associated protein‐coding genes sorted by the number of tissues and average Pearson's correlation coefficient. (c) The number of age‐associated tumor suppressor genes (TSGs) in 11 selected GTEx tissues. (d) Correlation between gene expression and age for the top 30 age‐associated TSGs sorted by the number of tissues and average Pearson's correlation coefficient.

Obtained from the GTEx database, 16,740 samples from a total of 980 healthy individuals were grouped into 47 tissues, with the sample number ranging from 139 to 803 for each tissue type (Table S1). A total of 19,334 protein‐coding genes were included in the analysis, and their mRNA expression changes with age were studied. Genes were considered age‐associated if their expression level was significantly correlated with age (Pearson's, *p* < 0.05) and upregulated or downregulated (*p* < 0.05) between the old (aged ≥60) and the young (aged <50) individuals. The number of age‐associated genes ranged from 416 to 11,245 (i.e., 2.6% to 73.3%), with an average of 5118 ± 415 (standard error of mean) genes across the 47 studied tissues (Table S2). Notably, more downregulated than upregulated age‐associated genes were observed in 39 tissues, excluding the transverse colon, sun‐unexposed skin, prostate, spleen, ileum, pituitary, lymphocytes, and cervical spinal cord (Figure 2a). The top 30 age‐associated protein‐coding genes sorted by the number of tissues and average Pearson's correlation coefficient are shown in Figure 2b. Taken together, these results highlight the tissue‐specific nature of age‐associated transcriptomics changes and reveal that most of the genes were downregulated with increased age.

### 3.2 Tumor suppressor genes are downregulated during aging

We next selected 11 healthy GTEx tissues to assess the relationship between expression changes of 252 tumor suppressor genes (TSGs, Table S3) with age. While the number of age‐associated TSGs varied among the 11 tissues, their expression levels were mostly downregulated in healthy aging, except for the breast, lung, and prostate tissues (Figure 2c). Esophageal mucosa and lung tissues had the highest number of dysregulated TSGs (*n* = 168, 68.3% and *n* = 143, 57.2%, respectively), while the liver had the lowest (*n* = 12, 4.86%) (Table S2), suggesting the tissue‐specific age‐associated dysregulation patterns for TSGs. The chi‐squared test showed that TSGs demonstrated significantly more age‐associated expression changes when compared to other protein‐coding genes that were age‐associated in at least 1 of the 11 analyzed tissues (*X* ^2^ = 37.82, *p* = 7.76E‐10). The top 30 age‐associated TSGs sorted by the number of tissues and average Pearson's correlation coefficient are summarized in Figure 2d.

In addition to tissue‐specific age‐associated gene expression patterns, we also studied the biological processes involving TSGs during aging. Gene ontology (GO) enrichment analysis revealed the top 20 biological processes enriched by the age‐associated TSGs across 11 tissues. While both upregulated and downregulated TSGs were commonly associated with cell cycle, specifically, only upregulated genes were related to T‐cell proliferation or activation (Figure S1, right panel), whereas downregulated TSGs were associated with a majority of biological pathways related to cell cycle checkpoint and DNA repair mechanisms (Figure S1, left panel). Interestingly, TSG expression changes associated with immune dysregulation, reduced DNA repair ability and cell cycle checkpoint signaling during aging in healthy noncancerous tissues, which may also contribute to increased cancer susceptibility.

### 3.3 Majority of the dysregulated cellular processes are inverted in cancer and healthy aging tissues

**FIGURE 3:**
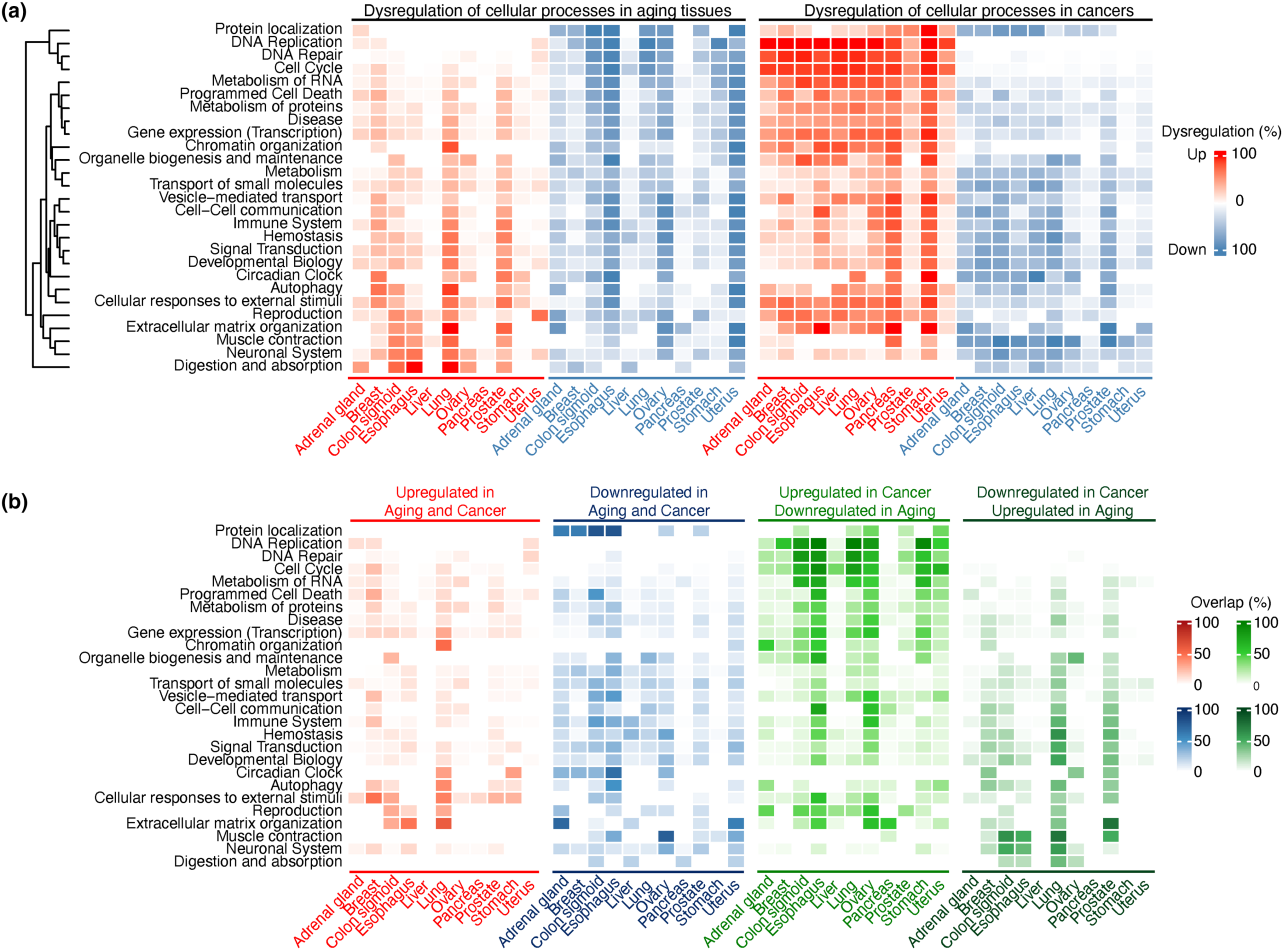
Dysregulated cellular processes in aging and cancer. (a) Overview of dysregulated cellular processes in aging and cancer. The activation or inhibition score for each pathway was calculated with iPANDA by combining precalculated gene coexpression data with gene importance factors based on the degree of differential gene expression and pathway topology decomposition. Pathways with an iPANDA score >0.01 or < −0.01 were considered as dysregulated and extracted. The color intensity represents the percentage of pathways that were upregulated (red) or downregulated (blue) in each cellular process (b) Overlapped dysregulated cellular processes in cancers and aging. The color intensity represents the percentage of the overlapped pathways involved in a cellular process for 11 cancers and their corresponding healthy tissues from GTEx. These cellular processes are (1) upregulated in aging and cancer; (2) downregulated in aging and cancer; (3) upregulated in cancer and downregulated in aging; or (4) downregulated in cancer and upregulated in aging, colored in red, blue, green, and dark green, respectively.

To compare the cellular signaling axes underlying aging and cancer progression, dysregulation of the constituent biological pathways (*n* = 1809) involved in the 27 main cellular processes were analyzed by the In silico Pathway Activation Network Decomposition Analysis (iPANDA) algorithm (Ozerov et al., 2016). While dysregulation of the cellular processes in aging tissues was highly tissue‐specific (Figure 3a, left panel), lung and prostate tissues were enriched with upregulated processes, whereas uterus, ovary, and esophagus were enriched with downregulated processes. Pancreas and liver tissues showed relatively less dysregulated signaling landscape. Notably, over 50% of the pathways involved in protein localization, DNA replication, DNA repair, cell cycle, and RNA metabolism were downregulated in more than half of the GTEx healthy aging tissues (Figure 3a, left panel). In contrast, these cellular processes as well as programmed cell death, metabolism of proteins, disease, gene expression (transcription), chromatin organization, cellular response to external stimuli, reproduction, and extracellular matrix organization were upregulated in at least five cancer tissues (Figure 3a, right panel).

A further analysis of the biological pathways dysregulated in both aging and cancer tissues revealed that DNA replication, DNA repair, and cell cycle were commonly upregulated in cancer but downregulated in aging across all tissues. Such pattern was dominant across a majority of the overlapped dysregulated processes, followed by pathways that are downregulated in both aging and cancer (Figure 3b). On average, 18.5% of all pathways were upregulated in cancer and downregulated in aging, and 12.0% were downregulated in both tissues (Table S4). Notably, such dysregulation patterns were tissue‐specific. For example, most of the dysregulated cellular processes were downregulated in cancer but upregulated in aging prostate tissue, while no dominant dysregulation trends were observed in breast tissue.

### 3.4 Pathways regulating cellular senescence are upregulated in both aging and cancer

Among all unidirectionally dysregulated signaling processes, pathways associated with cellular responses to external stimuli (Figure 3b) such as cellular senescence (R‐HSA‐2559583), including oncogene‐induced senescence (R‐HSA‐2559585) and oxidative stress‐induced senescence (R‐HSA‐2559580), were the most upregulated axes in both aging and cancer tissues (Table S5). On the contrary, signal transduction pathways associated with GPCR (R‐HSA‐388396), EGFR (R‐HSA‐177929), and MAPK1/MAPK3 signaling (R‐HSA‐5684996) were predominantly downregulated in both aging and cancer (Figure 3b and Table S5).

### 3.5 The identification of age‐associated common cancer targets

**FIGURE 4:**
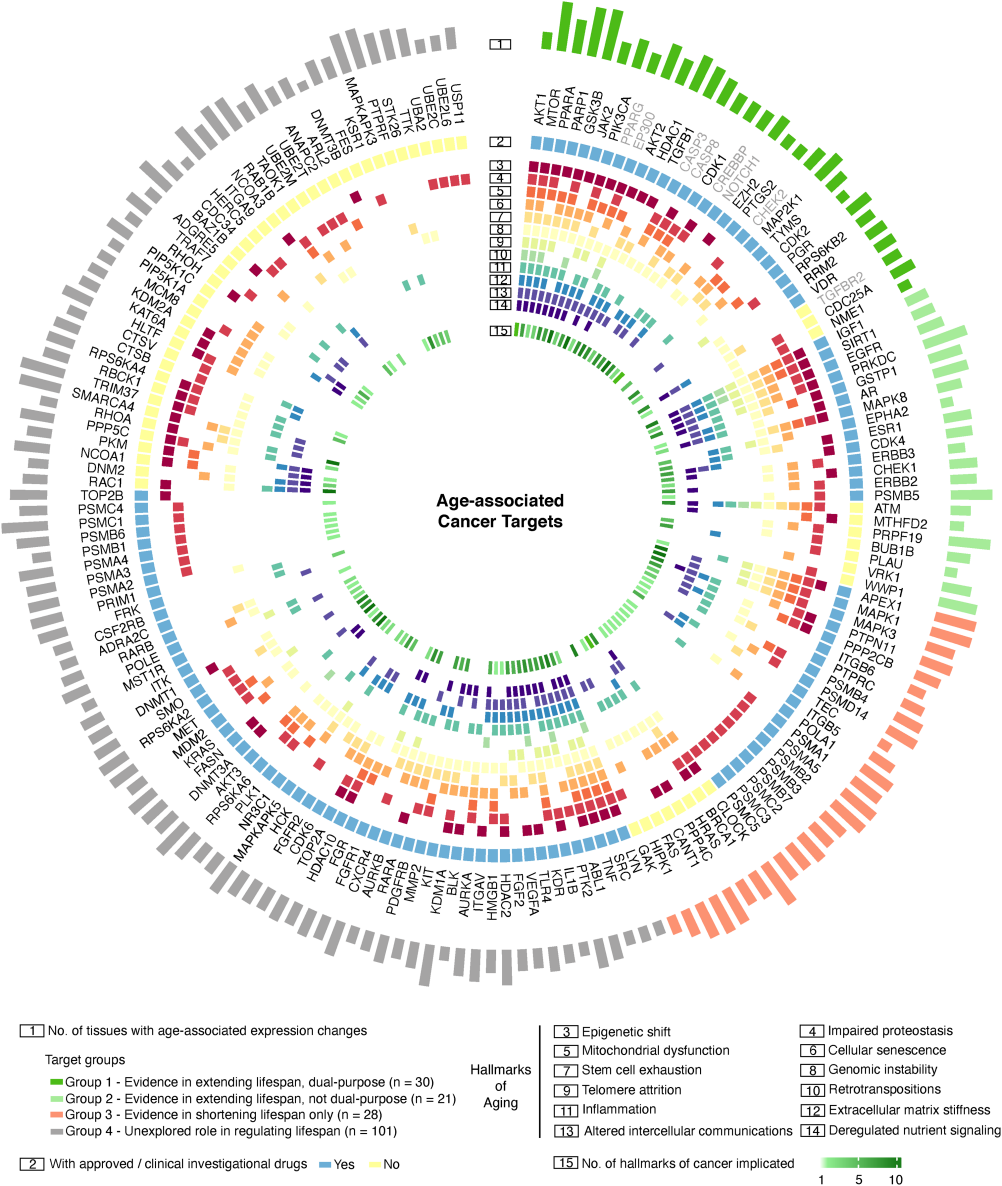
Age‐associated cancer targets and dual‐purpose targets. Targets were selected from the top 100 high confidence and 100 novel common cancer targets and were filtered based on the number of tissues dysregulated during aging and hallmarks of aging associated. They were then classified into four target groups by experimental evidence demonstrating their roles in regulating lifespan. A total of 180 targets were involved in this target wheel. The association of targets with 12 aging hallmarks was based on Gene Ontology terms selected according to Lopez‐Otin et al. (2013) and Pun, Leung, et al. (2022). The association of targets with 10 cancer hallmarks was based on the COSMIC and CHG databases. Tumor suppressor genes are labeled in gray.

To identify promising druggable targets implicated in cancers, we applied multiple AI omics and text models integrated in PandaOmics (see Methods for detailed description). In brief, the AI omics models including network neighbors, causal inference, pathways, interactome community, relevance, expression, heterogeneous graph walk, and matrix factorization were used to prioritize the targets based on omics data analyses. Text‐based models were used to study the target‐disease associations based on publications, attention, and grant funding. The targets were classified into two main groups, high confidence and novel targets, based on the proprietary AI engine. High confidence targets are well‐studied and reported in the specific disease area with voluminous publications, while novel targets are less studied and mentioned in the literature. The top 100 AI‐derived targets for each cancer type yielded a pool of 301 high confidence and 319 novel druggable targets across 11 age‐associated solid cancers. Based on the occurrence frequency and average ranking of these targets, the top 100 high confidence (Figure S2a) and top 100 novel (Figure S3a) common cancer targets were further selected from the target pool. Notably, most of the common targets were upregulated among the solid malignancies (Figure S2a). Next, the age‐associated expression changes of these common cancer targets across the 47 GTEx healthy tissues were analyzed (Figure S2b, Figure S3b), and genes dysregulated in more than 10% (*n* = 5) of GTEx tissues and associated with hallmarks of aging were considered as age‐associated cancer targets. A total of 180 such targets were identified, of which, 119 were associated with FDA‐approved or clinical investigational drugs (Table S6). The anti‐cancer therapeutic approach for these targets was proposed primarily based on expression change across the majority of analyzed cancers, with additional consideration of the target's mechanism of action suggested by literature. The target wheel (Figure 4) provides detailed information for each of the 180 age‐associated common cancer targets, including the number of tissues with age‐associated expression changes, development level, and connection to the hallmarks of aging and hallmarks of cancer. Interestingly, age‐associated expression changes in *MTOR* and *GSK3B* genes were noted in the highest number of GTEx healthy tissues. In addition, four targets (*AKT1, MTOR, IGF1,* and *SIRT1*) were associated with all aging hallmarks. The top 5 hallmarks of aging with the highest number of age‐associated cancer targets were genomic instability (*n* = 114), impaired proteostasis (*n* = 95), cellular senescence (*n* = 78), inflammation (*n* = 75), and altered intercellular communications (*n* = 70).

### 3.6 Classification of age‐associated cancer targets

In order to identify potential anti‐aging targets among the 180 age‐associated cancer targets, we systematically reviewed the evidence demonstrating the effects of these genes in regulating lifespan from several in vivo experimental models (*C. elegans*, *D. melanogaster,* and *M. musculus*), using publicly available curated databases such as DrugAge (database of aging‐related drugs), GenAge (aging gene database), Geroprotectors (database of geroprotectors), and SynergyAge (database for synergistic and antagonistic interactions of longevity‐associated genes). The 180 age‐associated cancer targets were classified into four groups (Figure 4; Table S6). Specifically: Group 1—dual‐purpose targets with evidence in extending lifespan and same direction of therapeutic inhibition or activation for anti‐aging and anti‐cancer treatment (*n* = 30, Table 1); Group 2—age‐associated cancer targets with evidence in extending lifespan and opposite direction of therapeutic inhibition or activation for anti‐aging and anti‐cancer treatment (*n* = 21); Group 3—age‐associated cancer targets with evidence in shortening lifespan only (*n* = 28); and Group 4—age‐associated cancer targets unexplored in lifespan studies (*n* = 101) (Figure 4, Table S7).

**TABLE 1:** Proposed dual‐purpose targets for anti‐aging and anti‐cancer treatment.

Dual‐purpose targets^a^	Therapeutic approaches^b^	Current clinical status^c^	Target‐associated FDA‐approved drug/investigational drug for cancer	Aging			Number of aging hallmarks	Role in modulating longevity^d^	References^e^
				Number of dysregulation	Number of upregulation	Number of downregulation			
GSK3B	Antagonism	Clinical investigation	9‐ING‐41 (advanced cancer), LY‐2090314 (leukemia, pancreatic cancer)	33	0	33	9	Pro‐aging	NCT03678883, NCT01214603, NCT01632306
MTOR	Antagonism	FDA approval	Everolimus (gastrointestinal or lung origin with unresectable, locally advanced or metastatic disease), Temsirolimus (renal cell carcinoma)	32	0	32	12	Pro‐aging	3893024* 4238713*
PARP1	Antagonism	FDA approval	Olaparib (ovarian cancer, breast cancer, pancreatic cancer, prostate cancer Rucaparib (ovarian cancer, prostate cancer) Niraparib (advanced epithelial ovarian, fallopian tube, or primary peritoneal cancer) Talazoparib (BRCA‐mutated HER2‐negative advanced or metastatic breast cancer)	32	1	31	9	Pro‐aging	4610866* 4997677* 4600207* 4335341*
HDAC1	Antagonism	FDA approval	Vorinostat (cutaneous T‐cell lymphoma), Romidepsin (cutaneous T‐cell lymphoma)	24	6	18	7	Pro‐aging	3043460* 4834454*
PPARA	Agonism	FDA approval*	Fenofibrate (multiple myeloma), Gemfibrozil (cancer)	22	1	21	11	Anti‐aging	NCT01965834, NCT01340846
TGFB1	Antagonism	FDA approval*	M7824 (pancreatic cancer, cervical cancer), Fresolimumab (breast cancer, lung cancer), LY‐2382770 (non‐Hodgkins, breast cancer)	21	18	3	7	Pro‐aging	NCT03451773, NCT03427411, NCT01401062, NCT02581787, NCT02018874, NCT01980277
MAP2K1	Antagonism	Clinical investigation	Pimasertib (advanced solid tumor)	20	0	20	3	Pro‐aging	NCT01378377
PIK3CA	Antagonism	FDA approval	Copanlisib (follicular lymphoma), Alpelisib (breast cancer)	19	3	16	9	Pro‐aging	4152629* 4438804*
AKT2	Antagonism	Clinical investigation	Capivasertib (breast cancer, prostate cancer), Ipatasertib (breast cancer, prostate cancer), MK2206 (breast cancer, non‐small cell lung cancer), Uprosertib (breast cancer, melanoma)	17	5	12	7	Pro‐aging	NCT04177108, NCT03072238, NCT03742102, NCT02077569, NCT01964924, NCT01941927, NCT01277757, NCT01294306
PGR	Agonism	FDA approval*	Mifepristone (endometrial cancer, ovarian cancer)	17	12	5	2	Anti‐aging	NCT00505739, NCT00459290
CDC25A	Antagonism	Preclinical study	‐	16	1	15	1	Pro‐aging	‐
JAK2	Antagonism*	FDA approval	Ruxolitinib (myelofibrosis)	14	3	11	9	Pro‐aging	4860976*
RRM2	Antagonism	FDA approval*	Hydroxyurea (head and neck cancer, gastric cancer), Motexafin Gadolinium (kidney cancer, lung cancer)	14	2	12	1	Pro‐aging	NCT00117572, NCT00003172, NCT00717587, NCT00373204
TYMS	Antagonism	FDA approval	Trifluridine (colorectal cancer), Pemetrexed (lung cancer, mesothelioma)	14	8	6	3	Pro‐aging	3823053* 4165466*
VDR	Agonism*	FDA approval*	Cholecalciferol (breast cancer), Ergocalciferol (breast cancer, prostate cancer), Paricalcitol (pancreatic cancer)	14	1	13	1	Anti‐aging	NCT02786875, NCT01224678, NCT00079001, NCT03331562
CDK1	Antagonism	Clinical investigation	Dinaciclib (leukemia), Alvocidib (multiple myeloma, liver cancer)	13	4	9	6	Pro‐aging	NCT01580228, NCT00047203, NCT00087282
RPS6KB2	Antagonism	Clinical investigation	LY‐2780301 (lymphoma, metastatic cancer) MSC‐2363318A (neoplasm)	12	1	11	2	Pro‐aging	NCT02018874, NCT01115751, NCT01971515
AKT1	Antagonism	Clinical investigation	Capivasertib (breast cancer, prostate cancer), Ipatasertib (breast cancer, prostate cancer), MK2206 (breast cancer, non‐small cell lung cancer), Uprosertib (breast cancer, melanoma)	11	1	10	12	Pro‐aging	NCT04177108, NCT03072238, NCT03742102, NCT02077569, NCT01964924, NCT01941927, NCT01277757, NCT01294306
EZH2	Antagonism	FDA approval	Tazemetostat (follicular lymphoma)	9	3	6	4	Pro‐aging	4627347*
PTGS2	Antagonism*	Clinical investigation	Celecoxib (breast cancer) Aspirin (prostate cancer)	9	8	1	4	Pro‐aging	NCT00525096, NCT02429427, NCT00316927
NME1	Antagonism	Preclinical study	‐	8	0	8	2	Pro‐aging	‐
CDK2	Antagonism	Clinical investigation	Dinaciclib (leukemia), Alvocidib (multiple myeloma, liver cancer), UCN‐01 (pancreatic cancer, lung cancer)	7	4	3	2	Pro‐aging	NCT01580228, NCT00047203, NCT00087282, NCT00045747, NCT00098956

^a^ Thirty dual‐purpose targets were selected from the top 100 high confidence and top 100 novel common cancer targets. Among them, eight tumor suppressor genes (CASP3, CASP8, CHEK2, CREBBP, EP300, NOTCH1, PPARG, and TGFBR2) were excluded.

^b^ Generally upregulation in expression led to target antagonist for cancer; downregulation in expression led to target agonist for cancer, according to our analyzed cancer datasets. The therapeutic approach proposed solely based on target's mechanism of action was marked with an asterisk.

^c^ Targets that are clinically investigated only for oncology indications were marked with an asterisk.

^d^ The effects of target genes on modulating longevity were determined by increasing or decreasing lifespan from in vivo studies (see Table S6).

^e^ ClinicalTrials.gov identifier or *FDA Reference ID for target‐associated FDA‐approved drug/investigational drug (indications for cancers) was shown.

### 3.7 Proposed dual‐purpose targets

**FIGURE 5:**
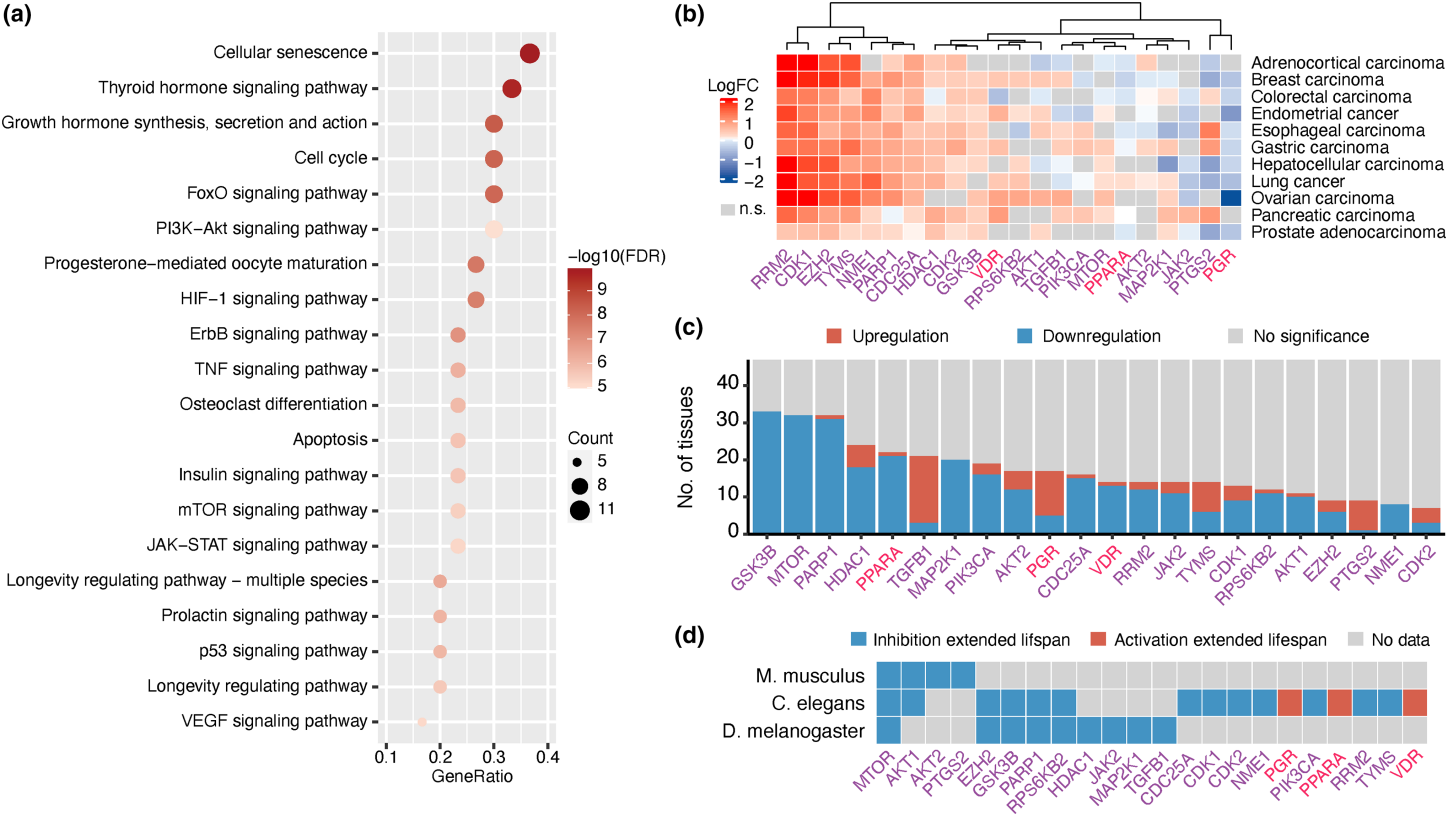
Overview of the identified dual‐purpose targets. (a) Top 20 KEGG pathways significantly enriched with the identified dual‐purpose (Group 1) targets. Sixty‐three pathways defined as human diseases from KEGG that were significantly enriched were excluded. (b) The dysregulated expression of 22 dual‐purpose targets in 11 studied cancers. Targets with non‐significant (n.s.; FDR≥0.05) LogFC are colored in gray. Therapeutic antagonism for anti‐aging and anti‐cancer treatment was proposed for most of the targets (genes in purple), while agonism was proposed for *PPARP, PGR,* and *VDR* (genes in red). (c) The number of tissues dysregulated in 47 GTEx tissues during aging for the 22 dual‐purpose targets. (d) Evidence of lifespan extension in different animal models for these dual‐purpose targets.

KEGG pathway enrichment analysis revealed that cellular senescence and other signaling pathways such as FoxO, PI3K‐AKT, HIF‐1, ErbB, TNF, insulin, mTOR, JAK–STAT, prolactin, p53, and VEGF were significantly enriched among the 30 dual‐purpose targets in Group 1 (FDR <0.05; Figure 5a). Eight targets (*EP300, CASP3, CASP8, CHEK2, CREBBP, NOTCH1, PPARG,* and *TGFBR2*) were TSGs (Figure 4, gray text), suggesting that antagonizing these molecules might increase the susceptibility to cancer in healthy individuals. The 22 remaining dual‐purpose targets are summarized in Table 1, providing detailed information on the proposed therapeutic approaches based on their mechanisms of action, clinical trial status, dysregulation in GTEx tissues, connection to the hallmarks of aging, and role in modulating longevity. These targets, except *PGR,* were overexpressed in at least one cancer type (Figure 5b)*,* while most of these genes were predominantly downregulated in aging GTEx tissues (Figure 5c). Antagonism, for most of the selected targets (Figure 5b–c, genes in purple), and agonism, for PGR, PPARA, and VDR (genes in red), were proposed as potential approaches for anti‐aging and anti‐cancer treatment. Of note, while VDR was mostly upregulated among cancer types, higher expression of VDR is suggested to exert an anti‐tumorigenic effect and associated with favorable prognosis in multiple types of cancer (Campbell & Trump, 2017; Yu et al., 2023). As such, agonism was proposed as a putative therapeutic approach. Most research on lifespan extension has been conducted in classical model species, such as *C. elegans*, *D. melanogaster,* and *M. musculus*. Inhibition, knockdown, or knockout experiments on *MTOR*, *AKT1*, *AKT2*, *PTGS2*, *EZH2*, *GSK3B*, *PARP1*, and *RPS6KB2* demonstrated their role in lifespan extension using either mammalian or at least two types of invertebrate animal models (Figure 5d, Table S6). FDA‐approved inhibitors are available for mTOR, PARP1, and EZH2, while the remaining five target candidates are currently being investigated in clinical trials (Table 1). Based on the reported validation in animal models, *MTOR, AKT1, PARP1, AKT2, PTGS2, GSK3B, EZH2*, and *RPS6KB2* were considered priority targets for further anti‐aging investigation.

### 3.8 Prediction of potential dual‐purpose candidates among genes with unexplored effects on lifespan

**FIGURE 6:**
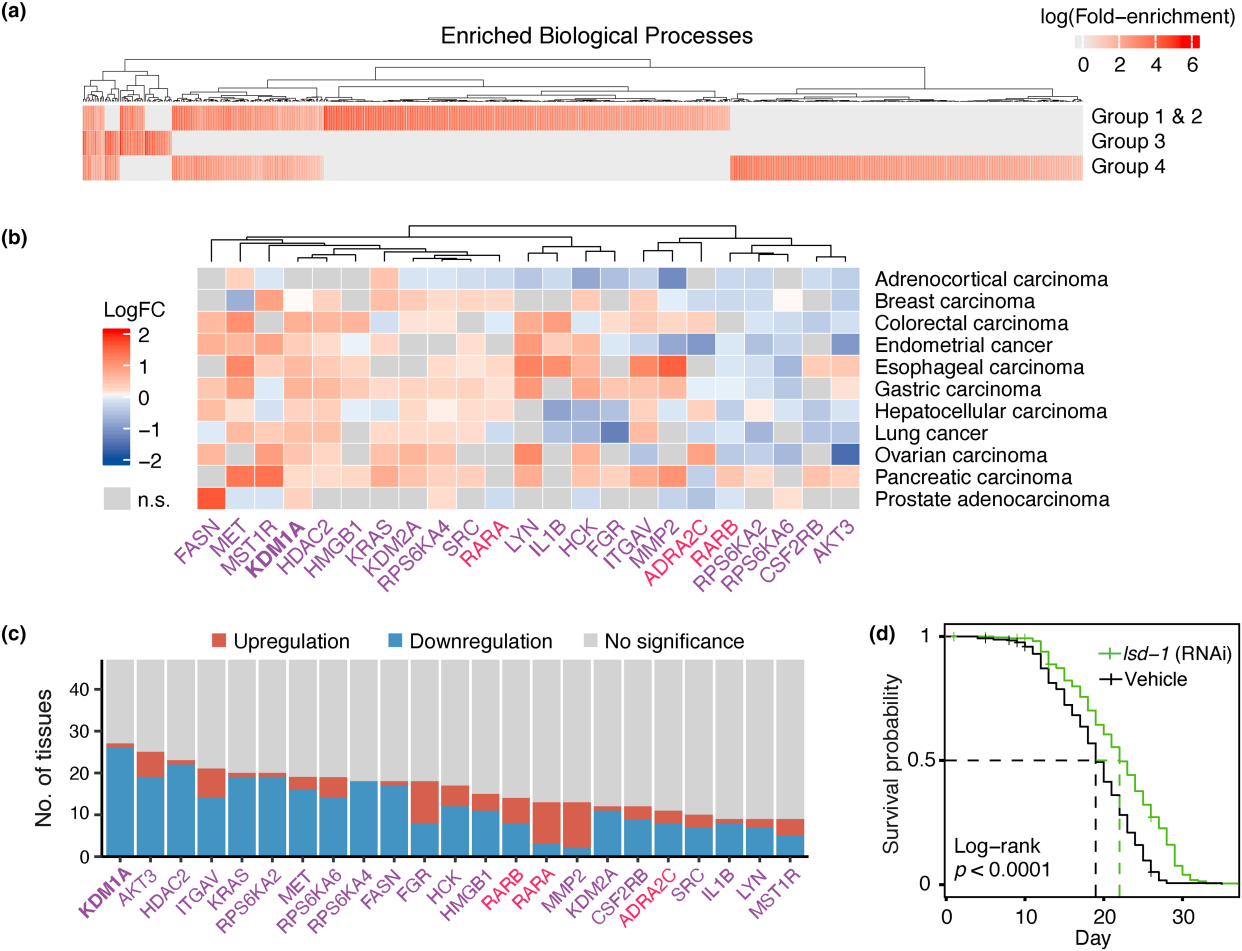
Overview of the predicted potential dual‐purpose candidates. (a) Gene ontology (biological processes) enrichment analysis was performed on targets associated with lifespan extension (Group 1 and Group 2), Group 3 and Group 4 targets independently to identify the commonly enriched pathways between only the lifespan‐extending and Group 4 targets. (b) The dysregulated expression of 23 potential dual‐purpose candidates in 11 studied cancers. Therapeutic antagonism for anti‐aging and anti‐cancer treatment was proposed for most of the targets (genes in purple), while agonism was proposed for *RARA, RARB,* and *ADRA2C* (genes in red). (c) The number of tissues dysregulated during aging for 23 potential dual‐purpose candidates. (d) *KDM1A* was selected for lifespan experiments. Kaplan–Meier survival curves for *C. elegans* treated with either the *lsd‐1* (homolog of the mammalian *KDM1A*) RNAi‐expressing bacteria (green, *n* = 270) or the empty vector (black, *n* = 270).

To predict the potential dual‐purpose candidates among targets classified to Group 4 (genes unexplored in lifespan studies), hypergeometric test was performed to compare the association between the biological processes enriched in Group 4 and those enriched in Group 1 and Group 2 (containing targets which are associated with lifespan extension) (Figure 6a). Ninety overlapped biological processes were identified as demonstrated in Table S8. Seventy‐nine targets from Group 4 were involved in these overlapping biological processes (Table S7). Among those, 23 targets (*KDM1A, AKT3, HDAC2, ITGAV, KRAS, RPS6KA2, MET, RPS6KA6, RPS6KA4, FASN, FGR, HCK, HMGB1, RARB, RARA, MMP2, KDM2A, CSF2RB, ADRA2C, SRC, IL1B, LYN,* and *MST1R*) were further selected as potential dual‐purpose candidates based on their mechanisms of action and evidence of their role in age‐associated diseases (Figure 6b). Antagonism, for most of the selected targets (genes in purple), and agonism, for *RARA, RARB*, and *ADRA2C* (genes in red), were proposed as potential approaches for anti‐aging and anti**‐**cancer treatment. The 23 potential dual‐purpose candidates are summarized in Table 2, providing detailed information on the proposed therapeutic approaches based on their mechanisms of action, clinical trial status, dysregulation in GTEx tissues, and connection to the hallmarks of aging. Most of these targets were associated with FDA‐approved or investigational drugs, except *RPS6KA4* and *KDM2A*. All potential dual‐purpose candidates were overexpressed in at least one cancer type (Figure 6b)*,* while 20 of these genes were predominantly downregulated in aging GTEx tissues (Figure 6c).

**TABLE 2:** Predicted potential dual‐purpose candidates for anti‐aging and anti‐cancer treatment.

Potential dual‐purpose candidates^a^	Therapeutic approach^b^	Current clinical status^c^	Aging			Number of aging hallmarks
			Number of dysregulation	Number of upregulation	Number of downregulation	
KDM1A	Antagonism	Clinical investigation	27	1	26	6
AKT3	Antagonism	FDA approval	25	6	19	3
HDAC2	Antagonism	FDA approval	23	1	22	7
ITGAV	Antagonism	Clinical investigation	21	7	14	7
RPS6KA2	Antagonism	Clinical investigation	20	1	19	3
KRAS	Antagonism	FDA approval	20	1	19	3
MET	Antagonism	FDA approval	19	3	16	3
RPS6KA6	Antagonism	Clinical investigation	19	5	14	4
RPS6KA4	Antagonism	Preclinical study	18	0	18	4
FGR	Antagonism	FDA approval	18	10	8	5
FASN	Antagonism	Clinical investigation	18	1	17	3
HCK	Antagonism	FDA approval	17	5	12	4
HMGB1	Antagonism	Clinical investigation	15	4	11	7
RARB	Agonism	Clinical investigation	14	6	8	2
MMP2	Antagonism	Clinical investigation	13	11	2	6
RARA	Agonism	Clinical investigation	13	10	3	6
CSF2RB	Antagonism	Clinical investigation*	12	3	9	1
KDM2A	Antagonism	Preclinical study	12	1	11	3
ADRA2C	Agonism	FDA approval*	11	3	8	1
SRC	Antagonism	FDA approval	10	3	7	11
LYN	Antagonism	FDA approval	9	2	7	11
IL1B	Antagonism	Clinical investigation	9	1	8	8
MST1R	Antagonism	Clinical investigation	9	4	5	2

^a^ Group‐4 targets involved in biological processes enriched with Group 1 and Group 2 targets.

^b^ The therapeutic approach for cancer was based on expression in cancer datasets and the target's mechanism of action (see Table S6 for details). The therapeutic approach for anti‐aging was based on mechanism of action, known longevity‐related pathways and the role in age‐associated diseases (see Table S7 for details).

^c^ *ADRA2C was associated with clinical investigational drugs for oncology indications. CSF2RB was pre‐clinically studied for oncology indications.

### 3.9 KDM1A: A promising dual‐purpose candidate

Among the 23 predicted dual‐purpose candidates, the expression of *KDM1A* (lysine‐specific demethylase 1A) was negatively correlated and significantly reduced with age in 26 GTEx healthy aging tissues (Table S6) including artery, esophagus, minor salivary gland, muscle skeletal, testis, thyroid, and vagina among others (*p* < 0.05, Pearson's correlation; *p* < 0.05, LogFC; Figure S4). In addition, *KDM1A* was significantly upregulated in 10 of 11 cancer types (Figure 6b). Given its potential involvement in carcinogenesis, targeting KDM1A is becoming an emerging option for the treatment of cancer (Li et al., 2021), and numerous KDM1A inhibitors are currently undergoing clinical assessment for the treatment of both solid and hematopoietic malignancies (Y. Fang et al., 2019; Karakaidos et al., 2019). Notably, RNA interference‐mediated knockdown of *lsd‐1*, the *C. elegans* homolog of the mammalian *KDM1A*, significantly increased the median lifespan by 15.8% (*p* < 0.0001) compared to worms fed with bacteria expressing the empty vector (22 ± 0.36 vs. 19 ± 0.33 days) (Figure 6d **,** Figure S5) in experiments using 90 5′‐fluorodeoxyuridine treated animals per group in three biological repeats, supporting the potential dual‐purpose properties of KDM1A. Taken together, we propose KDM1A as a potential dual‐purpose target worthy of further investigation in long‐lived model organisms.

## 4 DISCUSSION

Pervasive downregulation of genes that regulate a wide range of cellular processes, including mitochondrial function, immune response, growth factor signaling, and DNA repair, is suggested to be correlative with aging (Frenk & Houseley, 2018). While our results support this suggestion by demonstrating that most genes, including tumor suppressors, are downregulated during aging, it remains uncertain whether these changes may cause functional decline or provide anti‐aging protection. On the contrary, the hyper‐functional theory of aging hypothesizes that even though many of the critical genes are downregulated during aging, they retain sufficient activities for functional integrity, that is, hyper‐functional (Blagosklonny, 2022). For example, while *MTOR* gene expression is negatively correlated with age, additional inhibition could increase lifespan in vivo (Papadopoli et al., 2019), supporting a rationale that further inhibition of the targets downregulated in aging could also improve longevity.

Many biological processes are countered in aging and tumorigenesis, as pathways inactivated during aging (such as cell cycle and DNA replication) were often upregulated during carcinogenesis (Sedrak & Cohen, 2022). Therefore, signaling molecules involved in these cellular processes are unlikely to be dual‐purpose targets. Alternatively, cellular senescence‐related pathways are upregulated in both aging and cancer. Inhibiting the targets involved in these pathways may delay aging and combat cancer. Fifteen of the proposed dual‐purpose targets classified to Group 1 (*AKT1, CASP3, CDK1, CDK2, CHEK2, CREBBP, EP300, EZH2, GSK3B, HDAC1, JAK2, MAP2K1, MTOR, PARP1*, and *PIK3CA*) are linked to cellular senescence. In addition, known targets for cancer prevention and therapy such as mitogenic and survival signaling pathways driven by Ras, MEK, PI3K, mTOR, Akt, and NFκB are also involved in signaling pathways underlying the aging process. Subsequently, antagonizing these targets may potentially delay aging while inhibiting the onset of malignant disorders (Papadopoli et al., 2019).

Our study has demonstrated that aging hallmarks such as cellular senescence, inflammation, and impaired proteostasis are strongly correlated with multiple age‐associated malignancies. Notably, cellular senescence was the top pathway enriched for the proposed dual‐purpose targets. It was reported that cellular senescence signatures are activated in aging tissues (Chatsirisupachai et al., 2019), and that removal of the senescent cells can reduce age‐related tissue dysfunction, increase healthspan, and delay aging (Kumari & Jat, 2021). For instance, senolytics such as dasatinib and quercetin can reduce senescence and induce beneficial effects on chronic age‐associated conditions in vivo (Raffaele & Vinciguerra, 2022). While senescence may inhibit the tumorigenesis to a certain extent, cumulative evidence strongly support its role in promoting the angiogenesis to advance tumor growth, as well as facilitating invasion and metastasis of the cancer cells through a hypersecretory senescence‐associated secretory phenotype (SASP) (Collado et al., 2007). Thus, modulating cellular senescence may be beneficial for both delaying aging and treating a proliferative disorder. Inflammation, a common biological consequence of cellular senescence and autophagy, is also highly associated with multiple age‐related neurological, metabolic, fibrotic, and inflammatory diseases (Pun, Leung, et al., 2022). Chronic inflammation and tissue damage are frequently seen in aged individuals and were shown to promote cancer cell proliferation, survival, and migration by triggering the release of pro‐inflammatory cytokines and chemokines (Greten & Grivennikov, 2019). As such, immune modulatory interventions aiming at rescuing a dysregulated inflammatory response in order to facilitate tissue repair and maintain homeostasis are essential. Autophagy, a highly conserved lysosome‐mediated cellular degradation, plays an important role in proteostasis maintenance by removing toxic cytosolic protein aggregates and damaged cellular organelles (Lopez‐Otin, Blasco, et al., 2023). “Compromised autophagy” is considered a new hallmark of aging, underlying all age‐associated diseases (Schmauck‐Medina et al., 2022). The autophagic activity decreases with age, leading to an accumulation of damaged macromolecules and organelles (Barbosa et al., 2018). Inhibition of senescence through the activation of autophagy was shown to induce beneficial effects on skin aging (Lim et al., 2017). While the biological effects of autophagy in cancer are not fully understood, basal autophagy is considered to mediate tumor suppression, whereas a high level was shown to promote oncogenesis (Yun & Lee, 2018). Although the mechanisms that explain this paradox are not yet elucidated, therapeutic approaches targeting senescence, suppressing inflammation, and modulating autophagy could provide promising anti‐aging and anti‐cancer intervention strategies (Yun & Lee, 2018).

In this study, we identified 180 age‐associated common cancer targets and classified them into four groups based on the evidence demonstrating their effects in regulating lifespan in vivo. Notably, Group 1 contained promising dual‐purpose targets such as *MTOR, AKT1, PTGS2,* and *GSK3B*. *MTOR* is a well‐known dual‐purpose target. Everolimus and temsirolimus, mTOR inhibitors, have been approved by the FDA for the treatment of gastrointestinal, lung, and renal cancers, whereas rapamycin, another mTOR inhibitor, increased the median lifespan of genetically heterogeneous mice by approximately 25% (in both sexes, Miller et al., 2014), and its impact on aging has been investigated in clinical trial setting (NCT04488601, NCT03072485). AKT1, RAC‐alpha serine/threonine‐protein kinase, regulates many cellular processes such as metabolism, proliferation, cell survival, growth, and angiogenesis. AKT1 inhibitors, capivasertib, ipatasertib, MK2206, and uprosertib, have been clinically investigated for treating multiple solid malignancies with promising results. On the contrary, a deletion mutation in akt‐1 or heterogeneous knockout mutants increased lifespan in vivo (Nojima et al., 2013), supporting its selection as a potential dual‐purpose target. In addition, PTGS2 inhibitors, a key enzyme in prostaglandin biosynthesis, resulted in decreased recurrence and mortality in patients with breast (NCT00525096, NCT02429427) and prostate cancer (NCT00316927). Notably, PTGS2 inhibition was reported to extend lifespan and modulate skin aging, while its transgenic expression caused premature aging phenotype in mice (Lee et al., 2012; Strong et al., 2008). Moreover, inhibition of GSK3B, a constitutively active protein kinase that acts as a negative regulator of glucose homeostasis, Wnt signaling, and regulation of transcription factors and microtubules have been clinically investigated in advanced solid malignancies (NCT03678883) and leukemia (NCT01214603, NCT01632306), yielding promising results (Park et al., 2021). In parallel, longevity studies showed that GSK3B downregulation is associated with a significant lifespan increase in vivo (Castillo‐Quan et al., 2016). Remarkably, while GSK3B inhibition suppresses senescence signaling and delays kidney aging in aged mice (Fang et al., 2022), a complete absence of GSK3B may shorten the lifespan or impair development (Castillo‐Quan et al., 2016).

In addition to the proposed dual‐purpose targets classified into Group 1, we predicted 23 potential dual‐purpose candidates that were previously unexplored in longevity research based on multiple lines of evidence from public databases. Among these candidates, *KDM1A* was dysregulated in the highest number of tissues during aging. Therefore, its effect on lifespan was further evaluated in vivo. Knockdown of *lsd‐1* (homolog of the mammalian *KDM1A*) significantly increased the lifespan in wild‐type *C. elegans* animals, supporting a role of KDM1A in longevity. Despite the downregulated pattern of *KDM1A* expression was observed across the GTEx tissues of aged (≥60) individuals, the effect of KDM1A inhibition on lifespan extension in C. *elegans* suggests that the age‐related reduction in *KDM1A* expression could be a protective mechanism. This is supported by previous studies measuring age‐associated expression changes of the orthologs of *KDM1A* in *C. elegans* (Wang et al., 2022) and *M. musculus* (Duteil et al., 2017), showing that its expression is downregulated during aging across multiple tissues. Cumulative evidence indicates that KDM1A is an essential regulator of autophagy, and that its inhibition can enhance the autophagosomal formation and stimulate autophagic flux via the mTOR signaling pathway (Wei et al., 2018). Consistently, modulation of KDM1A with vafidemstat (a selective inhibitor also known as ORY‐2001) rescues the age‐associated neurodegeneration in mice (Maes et al., 2020). In addition to its anti‐aging effect, KDM1A inhibitor IMG‐7289 has been clinically investigated for treatment of solid and hematologic malignancies (NCT02842827, NCT05191797), while preclinical studies have demonstrated its role in the regulation of stemness in different myeloid and solid cancers (Karakaidos et al., 2019), supporting the status of KDM1A as a promising dual‐purpose target candidate for anti‐aging and anti‐cancer treatment. Nevertheless, additional studies using nematodes and other animal models are warranted to further decipher the function of KDM1A and to experimentally confirm the role and other less‐explored dual‐purpose targets proposed by our study in both cancers and aging.

Development of improved approaches capable of identifying potential dual‐purpose targets for aging and cancer would enable biologists and clinicians to further investigate their therapeutic potential in a cost‐saving and time‐efficient manner. The next steps toward implementation of these targets into the clinic would involve a generation of small molecules and their validation in both preclinical testing and clinical trial setting to determine their safety, efficacy, and potential side effects. As our results underscore the ability of PandaOmics to identify dual‐purpose targets across multiple types of diseases, the potential clinical utility of this AI‐driven platform in augmenting healthy aging is broad.

## 5 CONCLUSION

In this study, we have applied PandaOmics, a comprehensive artificial intelligence target discovery platform, to analyze large omics datasets containing thousands of patients with various solid malignancies (derived from public repositories) and healthy tissues obtained from GTEx, for identifying potential targets that can be used for both anti‐aging and anti‐cancer treatment. Using this unique approach, we were able to identify several well‐known anti‐aging drug targets as potential dual‐purpose candidates and proposed new targets that were not previously associated with lifespan extension. We further confirmed the validity of our target prediction model by demonstrating the effect of KDM1A (one of the unexplored candidates) on lifespan extension in vivo, supporting the effectiveness of this approach. To date, this is the first study demonstrating the feasibility of an AI‐driven innovative approach to identify dual‐purpose target candidates for anti‐aging and anti‐cancer treatment. The in vivo validation clearly demonstrates the value of integrative AI‐driven tools in addressing the complex challenges at the interface between aging and carcinogenesis.

## AUTHOR CONTRIBUTIONS

F.W.P. analyzed data, participated in result interpretation and project administration, and drafted the manuscript. G.H.D.L. and H.W.L. analyzed data, performed visualization, participated in result interpretation, and drafted the manuscript. J.R. and T.S. provided technical support. S.L. reviewed the manuscript. X.L. performed the statistical analysis and reviewed the manuscript. B.H.M.L., C.W.W., and I.V.O. reviewed the manuscript. A.A. and F.R. designed the study and provided resources. A.J.R., N.A., E.I., and E.F.F. provided scientific advice and reviewed the manuscript. A.Z. provided conceptualization, reviewed the manuscript, provided resources, and supervised the projects. All authors read and approved the final manuscript.

## FUNDING INFORMATION

This study received no specific grant from any funding agency in the public, commercial, or not‐for‐profit sectors. E.F.F. is supported by Cure Alzheimer's Fund (#282952), HELSE SØR‐ØST (#2020001, #2021021, #2023093), the Research Council of Norway (#262175, #334361), Molecule AG/VITADAO (#282942), NordForsk Foundation (#119986), the National Natural Science Foundation of China (#81971327), Akershus University Hospital (#269901, #261973, #262960), the Civitan Norges Forskningsfond for Alzheimers sykdom (#281931), the Czech Republic‐Norway KAPPA program (with Martin Vyhnálek, #TO01000215), and the Rosa sløyfe/Norwegian Cancer Society & Norwegian Breast Cancer Society (#207819). S.L has received funding from the European Union's Horizon 2020 research and innovation program under the Marie Sklodowska‐Curie grant agreement No 801133.

## CONFLICT OF INTEREST STATEMENT

The authors declare the following competing interests: F.W.P., G.H.D.L., H.W.L., X.L., B.H.M.L., C.W.W., A.A., and A.Z. are affiliated with Insilico Medicine, a commercial company developing AI solutions for aging research, drug discovery, and longevity medicine. E.F.F. has an MTA with LMITO Therapeutics Inc (South Korea), a CRADA arrangement with ChromaDex (USA), and a commercialization agreement with Molecule AG/VITADAO, and is a consultant to Aladdin Healthcare Technologies (UK and Germany), the Vancouver Dementia Prevention Centre (Canada), Intellectual Labs (Norway), MindRank AI (China), and NYo3 (China).

## Supporting information


Figure S1.

Figure S2.

Figure S3.

Figure S4.

Figure S5.

Tables S1–S9.

## Data Availability

PandaOmics is commercially available at https://pandaomics.com/. RNA‐sequencing data used for performing age‐associated expression analyses are publicly available on the Genotype‐Tissue Expression (GTEx) website (available at https://gtexportal.org/home/). Samples' metadata including the donor's age and sex were downloaded after the access to GTEx V9‐protected data (phs000424.v9.p2) was authorized by the Data Access Committee of NCBI dbGAP. Analyses for the 11 selected solid cancers were performed using 139 publicly available datasets. Transcriptomics datasets were collected from GEO, ArrayExpress, and TCGA databases. Proteomics datasets were collected from PRIDE and PDC databases. All dataset identifiers are included in Table S1.
