# Supplementary figures and images for: A comprehensive AI‐driven analysis of large‐scale omic datasets reveals novel dual‐purpose targets for the treatment of cancer and aging
Source: Aging Cell. 2023 Oct 27;22(12):e14017. doi: 10.1111/acel.14017 (PMC10726874; doi:10.1111/acel.14017)

Downregulated age-associated TSGs

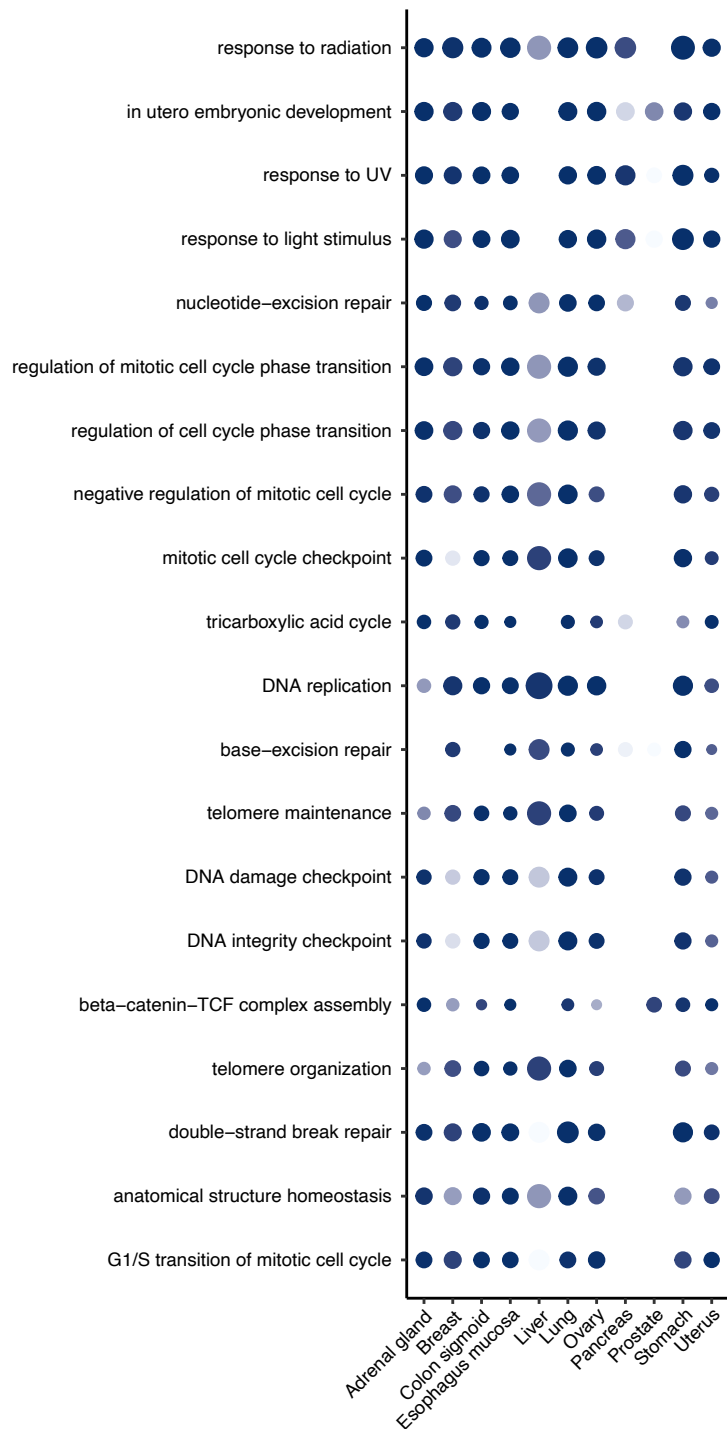

FDR 0.04 0.03 0.02 0.01

Upregulated age-associated TSGs

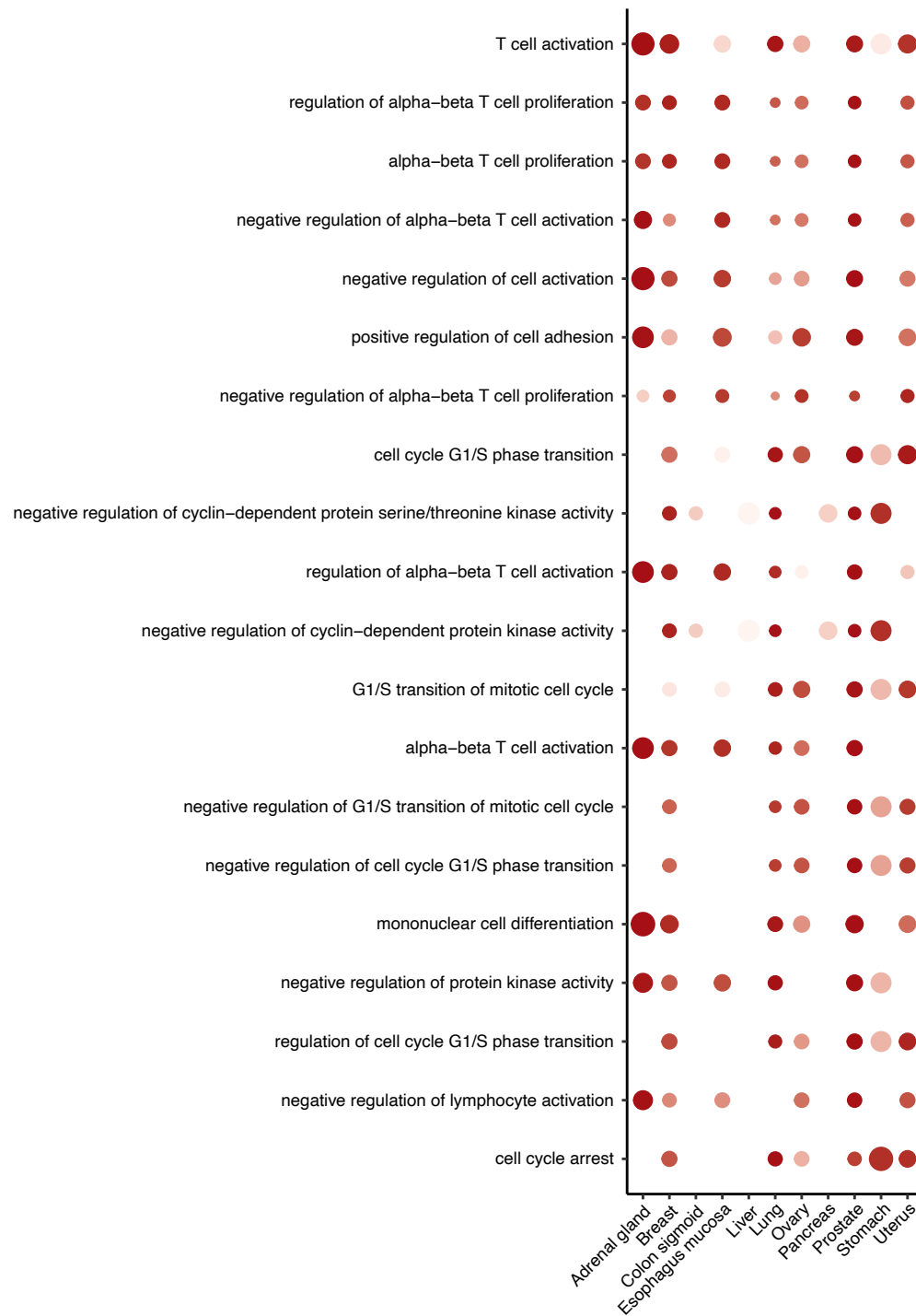

FDR 0.04 0.03 0.02 0.01

GeneRatio 0.1 0.2 0.3 0.4 0.5

Supplement: Supplementary file 1 — Figure S1. [file ACEL-22-e14017-s005.pdf]

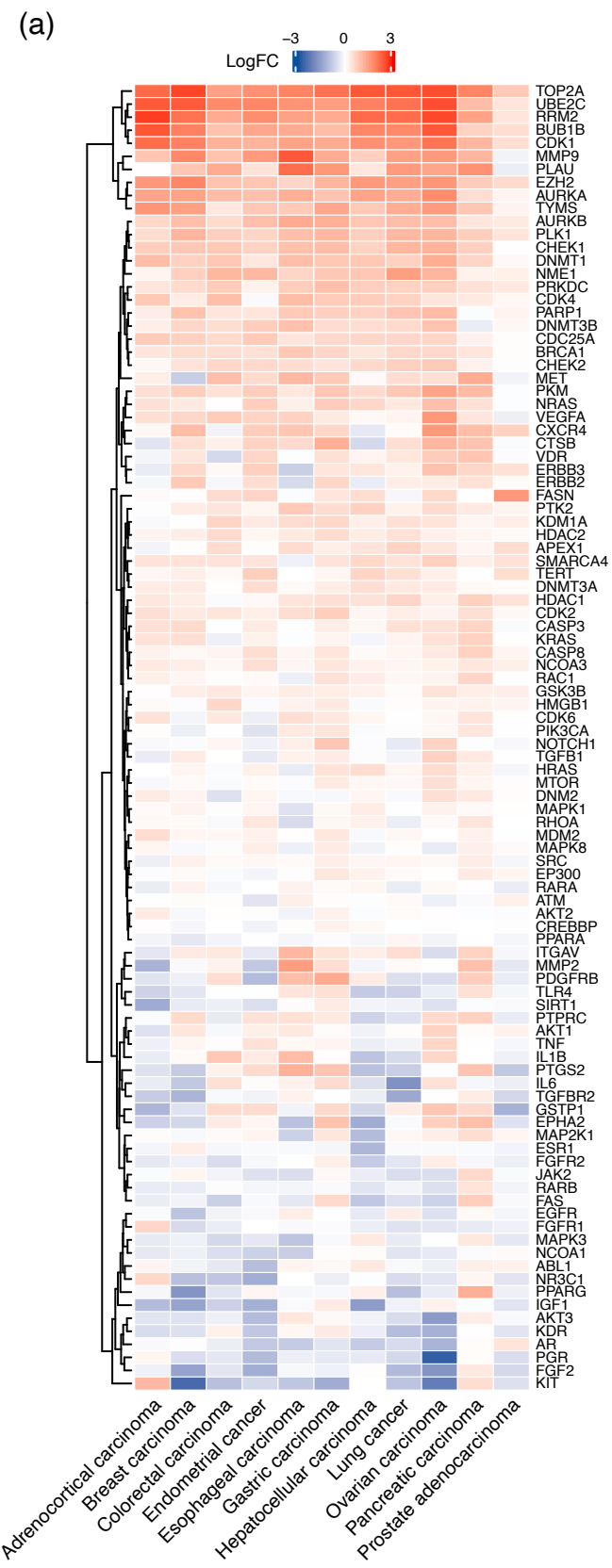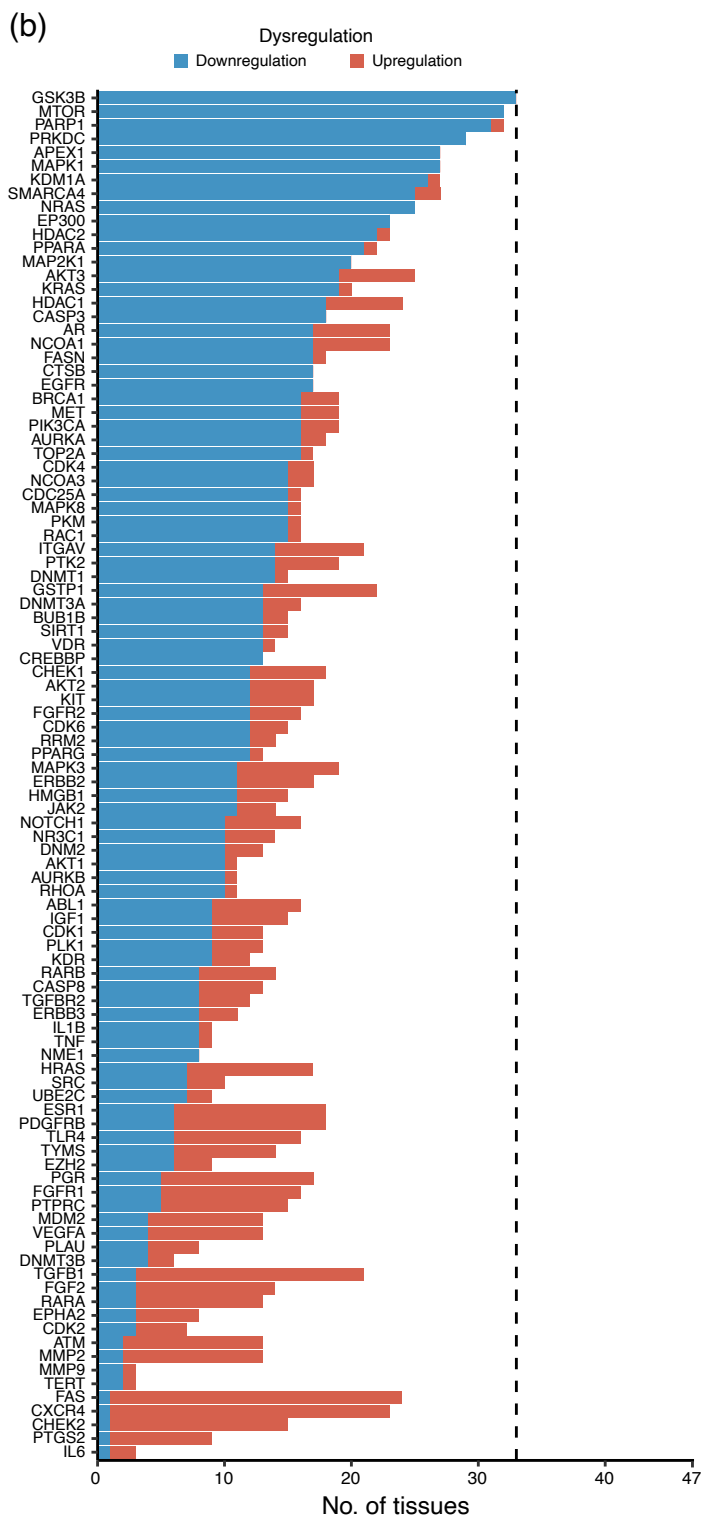

Supplement: Supplementary file 2 — Figure S2. [file ACEL-22-e14017-s001.pdf]

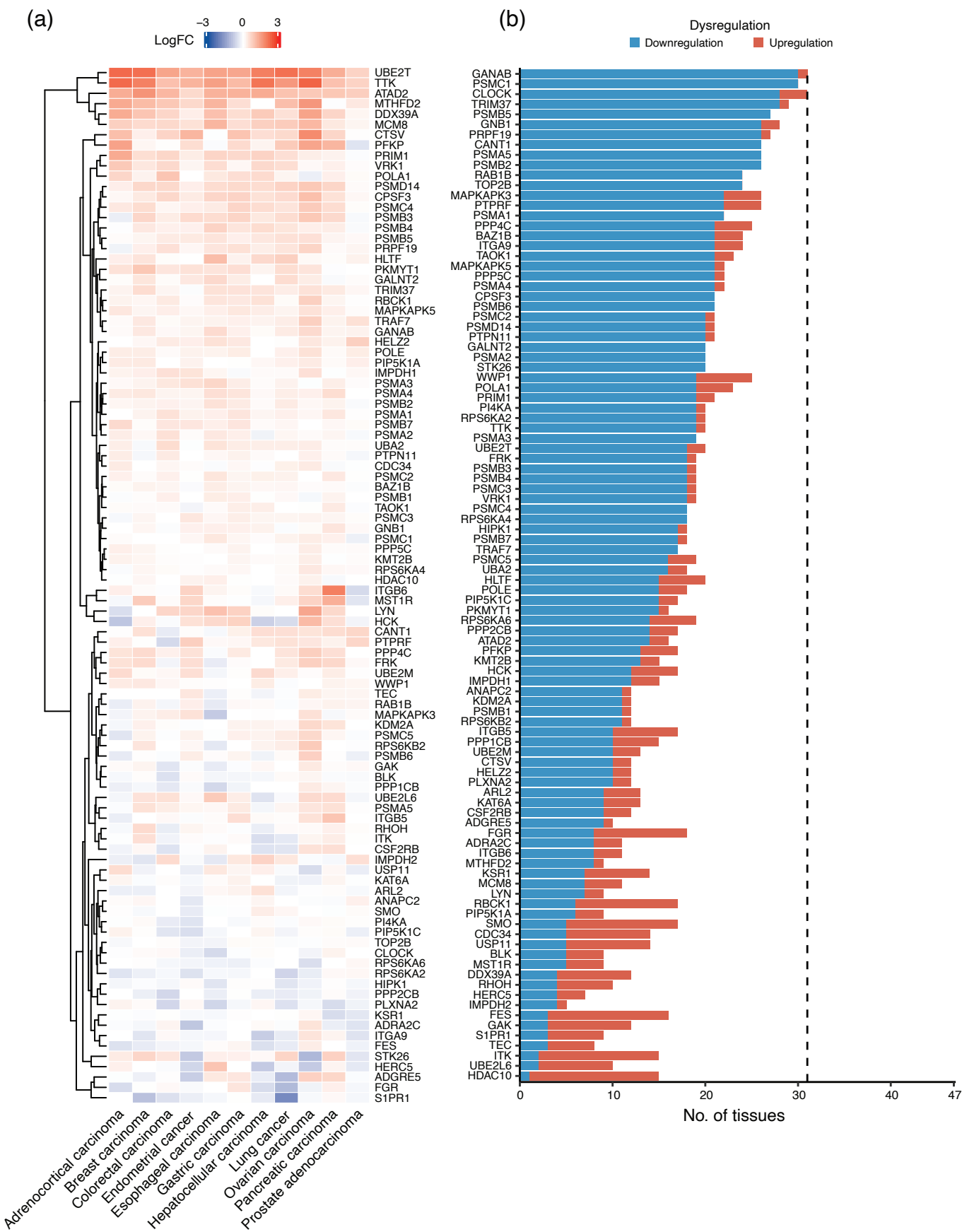

Supplement: Supplementary file 3 — Figure S3. [file ACEL-22-e14017-s004.pdf]

Fold Change compared to L4440

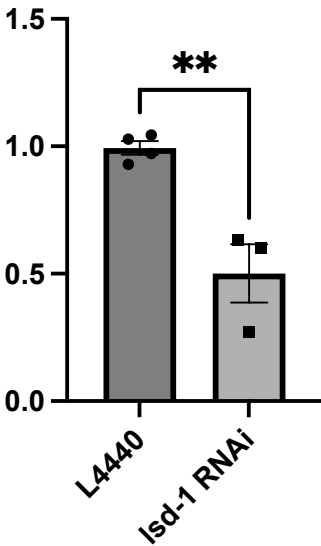

Supplement: Supplementary file 5 — Figure S5. [file ACEL-22-e14017-s003.pdf]
