# Supplementary material for: A comprehensive AI‐driven analysis of large‐scale omic datasets reveals novel dual‐purpose targets for the treatment of cancer and aging
Source: Aging Cell. 2023 Oct 27;22(12):e14017. doi: 10.1111/acel.14017 (PMC10726874; doi:10.1111/acel.14017)
Supplement: Supplementary file 4 — Figure S4. [file ACEL-22-e14017-s006.pdf]

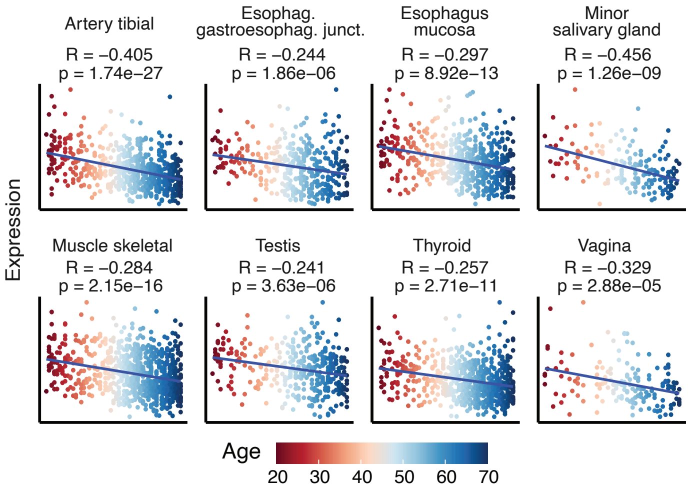

Artery tibial
R = −0.405
p = 1.74e−27
Esophag. gastroesophag. junct.
R = −0.244
p = 1.86e−06
Esophagus mucosa
R = −0.297
p = 8.92e−13
Minor salivary gland
R = −0.456
p = 1.26e−09
Muscle skeletal
R = −0.284
p = 2.15e−16
Testis
R = −0.241
p = 3.63e−06
Thyroid
R = −0.257
p = 2.71e−11
Vagina
R = −0.329
p = 2.88e−05
Expression
Age
20 30 40 50 60 70
