# Supplementary material for: A comprehensive AI‐driven analysis of large‐scale omic datasets reveals novel dual‐purpose targets for the treatment of cancer and aging
Source: Aging Cell. 2023 Oct 27;22(12):e14017. doi: 10.1111/acel.14017 (PMC10726874; doi:10.1111/acel.14017)
Supplement: Supplementary file 6 — Tables S1–S9. [file ACEL-22-e14017-s002.pdf]

Table S1. Disease and dataset selection

Solid cancers (n = 11)	Number of dataset comparisons	Number of cases	Number of controls	Dataset identifiers
Adrenal cortex carcinoma	6	198	42	GSE10927, GSE12368, GSE19750, GSE33371, GSE75415, GSE90713
Breast carcinoma	19	2,424	739	GSE10780, GSE115144, GSE120129, GSE17907, GSE31448, GSE36295 GSE38959, GSE57297, GSE5764, GSE58135, GSE59246, GSE61304, GSE61723, GSE65212, GSE70905, GSE70947, GSE71651, GSE83591, TCGA-BRCA
Colorectal carcinoma	19	1,211	523	E-MTAB-57, GSE123390, GSE18105, GSE20842, GSE21510, GSE22598 GSE28866, GSE32323, GSE41657, GSE4183, GSE44076, GSE62932 GSE68468, GSE75548, GSE76855, GSE77199, GSE89076, PDC000116 TCGA-COADREAD
Endometrial cancer	7	786	102	GSE115810, GSE17025, GSE36389, GSE56087, GSE63678, PDC000125 TCGA-UCEC
Esophageal carcinoma	8	473	188	GSE1420, GSE161533, GSE20347, GSE23400, GSE70409, GSE74553 GSE75241, TCGA-ESCA
Gastric carcinoma	20	1,111	698	E-MTAB-1440, GSE103236, GSE118897, GSE122401, GSE13195, GSE13911 GSE19826, GSE27342, GSE29272, GSE29998, GSE30727, GSE33335 GSE37023, GSE37023, GSE51575, GSE56807, GSE63089, GSE79973 GSE85465, TCGA-STAD
Hepatocellular carcinoma	13	1,118	651	E-MTAB-5905, GSE102079, GSE107170, GSE19665, GSE36376, GSE45267 GSE59259, GSE60502, GSE6764, GSE67764, GSE82177, PDC000198 TCGA-LIHC
Lung cancer	23	2,457	1,012	E-MEXP-231, E-MTAB-1690, E-MTAB-3950, E-MTAB-5231, GSE104854 GSE18842, GSE19188, GSE19804, GSE28866, GSE30219, GSE40791 GSE43458, GSE44077, GSE63459, GSE74706, GSE75324, GSE83227 GSE84776, PDC000153, PDC000219, PDC000234, TCGA-LUAD, TCGA-LUSC
Ovarian carcinoma	5	112	28	GSE10971, GSE124766, GSE146553, GSE27651, GSE36668
Pancreatic carcinoma	15	595	301	GSE136569, GSE141873, GSE143754, GSE15471, GSE16515, GSE18670 GSE22780, GSE28735, GSE55643, GSE56560, GSE60646, GSE62165 GSE62452, GSE63111, PDC000270
Prostate adenocarcinoma	6	818	147	E-MTAB-6128, GSE111320, GSE68555, GSE6956, PXD010744, TCGA-PRAD

TOTAL	141	11,303	4,431

GTEx healthy tissue (n = 47	Number of all samples	Number of young gr	Number of old group (aged 60-79)
Adipose subcutaneous	663	213	237
Adipose visceral omentum	541	175	182
Adrenal gland*	258	88	81
Artery aorta	432	143	140
Artery coronary	240	63	82
Artery tibial	663	232	215
Brain amygdala	152	22	85
Brain anterior cingulate corte	176	32	103
Brain caudate basal ganglia	246	38	135
Brain cerebellar hemisphere	215	31	119
Brain cerebellum	241	39	127
Brain cortex	255	42	136
Brain frontal cortex ba9	209	26	120
Brain hippocampus	197	31	114
Brain hypothalamus	202	25	117
Brain nucleus accumbens bas	246	36	135
Brain putamen basal ganglia	205	29	109
Brain spinal cord cervical c-1	159	22	90
Brain substantia nigra	139	23	81
Breast mammary tissue*	459	165	155
Cells ebv-Transformed lympl	174	70	50
Colon sigmoid*^	373	129	135
Colon transverse	406	165	105
Esophagus gastroesophageal	375	135	103
Esophagus mucosa*^	555	208	166
Esophagus muscularis	515	206	143
Heart atrial appendage	429	95	180
Heart left ventricle	432	114	164
Liver*	226	59	84
Lung*	578	166	212
Minor salivary gland	162	60	52
Muscle skeletal	803	256	292
Nerve tibial	619	200	229
Ovary*	180	73	46
Pancreas*	328	126	84

Pituitary	283	41	155
Prostate*	245	93	80
Skin not sun exposed suprapu	604	188	226
Skin sun exposed lower leg	701	223	260
Small intestine terminal ileum	187	85	46
Spleen	241	101	54
Stomach*	359	147	84
Testis	361	119	125
Thyroid	653	208	234
Uterus*	142	69	28
Vagina	156	64	42
Whole blood	755	249	272
TOTAL	16,740	5,124	6,214

* Tissues corrsponding to the 11 studied cancers

^ Colon sigmoid and esophagus mucosa were selected as they are the prevalent primary sites for the corresponding cancers

Table S2. Age-associated expression changes in protein-coding genes and cancer-related genes in 47 GTEx tissues

GTEx healthy tissues[1]	Protein-coding genes[2]				Tumor suppressor genes[3]			
	Number of genes sequenced	Number of dysregulation	Number of upregulation	Number of downregulation	Number of genes sequenced	Number of dysregulation	Numebr of upregulation	Numebr of downregulation
Adrenal gland*	15,690	4,406 (28.08%)	948	3,458	247	85 (34.41%)	17	68
Breast mammary tissue*	15,939	4,321 (27.11%)	1,525	2,796	245	65 (26.53%)	34	31
Colon sigmoid*^	15,865	6,185 (38.99%)	2,453	3,732	250	112 (44.8%)	24	88
Esophagus mucosa*^	15,796	9,436 (59.74%)	2,500	6,936	246	168 (68.29%)	24	144
Liver*	15,483	1,482 (9.57%)	503	979	247	12 (4.86%)	3	9
Lung*	16,104	8,495 (52.75%)	4,009	4,486	250	143 (57.2%)	82	61
Ovary*	15,757	4,519 (28.68%)	2,163	2,356	246	82 (33.33%)	25	57
Pancreas*	15,702	1,770 (11.27%)	339	1,431	248	34 (13.71%)	5	29
Prostate*	16,203	5,048 (31.15%)	2,713	2,335	251	74 (29.48%)	48	26
Stomach*	15,860	2,393 (15.09%)	376	2,017	250	45 (18%)	6	39
Uterus*	15,766	6,944 (44.04%)	1,777	5,167	244	116 (47.54%)	26	90
Adipose subcutaneous	15,697	8,598 (54.77%)	1,952	6,646	245	145 (59.18%)	29	116
Adipose visceral omentum	15,786	8,470 (53.66%)	3,227	5,243	245	141 (57.55%)	45	96
Artery aorta	15,547	8,177 (52.6%)	1,875	6,302	246	152 (61.79%)	27	125
Artery coronary	15,703	2,309 (14.7%)	391	1,918	247	41 (16.6%)	7	34
Artery tibial	15,345	11,245 (73.28%)	1,662	9,583	245	202 (82.45%)	25	177
Brain amygdala	15,953	5,489 (34.41%)	1,368	4,121	248	88 (35.48%)	35	53
Brain anterior cingulate cortex ba24	16,068	3,598 (22.39%)	682	2,916	248	58 (23.39%)	15	43
Brain caudate basal ganglia	16,068	3,093 (19.25%)	697	2,396	249	42 (16.87%)	7	35
Brain cerebellar hemisphere	15,896	2,652 (16.68%)	746	1,906	249	42 (16.87%)	12	30
Brain cerebellum	16,008	3,240 (20.24%)	1,026	2,214	251	51 (20.32%)	16	35
Brain cortex	16,150	4,672 (28.93%)	1,501	3,171	249	68 (27.31%)	21	47
Brain frontal cortex ba9	16,070	5,742 (35.73%)	1,941	3,801	249	93 (37.35%)	33	60
Brain hippocampus	15,996	4,841 (30.26%)	1,082	3,759	248	71 (28.63%)	23	48
Brain hypothalamus	16,194	4,356 (26.9%)	822	3,534	249	76 (30.52%)	15	61
Brain nucleus accumbens basal ganglia	16,071	2,234 (13.9%)	838	1,396	249	37 (14.86%)	7	30
Brain putamen basal ganglia	15,930	1,371 (8.61%)	396	975	248	12 (4.84%)	2	10
Brain spinal cord cervical c-1	15,924	416 (2.61%)	278	138	248	9 (3.63%)	7	2
Brain substantia nigra	15,905	1040 (6.54%)	288	752	248	16 (6.45%)	4	12
Cells ebv-Transformed lymphocytes	15,037	1092 (7.26%)	967	125	244	13 (5.33%)	13	0
Colon transverse	16,099	10,040 (62.36%)	6,254	3,786	251	141 (56.18%)	77	64
Esophagus gastroesophageal junction	15,695	6,817 (43.43%)	1,377	5,440	249	127 (51%)	19	108
Esophagus muscularis	15,686	8,008 (51.05%)	1,539	6,469	249	143 (57.43%)	18	125
Heart atrial appendage	15,576	4,561 (29.28%)	1,257	3,304	247	76 (30.77%)	22	54
Heart left ventricle	15,194	6,920 (45.54%)	1,811	5,109	244	113 (46.31%)	25	88
Minor salivary gland	16,094	4,925 (30.6%)	1,198	3,727	247	77 (31.17%)	8	69
Muscle skeletal	15,090	9,728 (64.47%)	1,923	7,805	241	169 (70.12%)	31	138
Nerve tibial	15,917	8,537 (53.63%)	2,253	6,284	246	129 (52.44%)	42	87
Pituitary	16,376	1,408 (8.6%)	827	581	248	18 (7.26%)	15	3
Skin not sun exposed suprapubic	16,067	6,100 (37.97%)	3,235	2,865	247	102 (41.3%)	71	31
Skin sun exposed lower leg	16,085	6,269 (38.97%)	1,706	4,563	248	111 (44.76%)	34	77

Small intestine terminal ileum	16,179	1,852 (11.45%)	1,226	626	250	35 (14%)	15	20
Spleen	15,873	2,514 (15.84%)	1,226	1,288	245	40 (16.33%)	18	22
Testis	17,631	3,075 (17.44%)	995	2,080	252	54 (21.43%)	9	45
Thyroid	15,955	6,759 (42.36%)	1,689	5,070	245	127 (51.84%)	22	105
Vagina	15,955	5,724 (35.88%)	1,400	4,324	249	98 (39.36%)	9	89
Whole blood	14,375	9,464 (65.84%)	3,955	5,509	239	161 (67.36%)	82	79

[1] Tissues corrsponding to the 11 studied cancers were marked with an asterisk. ^ Colon sigmoid and esophagus mucosa were selected as they are the prevalent primary sites for the corresponding cancers

[2] A total of 19,334 protein coding genes

[3] A total of 252 tumor suppressor genes

Table S3. The list of tumor suppressor genes analyzed

ABI1
ACVR1B
ACVR2A
AMER1
APC
ARHGAP26
ARHGEF10
ARHGEF10L
ARHGEF12
ARID1A
ARID1B
ARID2
ASXL1
ASXL2
ATM
ATP2B3
ATR
ATRX
AXIN1
AXIN2
B2M
BAP1
BARD1
BAX
BAZ1A
BCL10
BCOR
BLM
BRCA1
BRCA2

BRIP1
BTG1
BUB1B
CAMTA1
CASP3
CASP8
CASP9
CBFA2T3
CBFB
CBLB
CCDC6
CCNB1IP1
CCNC
CD274
CDC73
CDH1
CDH10
CDH11
CDK12
CDKN1B
CDKN2A
CDKN2C
CDX2
CEBPA
CHD2
CHEK2
CIC
CIITA
CLTC
CLTCL1
CNBP

CNOT3
CPEB3
CREB3L1
CREBBP
CTCF
CUL3
CYLD
DAXX
DDX10
DDX3X
DICER1
DNM2
DROSHA
EBF1
EED
EIF3E
ELF3
ELL
EP300
EPS15
ERCC2
ERCC3
ERCC4
ERCC5
ETNK1
ETV6
EXT1
EXT2
FANCA
FANCC
FANCD2

FANCE
FANCF
FANCG
FAS
FAT1
FAT4
FBLN2
FBXO11
FBXW7
FEN1
FH
FHIT
FLCN
FUBP1
FUS
GATA1
GATA3
GPC5
GRIN2A
HNF1A
ID3
IGF2BP2
IKZF1
KAT6B
KDM5C
KDM6A
KEAP1
KLF6
KMT2C
KMT2D
KNL1

LARP4B
LATS1
LATS2
LEPROTL1
LRIG3
LRP1B
LZTR1
MAP3K1
MAX
MEN1
MGMT
MLF1
MLH1
MSH2
MSH6
MUTYH
MYH9
N4BP2
NAB2
NBN
NCOA4
NCOR1
NCOR2
NDRG1
NF1
NF2
NFKBIE
NOTCH1
NOTCH2
NRG1
NTHL1

PALB2
PATZ1
PAX5
PBRM1
PER1
PHF6
PHOX2B
PIK3R1
PML
PMS2
POLD1
POLE
POLG
POT1
PPARG
PPP6C
PRDM1
PRDM2
PRF1
PTCH1
PTEN
PTPN13
PTPN6
PTPRB
PTPRC
PTPRD
PTPRK
PTPRT
RAD17
RAD51B
RANBP2

RB1
RBM10
RFWD3
RHOH
RMI2
RNF43
ROBO2
RPL10
RPL22
RPL5
RSPO2
RUNX1
SBDS
SDHA
SDHAF2
SDHB
SDHC
SDHD
SETD1B
SETD2
SFPQ
SFRP4
SH2B3
SIRPA
SLC34A2
SMAD2
SMAD3
SMAD4
SMARCA4
SMARCB1
SMARCD1

SMARCE1
SMC1A
SOCS1
SOX21
SOX9
SPEN
STAG1
STAG2
STK11
SUFU
TET2
TGFBR2
TMEM127
TNFAIP3
TNFRSF14
TP53
TPM3
TRAF7
TRIM33
TSC1
TSC2
USP44
VHL
WIF1
WNK2
WRN
WT1
XPA
XPC
YWHAE
ZBTB16

ZFHX3
ZMYM3
ZNF331
ZNRF3
ZRSR2

Table S4. Overlapped pathways dysregulation in aging and cancer

Cellular processes	The percentage of dysregulated cellular processes[1]											
	Adrenal gland	Breast	Colon sigmoid	Uterus	Esophagus	Stomach	Liver	Lung	Ovary	Pancreas	Prostate	Average
Upregulation in aging & cancer	2.5	11.5	5.1	1.7	6.6	4.1	0.4	11.3	3.3	0.9	5.4	4.8
Downregulation in aging & cancer	12.6	10.9	21.6	17.3	26.2	0.5	9.6	9.9	12	0.9	10.3	12
Upregulation in cancer & downregulation in aging	9.2	8.1	21.7	16.7	41.3	23.6	6.2	24.1	41.6	4.5	7	18.5
Dowregulation in cancer & upregulation in aging	3.3	13.7	11.9	1	6.5	0.7	1.7	30.8	4.1	0.2	21.7	8.7
TOTAL	27.6	44.2	60.3	36.7	80.6	28.9	17.9	76.1	61	6.5	44.4	44

[1] The percentage of dysregulated pathways in all cellular process overlapped in aging and cancer in corresponding tissues.

Table S5. Overlapped unidirectionally dysregulated pathways in aging and cancer

Pathways[1]	Upstream signaling pathways	Main cellular processes	Unidirectional dysregulation in aging and cancer	GTEx tissues	iPANDA score (aging)	Cancer types	iPANDA score (cancer)
Cellular Senescence	Cellular Senescence	Cellular response to external stimuli	Upregulation	Adrenal gland	0.0102	Adrenocortical carcinoma	0.042
Cellular Senescence	Cellular Senescence	Cellular response to external stimuli	Upregulation	Breast	0.0527	Breast carcinoma	0.1831
Cellular Senescence	Cellular Senescence	Cellular response to external stimuli	Upregulation	Lung	0.0313	Lung cancer	0.139
Cellular Senescence	Cellular Senescence	Cellular response to external stimuli	Upregulation	Pancreas	0.0174	Pancreatic carcinoma	0.1421
Cellular Senescence	Cellular Senescence	Cellular response to external stimuli	Upregulation	Prostate	0.0347	Prostate adenocarcinoma	0.077
Cellular Senescence	Cellular Senescence	Cellular response to external stimuli	Upregulation	Stomach	0.0191	Gastric carcinoma	0.1638
Cellular Senescence	Cellular Senescence	Cellular response to external stimuli	Upregulation	Colon sigmoid	0.0112	Colorectal carcinoma	0.0715
Oncogene Induced Senescence	Cellular Senescence	Cellular response to external stimuli	Upregulation	Stomach	0.0506	Gastric carcinoma	0.1495
Oncogene Induced Senescence	Cellular Senescence	Cellular response to external stimuli	Upregulation	Lung	0.0767	Lung cancer	0.0629
Oncogene Induced Senescence	Cellular Senescence	Cellular response to external stimuli	Upregulation	Pancreas	0.051	Pancreatic carcinoma	0.1884
Oncogene Induced Senescence	Cellular Senescence	Cellular response to external stimuli	Upregulation	Prostate	0.0617	Prostate adenocarcinoma	0.0374
Oncogene Induced Senescence	Cellular Senescence	Cellular response to external stimuli	Upregulation	Breast	0.0761	Breast carcinoma	0.1067
Oncogene Induced Senescence	Cellular Senescence	Cellular response to external stimuli	Upregulation	Colon sigmoid	0.0587	Colorectal carcinoma	0.083
Oxidative Stress Induced Senescence	Cellular Senescence	Cellular response to external stimuli	Upregulation	Breast	0.0446	Breast carcinoma	0.2781
Oxidative Stress Induced Senescence	Cellular Senescence	Cellular response to external stimuli	Upregulation	Colon sigmoid	0.0272	Colorectal carcinoma	0.0526
Oxidative Stress Induced Senescence	Cellular Senescence	Cellular response to external stimuli	Upregulation	Lung	0.051	Lung cancer	0.1834
Oxidative Stress Induced Senescence	Cellular Senescence	Cellular response to external stimuli	Upregulation	Pancreas	0.0277	Pancreatic carcinoma	0.1415
Oxidative Stress Induced Senescence	Cellular Senescence	Cellular response to external stimuli	Upregulation	Prostate	0.0419	Prostate adenocarcinoma	0.0938
Oxidative Stress Induced Senescence	Cellular Senescence	Cellular response to external stimuli	Upregulation	Stomach	0.0343	Gastric carcinoma	0.1726
Adenylate cyclase inhibitory pathway	GPCR downstream signalling	Signal transduction	Downregulation	Adrenal gland	-0.0358	Adrenocortical carcinoma	-0.0311
Adenylate cyclase inhibitory pathway	GPCR downstream signalling	Signal transduction	Downregulation	Breast	-0.0313	Breast carcinoma	-0.2577
Adenylate cyclase inhibitory pathway	GPCR downstream signalling	Signal transduction	Downregulation	Colon sigmoid	-0.0108	Colorectal carcinoma	-0.1508
Adenylate cyclase inhibitory pathway	GPCR downstream signalling	Signal transduction	Downregulation	Esophagus	-0.0785	Esophageal carcinoma	-0.0703
Adenylate cyclase inhibitory pathway	GPCR downstream signalling	Signal transduction	Downregulation	Liver	-0.0209	Hepatocellular carcinoma	-0.0156
Adenylate cyclase inhibitory pathway	GPCR downstream signalling	Signal transduction	Downregulation	Ovary	-0.1015	Ovarian carcinoma	-0.0557
Adenylate cyclase inhibitory pathway	GPCR downstream signalling	Signal transduction	Downregulation	Uterus	-0.1387	Endometrial cancer	-0.3542
G-protein mediated events	GPCR downstream signalling	Signal transduction	Downregulation	Adrenal gland	-0.0259	Adrenocortical carcinoma	-0.0384
G-protein mediated events	GPCR downstream signalling	Signal transduction	Downregulation	Breast	-0.0225	Breast carcinoma	-0.1996
G-protein mediated events	GPCR downstream signalling	Signal transduction	Downregulation	Colon sigmoid	-0.0125	Colorectal carcinoma	-0.1505
G-protein mediated events	GPCR downstream signalling	Signal transduction	Downregulation	Esophagus	-0.0907	Esophageal carcinoma	-0.0604
G-protein mediated events	GPCR downstream signalling	Signal transduction	Downregulation	Liver	-0.0145	Hepatocellular carcinoma	-0.0472
G-protein mediated events	GPCR downstream signalling	Signal transduction	Downregulation	Ovary	-0.087	Ovarian carcinoma	-0.056
GAB1 signalosome	Signaling by EGFR	Signal transduction	Downregulation	Colon sigmoid	-0.0399	Colorectal carcinoma	-0.0389
GAB1 signalosome	Signaling by EGFR	Signal transduction	Downregulation	Esophagus	-0.2263	Esophageal carcinoma	-0.2972
GAB1 signalosome	Signaling by EGFR	Signal transduction	Downregulation	Lung	-0.0635	Lung cancer	-0.1342
GAB1 signalosome	Signaling by EGFR	Signal transduction	Downregulation	Ovary	-0.06	Ovarian carcinoma	-0.0135
GAB1 signalosome	Signaling by EGFR	Signal transduction	Downregulation	Prostate	-0.0481	Prostate adenocarcinoma	-0.1793
GAB1 signalosome	Signaling by EGFR	Signal transduction	Downregulation	Uterus	-0.2109	Endometrial cancer	-0.0737
MAP2K and MAPK activation	MAPK1/MAPK3 signaling	Signal transduction	Downregulation	Breast	-0.0132	Breast carcinoma	-0.0102
MAP2K and MAPK activation	MAPK1/MAPK3 signaling	Signal transduction	Downregulation	Colon sigmoid	-0.0405	Colorectal carcinoma	-0.0972
MAP2K and MAPK activation	MAPK1/MAPK3 signaling	Signal transduction	Downregulation	Esophagus	-0.09	Esophageal carcinoma	-0.0466
MAP2K and MAPK activation	MAPK1/MAPK3 signaling	Signal transduction	Downregulation	Liver	-0.0105	Hepatocellular carcinoma	-0.0521
MAP2K and MAPK activation	MAPK1/MAPK3 signaling	Signal transduction	Downregulation	Lung	-0.0386	Lung cancer	-0.1614
MAP2K and MAPK activation	MAPK1/MAPK3 signaling	Signal transduction	Downregulation	Prostate	-0.057	Prostate adenocarcinoma	-0.0723

[1] Reactome pathways that are dysregulated in a unidirectional manner in more than 5 types of cancer (i.e., 50%) and their corresponding healthy GTEx tissues

Table S6. Top 100 AI-derived common high confidence and novel cancer targets

				Ranking					Aging				Cancer								Aging-association			
Targets	Protein family	Clinical trial status	Novelty[1]	Overall rank[2]	Number of cancers (ranked as top 100)	Average rank	Number of aging hallmarks[3]	Hallmarks of aging	Number of dysregulation	Number of upregulation	Number of downregulation	Number of cancer hallmarks[4]	Number of dysregulation	Number of upregulation	Number of downregulation	Therapeutic approach for cancer treatment[6]	Reported in database; Results	Observed effects on longevity[5]	Therapeutic approach for anti-aging	Involved in target wheel[7]	Group 1: Evidence in extending lifespan, dual-purpose (same direction of therepeutic inhbition or activation)<br>Group 2: Evidence in extending lifespan, not dual-purpose (opposite direction of therepeutic inhibition or activation)<br>Group 3: Evidence in shortening lifespan only<br>Group 4: Unexploring role in regulating lifespan<br>NA: Excluded in target wheel (due to lack of aging hallmarks or less than 7 tissues dysregulated GTEx tissues during aging)	Source (DrugAge/ GenAge / Geroprotectors / SynergyAge)	Remarks	References
AKT1	AGC kinase	Yes	HC	1	11	11.5	12	Altered intercellular communications, Cellular senescence, Deregulated nutrient signaling, Epigenetic shift, Extracellular matrix stiffness, Genomic instability, Impaired proteostasis, Inflammation, Mitochondrial dysfunction, Retrotranspositions, Stem [illegible]	11	1	10	10	8	5	3	Antagonism	Reported; Lifespan extension	Anti-Longevity:<br>Deletion mutation (akt-1 mg306) increased lifespan in *C. elegans* .<br>Deletion mutation (akt-1 ok525) increased lifespan in *C. elegans* .<br>Deletion mutation (akt-2 ok393) increased lifespan in *C. elegans*.<br>Heterogeneous knockout mutants (akt1) increased lifespan in mice.	Antagonism	Yes	Group 1	SynergyAge,GenAge	AKT1 as a therapeutic target (antagonist) for cancer.	PMID: 23935948, PMID: 30808672, PMID: 19240135, PMID: 18241854, PMID: 23786484, PMID: 16839187, PMID: 23415228, PMID: 20620993, PMID: 21803287, PMID: 15068796, PMID: 21803287, PMID: 23415228, PMID: 23935948
ESR1	Nuclear receptor	Yes	HC	2	11	22.4	5	Epigenetic shift, Extracellular matrix stiffness, Genomic instability, Inflammation, Stem cell exhaustion	18	12	6	5	10	1	9	Antagonism*	Reported; Lifespan extension	Pro-Longevity:<br>17-a-estradiol (ESR1 agonist) extended lifespan in mice i.e. preferentially in males.<br>Beta-Estradiol (ESR1 agonist) induced the ESR1 translocation to the cell membrane,<br>and was shown to increase lifespan in C.elegans.<br>Estradiol (ESR1 agonist) extended lifespan significantly in C. elegans and mice.	Agonism	Yes	Group 2	rugAge, Geroprotectors	Antagonist or agonist for cancer.<br>Fulvestrant and amoxifen are used for breast cancer treatment. For instance, fulvestrant, ESR1 inhibitor, used to treat HR+ breast cancer that may also be HER2-,<br>while amoxifen is both an antagonist and an agonist of the estrogen receptor.	PMID: 24245565, PMID: 17928626, PMID: 9045319, PMID: 24134630, PMID: 27312235, PMID: 33723245, PMID: 33788371
PLK1	Protein kinase	Yes	HC	3	11	29.5	4	Cellular senescence, Genomic instability, Impaired proteostasis, Inflammation	13	4	9	2	11	11	0	Antagonism	Not reported	No evidence	NA	Yes	Group 4	-	Antagonist for cancer.<br>PLK1 is involved in cell cycle regulation and DNA checkpoint during healthy aging;<br>PLK1 promotes autophagy.<br>polo loss-of-function increased lifespan of alzheimer-like *Drosophila* .	PMID: 28102733, PMID: 26597721
DNMT1	Methyltransferase	Yes	HC	4	11	30.4	2	Epigenetic shift, Genomic instability	15	1	14	1	10	10	0	Antagonism	Not reported	No evidence	NA	Yes	Group 4	-	Antagonist for cancer.<br>DNMT1 maintains genomic methylation stabiluty and insufficient DNA methylation affects healthy aging and promotes age-related health problems<br>No difference in longevity was observed between Dnmt1-deficient mice and normal controls.	PMID: 22704347, PMID: 16510855
CDK1	CMGC kinase	Yes	HC	5	11	31.2	6	Cellular senescence, Extracellular matrix stiffness, Genomic instability, Impaired proteostasis, Mitochondrial dysfunction, Stem cell [illegible]	13	4	9	3	11	11	0	Antagonism	Reported; Lifespan extension	Anti-Longevity:<br>cdk-1 RNAi increased lifespan by 43% in *C. elegans* .	Antagonism	Yes	Group 1	-	Antagonist for cancer.<br>CDK1 is involved in cell cycle regulation during healthy aging	PMID: 27668945
CHEK1	Protein kinase	Yes	HC	6	11	31.6	3	Cellular senescence, Epigenetic shift, Genomic instability	18	6	12	2	10	10	0	Antagonism	Reported; Lifespan extension	Anti-Longevity:<br>chk-1 (RNAi) increased lifespan by 15% to 25% in *C. elegans* .	Agonism	Yes	Group 2	GenAge	Antagonist for cancer.<br>CHEK1 is involved in DNA damage response and cell cycle checkpoint response during healthy aging	PMID: 16741121
EGFR	Receptor kinase	Yes	HC	7	11	35.6	10	Altered intercellular communications, Cellular senescence, Deregulated nutrient signaling, Epigenetic shift, Extracellular matrix stiffness, Genomic instability, Impaired proteostasis, Inflammation, Stem cell exhaustion, Telomere [illegible]	17	0	17	7	8	2	6	Antagonism	Reported; Lifespan extension	Pro-Longevity:<br>let-23 (gain-of-function) mutants lifespan increased by 29%, while<br>let-23 (loss-of-function) mutants lifespan decreased by 19% in *C. elegans* .	Agonism	Yes	Group 2	GenAge	Antagonist for some cancers but EGFR inhibition results in skin aging and age-related decline.<br>EGFR inhibition was shown to molecular alterations in keratinocytes, likely contributing to the observed skin aging.<br>EGFR activation promoting extended healthspan, while EGFR inactivation associated with age-related decline.	PMID: 27165055, PMID: 20497132
BRCA1	Acyltransferase	No	HC	8	11	36.4	6	Altered intercellular communications, Cellular senescence, Epigenetic shift, Genomic instability, Impaired proteostasis, Stem cell exhaustion	19	3	16	7	10	10	0	Antagonism	Reported; Reduced lifespan only	Pro-Longevity:<br>Heterogeneous BRCA1 mutant lifespan was decreased in mice.	NA	Yes	Group 3	GenAge	Tumor suppressor gene	PMID: 17420720
CHEK2	Protein kinase	Yes	HC	9	11	37.9	3	Cellular senescence, Genomic instability, Impaired proteostasis	15	14	1	2	9	9	0	Antagonism	Reported; Lifespan extension	Anti-Longevity: *<br>Lifespan of Brca1Δ11/Δ11Chk2−/− mice longer than Brca1Δ11/Δ11p53+/− in mice.	Antagonism	Yes	Group 1	GenAge	Tumor suppressor gene<br>CHEK2 inhibitors were invesigated in multiple cancers (max p2 completed, NCT03414047)	PMID: 16675955
KDR	Receptor kinase	Yes	HC	10	11	42.7	8	Cellular senescence, Deregulated nutrient signaling, Extracellular matrix stiffness, Genomic instability, Impaired proteostasis, Mitochondrial dysfunction, Stem cell exhaustion, Telomere [illegible]	12	3	9	7	9	2	7	Antagonism*	Not reported	No evidence	NA	Yes	Group 4	-	KDR antagonists such as Ramucirumab, Cabozantinib approved for cancer treatment but our data showed KDR downregulation in cancers.<br>KDR may be needed for normal angiogenesis, to ensure developing or healing tissues receive an adequate supply of nutrients.	
MMP2	Peptidase	Yes	HC	11	11	44.5	6	Extracellular matrix stiffness, Genomic instability, Impaired proteostasis, Inflammation, Mitochondrial [illegible]	13	11	2	5	9	4	5	Antagonism	Not reported	No evidence	NA	Yes	Group 4	-	Matrix metalloproteinases are invovled in cancer invasion and metastasis - advanced stage of cancer.<br>Aging is associated with increased matrix metalloproteinase-2 activity in the human aorta<br>MMP2 was considered as classsical senescence-associated secretory phenotype (SASP).	PMID: 15831360
SMARCA4	Hydrolase	No	HC	12	11	49.7	5	Altered intercellular communications, Cellular senescence, Epigenetic shift, Genomic instability, Stem cell exhaustion	27	2	25	0	11	10	1	Antagonism	Not reported	No evidence	NA	Yes	Group 4	-	Tumor suppressor gene	
CDK4	CMGC kinase	Yes	HC	13	10	16.5	4	Altered intercellular communications, Deregulated nutrient signaling, Epigenetic shift, Extracellular matrix [illegible]	17	2	15	6	10	10	0	Antagonism	Reported; Lifespan extension	Pro-Longevity:<br>Overexpresision of CycD/Cdk4 in *Drosophila* increased lifespan.	Agonism	Yes	Group 2	-	Antagonist for cancer.<br>CDK4 is involved in cell cycle regulation during healthy aging	PMID: 26219626
CDK2	CMGC kinase	Yes	HC	14	10	22.9	2	Cellular senescence, Genomic instability	7	4	3	4	10	10	0	Antagonism	Reported; Lifespan extension	Anti-Longevity:<br>cdk-2 RNAi increased lifespan by 28% in *C. elegans* .	Antagonism	Yes	Group 1	-		PMID: 27668945
PARP1	Glycosyltransferase	Yes	HC	15	10	24.6	10	Altered intercellular communications, Cellular senescence, Deregulated nutrient signaling, Epigenetic shift, Extracellular matrix stiffness, Genomic instability, Inflammation, Mitochondrial dysfunction, Stem cell exhaustion, Telomere [illegible]	32	1	31	3	11	10	1	Antagonism	Reported; Lifespan extension	Anti-Longevity:<br>Overexpression hPARP1 (double knock-in) significantly decreased lifespan in mice.<br>Mutation, RNAi and PARP inhibitors (AZD2281/ABT-888) extended lifespan by 29%, 20%, 15-23% in C. elegans, respectively.<br>pme-1 mutated increased lifespan in *C. elegans* .<br>Muscle PARP1 inhibition extends lifespan in Drosophila	Antagonism	Yes	Group 1	GenAge	Antagonist for cancer.<br>PARP1 is involved in base excision repair during healthy aging.	PMID: 20561897, PMID: 23870130, PMID: 31878234, PMID: 36947517
AURKA	Protein kinase	Yes	HC	16	10	27.0	6	Cellular senescence, Genomic instability, Impaired proteostasis, Inflammation, Mitochondrial dysfunction, Stem cell [illegible]	18	2	16	0	11	11	0	Antagonism	Not reported	No evidence	NA	Yes	Group 4	-	Antagonist for cancer.<br>AURKA is involved in cell cycle regulation during healthy aging	

VEGFA	Growth factor	Yes	HC	17	10	29.3	8	Altered intercellular communications, Cellular senescence, Deregulated nutrient signaling, Epigenetic shift, Extracellular matrix stiffness, Genomic instability, Inflammation, Telomere attrition	13	9	4	5	11	10	1	Antagonism	Not reported	No evidence	NA	Yes	Group 4	-	Antagonist for cancer.<br>Counteracting age-related VEGF signaling insufficiency promotes healthy aging and extends lifespan.	PMID:34326210
PPARG	Nuclear receptor	Yes	HC	18	10	30.1	9	Altered intercellular communications, Deregulated nutrient signaling, Epigenetic shift, Extracellular matrix stiffness, Genomic instability, Inflammation, Mitochondrial dysfunction, Stem cell exhaustion, Telomere [illegible]	13	1	12	3	8	2	6	Agonism	Reported; Lifespan extension	Pro-Longevity:<br>Ala/Ala Knock-in lifespan increased by 13%, and hypomorphic mutants decrease lifespan by 11% in mice<br>Bezafibrate agonising PPARG extended lifespan significantly in *C. elegans* .<br>Hypomorphic Pparg1/2 knockout decreased lifespan in mice.	Agonism	Yes	Group 1	ge,GenAge,Geroprotec	Tumor suppressor gene;<br>Agonist for cancer	PMID: 19117549,<br>PMID: 23603800,<br>PMID: 19997628
GSK3B	CMGC kinase	Yes	HC	19	10	30.9	9	Altered intercellular communications, Cellular senescence, Deregulated nutrient signaling, Epigenetic shift, Genomic instability, Impaired proteostasis, Inflammation, Mitochondrial dysfunction, Stem cell exhaustion	33	0	33	7	9	9	0	Antagonism	Reported; Lifespan extension	Anti-Longevity:.<br>sgg (GSK3B) RNAi increased the lifespan in *Drosophila* .<br>Lithium chloride antagonising GSK3B extended lifespan significantly in C. elegans and Drosophila.<br>Worthy note that complete absence of GSK3B in *C. elegans* , *Drosophila* , and mice shortened lifepsn or prevents development.	Antagonism	Yes	Group 1	DrugAge,Geroprotector	Adult overexpression of GSK3B in the brain of mice resulted in neurodegeneration.<br>Key mediators of senescence signaling such as p16 and p53 physically interact with GSK3β, and act as its putative substrates.<br>Low dose lithium uptake promoted longevity in human and *C. elegans* .<br>Lithium chloride antagonising GSK3B extended lifespan significantly in *C. elegans* and *Drosophila* .<br>Therapeutic targeting of GSK3β by microdose lithium later in life decreased senescence signaling and delayed kidney aging in mice.<br>Lithium extended lifespan in female and male in *Drosophila* , when administrated throughout adulthood or only later in life.<br>Importantly, although lithium chloride extended lifespan in *C. elegans* , it could not entend lifespan in null gsk-3 (nr2047) mutant (reduction in lifepsan instead) compared to wild type *C. elegans* . The [illegible]	PMID: 27068460,<br>PMID: 17959600,<br>PMID: 21301855,<br>PMID: 24398558,<br>PMID: 11226152,<br>PMID: 35166234
MTOR	Protein kinase	Yes	HC	20	10	33.4	12	Altered intercellular communications, Cellular senescence, Deregulated nutrient signaling, Epigenetic shift, Extracellular matrix stiffness, Genomic instability, Impaired proteostasis, Inflammation, Mitochondrial dysfunction, Retrotranspositions, Stem [illegible]	32	0	32	6	8	6	2	Antagonism	Reported; Lifespan extension	Anti-Longevity:<br>In roundworms, TOR deficiency more than doubled the lifespan.<br>TOR disruption in *Drosophila* also extended lifespan.<br>Hypomorphic mutation increase lifespan by 20% in mice.<br>Knockdown of TOR increased the longevity of ife-2 mutants in *C. elegans* .<br>MTOR inhibitor, rapamycin extends lifespan and delays cancer in mice.<br>Rapamycin inhibiting MTOR extended longevity in drosophila and mice.	Antagonism	Yes	Group 1	, Geroprotector, Synerg	MTOR inhibitor, everolimus (gastrointestinal or lung origin with unresectable, locally advanced or metastatic disease), temsirolimus (Renal cell carcinoma) were approved drug for cancer treatment.	PMID: 17277769,<br>PMID: 14668850,<br>PMID: 15186745,<br>PMID: 24556924,<br>PMID: 23994476,<br>PMID: 20017609,<br>PMID: 24341993,<br>PMID: 19587680,<br>PMID: 24409289
PIK3CA	Non-protein kinase	Yes	HC	21	10	33.6	9	Altered intercellular communications, Cellular senescence, Deregulated nutrient signaling, Epigenetic shift, Extracellular matrix stiffness, Genomic instability, Impaired proteostasis, Mitochondrial dysfunction, Telomere attrition	19	3	16	9	6	3	3	Antagonism	Reported; Lifespan extension	Anti-Longevity:<br>age-1 mutants lifespan increased by 65% in C. elegans.<br>age-1 (hx546) mutants lifespan increased by 40 - 100% in *C. elegans* .<br>age-1(RNAi) increased lifespan by 170% in C. elegans.<br>age-1(mg44) mutants lifespan increased by 1000% in *C. elegans*.<br>age-1(hx546) mutant C. elegans were long-lived by almost two-fold.<br>age-1(mg44) mutant C. elegans extended lifespan compared to wild-type.<br>Pictilisib inhibiting PIK3CA extended lifespan significantly in *C. elegans* .	Antagonism	Yes	Group 1	Age, GenAge, SynergyA	Suppressing the activity of the p110alpha isoform of PIK3CA preserved cardiac function and prevented cardiac aging in mice.<br>MTOR is a downstream effectos of PI3K.<br>Selective anti-cancer agnet wortmannin, PI3K inhibitor, increased lifespan in *Drosophila* .<br>PI3-kinase p110-alpha subunit inhibitor was investigation drugs in various clincial trials for cancer treatment.	PMID: 23543623,<br>PMID: 2392681,<br>PMID: 7789761,<br>PMID: 8608934,<br>PMID: 15998808,<br>PMID: 17996009,<br>PMID: 19822807,<br>PMID: 24096697,<br>PMID: 14668486,<br>PMID: 18828672
ERBB2	Receptor kinase	Yes	HC	22	10	35.5	3	Cellular senescence, Deregulated nutrient signaling, Impaired proteostasis	17	6	11	6	10	6	4	Antagonism	Reported; Lifespan extension	Pro-Longevity:<br>Let-23 mutants (reduction-of-function) survive less robustly in middle adulthood than matched wild type controls (19% decrease of median lifespan, 8% decrease of maximum lifespan) in *C. elegans* .<br>Let-23 mutants (gain-of-function) survive more robustly in middle adulthood (29% increase of median lifespan, 9% increase of maximum lifespan) in *C. elegans* .	Agonism	Yes	Group 2	GenAge	Antagonist for cancer but ERBB2 signaling is important for health.<br>ERBB2 signaling in the heart is essential for the prevention of dilated cardiomyopathy,<br>and ERBB2 is necessart for oligodendrocytes, specialized glial cells, myelinate CNS axons.<br>ERBB2 is healthy driver.	PMID: 35370556,<br>PMID: 14749494,<br>PMID: 20497132,<br>ncg.kcl.ac.uk/index.php
MET	Receptor kinase	Yes	HC	23	10	39.3	3	Impaired proteostasis, Mitochondrial dysfunction, Telomere [illegible]	19	3	16	8	10	8	2	Antagonism	Not reported	No evidence	NA	Yes	Group 4	-	Antagonist for cancer.<br>MET is a target of multiple approved drugs such as cabozantinib, crizotinib and brigatinib for cancer	PMID: 28677234
MMP9	Peptidase	Yes	HC	24	10	39.9	6	Extracellular matrix stiffness, Genomic instability, Impaired proteostasis, Inflammation, Mitochondrial [illegible]	3	1	2	7	9	9	0	Antagonism	Not reported	No evidence	NA	No	-	-	< 7 tissues (out of 47) dysregulated during aging<br>Antagonist for cancer.<br>MMP-9 short interfering RNA induced senescence and MMP9 is one of CellAge Database of Cell Senescence Genes	PMID: 17510426,<br>https://genomics.senescence.info/
IL6	Interleukin	Yes	HC	25	10	53.0	7	Altered intercellular communications, Deregulated nutrient signaling, Epigenetic shift, Extracellular matrix stiffness, Genomic instability, Inflammation, Stem cell exhaustion	3	2	1	6	6	2	4	Antagonism	Not reported	No evidence	NA	No	-	-	< 7 tissues (out of 47) dysregulated during aging<br>IL6 antagonists such as Siltuximab approved for immune system disease and investigatied for cancer but our data showed IL6 downregulation in cancers.<br>Pro-inflammatory cytokine IL-6 is commonly present in the the senescence-associated secretory phenotype (SASP).<br>Interleukin-6 knockout inhibits senescence of bone mesenchymal stem cells in high-fat diet-induced bone loss	PMID: 33679606
CDK6	CMGC kinase	Yes	HC	26	9	27.6	4	Altered intercellular communications, Cellular senescence, Extracellular matrix stiffness, Stem cell exhaustion	15	3	12	4	7	5	2	Antagonism	Not reported	No evidence	NA	Yes	Group 4	-	Antagonist for cancer.<br>CDK6 is involved in cell cycle regulation during healthy aging	
AR	Nuclear receptor	Yes	HC	27	9	31.9	6	Altered intercellular communications, Deregulated nutrient signaling, Epigenetic shift, Extracellular matrix stiffness, Genomic instability, Inflammation	23	6	17	2	8	1	7	nism / Antagon	Reported; Lifespan extension	Pro-Longevity:<br>Danazol agonising AR & PGR extended lifespan in *C. elegans* .	Agonism	Yes	Group 2	DrugAge,Geroprotector	Progressive reduced insulin sensitivity and impaired glucose tolerance were seen in AR-knockout mice with advancing age.<br>Aging AR-knockout mice displayed accelerated weight gain, hyperinsulinemia, and hyperglycemia, and loss of AR contributes to increased triglyceride content in skeletal muscle and liver.<br>Dietary calorie restriction, which retards age-related diseases and extends lifespan, reverses loss of AR expression and restores androgen sensitivity of the aging liver.<br>AR antagonist was approved for the treatment of prostate cancer, while AR agonist was approved for the treatment of breast cancer. Our data showed AR downregulation in 6 cancers.	PMID: 15919793,<br>PMID: 1986927,<br>PMID: 8706808,<br>PMID: 24134630
IGF1	Growth factor	Yes	HC	28	9	32.0	12	Altered intercellular communications, Cellular senescence, Deregulated nutrient signaling, Epigenetic shift, Extracellular matrix stiffness, Genomic instability, Impaired proteostasis, Inflammation, Mitochondrial dysfunction, Retrotranspositions, Stem cell exhaustion, Telomere attrition	15	6	9	8	8	1	7	Antagonism*	Reported; Lifespan extension	Anti-Longevity:<br>Congenital liver IGF-1-deficient mice were increased with lifespan.<br>Hypomorphic mutation increased maximum lifespan by 12% in mice.<br>*C. elegans* lifespan extension can be achieved by reduced Daf-2/insulin/Igf-like signalling<br>Pro-longevity:<br>IGF-1 cardiac-specific overexpression increased lifespan in mice.<br>Overexpression of dilp6 increased lifespan in *Drosophila* .<br>Knockdown of dilp2,3,5 decreased lifespan by 10.5% in male, 18.5% in virgin females and 33.5% in mated females (*Drosophila*).<br>No effects:<br>dilp2 RNAi only was not sufficient to change the lifespan in *Drosophila* .	nism / Antagon	Yes	Group 2	GenAge	IGF1 inhibitors such as xentuzumab, dusigitumab were investigated for cancer treatment in clinical trials.<br>Insulin resistance is increased with aging. Mice lacking IGF1 in the liver have shown to display enhanced insulin sensitivity and glucose homeostasis. Female liver-specific IGF-I inactivation mice showed a 16% increase in lifespan. However, IGF-1 cardiac-specific overexpression increased lifespan in mice . The effects of IGF-1 on longevity may be tissue-specific.	PMID: 21799924,<br>PMID: 24341939,<br>PMID: 23873963,<br>PMID: 17973971,<br>PMID: 15708981,<br>PMID: 22935001,<br>PMID: 19005568,<br>PMID: 20395504

NR3C1	Nuclear receptor	Yes	HC	29	9	34.8	4	Altered intercellular communications, Extracellular matrix stiffness, Genomic instability, Mitochondrial dysfunction	14	4	10	0	10	2	8	Agonism	Not reported	No evidence	NA	Yes	Group 4	-	NR3C1 agonist was investigated for cancer treatment in clinical trials.<br>Mice with impaired NR3C1 function showed behavioural deficits indicative of cognitive impairment.<br>Cyproterone acetate inhibiting AR, PGR & NR3C1 (approved for human use) extended lifespan in *C. elegans* .<br>Dexamethasone, NR3C1 agonist, has been used for cancer treatment.	PMID: 33684964, PMID: 24134630
MAPK3	CMGC kinase	Yes	HC	30	9	35.4	7	Altered intercellular communications, Cellular senescence, Genomic instability, Impaired proteostasis, Mitochondrial dysfunction, Stem cell exhaustion, Telomere	19	8	11	9	10	2	8	Antagonism*	Reported; Reduced lifespan onl	Pro-Longevity:<br>RNA interference of mpk-1 resulted in a decrease in lifespan in *C. elegans* .	NA	Yes	Group 3	GenAge	MAPK3 (ERK1) antagonists such as ulixertinib and temuterkib were investigated for cancer treatment.<br>Age-associated selective impairment in the MAPK signaling pathways in the aged brain.<br>Lifelong caloric restriction completely prevented the age-related decrease in basal brain ERK activity and diminished the age-related reduction of p38 MAPK activity.	PMID: 10647963, PMID: 20624915, PMID: 18059442
CASP3	Peptidase	Yes	HC	31	9	37.7	6	Altered intercellular communications, Cellular senescence, Extracellular matrix stiffness, Genomic instability, Impaired proteostasis, Mitochondrial dysfunction	18	0	18	8	7	7	0	Antagonism	Reported; Lifespan extension	Anti-Longevity: *<br>RNA interference (ced-3) in adulthood resulted in a 19% increase in mean lifespan in *C. elegans* .	Antagonism	Yes	Group 1	GenAge	Tumor suppressor gene	PMID: 17411345
TGFB1	Growth factor	Yes	HC	32	9	39.1	7	Altered intercellular communications, Deregulated nutrient signaling, Epigenetic shift, Extracellular matrix stiffness, Genomic instability, Inflammation, Stem cell exhaustion	21	18	3	7	9	5	4	Antagonism	Reported; Lifespan extension	Anti-Longevity;<br>daw (RNAi) increased lifespan by 35% in *Drosophila* .<br>Muscle specific daw (RNAi) increased lifespan by 11% while fat specific daw (RNAi) decreased lifespan (PMID: 24244197).	Antagonism	Yes	Group 1	GenAge	TGFB1 inhibitor or binding agent was investigated for cancer treatmant in trials.<br>Upregulation of TGF-β ligands contribute to cell degeneration, tissue fibrosis, inflammation,<br>decreased regeneration capacity, and metabolic malfunction.	PMID: 31658594
NOTCH1	Receptor	Yes	HC	33	9	43.7	5	Altered intercellular communications, Extracellular matrix stiffness, Genomic instability, Inflammation, Stem cell exhaustion	16	6	10	6	9	4	5	Antagonism	Reported; Lifespan extension	Anti-Longevity: *<br>glp-1(q158) mutants lifespan increased by 30% in *C. elegans* .<br>glp-1 (RNAi) increased lifespan by 33% in *C. elegans* .<br>A temperature-sensitive glp-1 mutant, which lacks a germline, is long-lived at the non-permissive temperature in *C. elegans* .	Antagonism	Yes	Group 1	GenAge,SynergyAge	Tumor suppressor gene,<br>NOTCH1 inhibitory antibody was investigated in metastatic colorectal cancer (p1 completed, NCT03031691)	PMID: 22291607, PMID: 26578790, PMID: 21906946
KRAS	Hydrolase	Yes	HC	34	9	44.9	3	Altered intercellular communications, Genomic instability, Mitochondrial dysfunction	20	1	19	9	9	7	2	Antagonism	Not reported	No evidence	NA	Yes	Group 4	-	Antagonist for cancer.<br>KRAS inhbitor such as sotorasib was approved for cancer<br>Genetic inhibition of Ras was found to extend lifespan.<br>Adult-onset administration of the drug trametinib inhibiting MAP2K1 and MAP2K2, a highly specific inhibitor of Ras-Erk-ETS signaling, extended lifespan in *Drosophila*.	PMID: 29276789, PMID: 26119340
HDAC1	Hydrolase	Yes	HC	35	9	47.6	7	Altered intercellular communications, Cellular senescence, Epigenetic shift, Extracellular matrix stiffness, Genomic instability, Inflammation, Stem cell exhaustion	24	6	18	2	10	9	1	Antagonism	Reported; Lifespan extension	Anti-Longevity:<br>Hypomorphic mutation increased lifespan by 33 - 52% in *Drosophila* .<br>Heterogeneous mutation increased male lifespan by 41 - 47%, while no change in median of female lifespan.<br>Rpd3 activity is reduced by calorie restriction, which causes the increase in Sir2 activity and lifespan extension in *Drosophila*.	Antagonism	Yes	Group 1	GenAge,SynergyAge	Antagonist for cancer. HDAC1 inhibitors, vorinostat and romidepsin were approved for the treatment of cutaneous T-cell lymphoma.<br>Inactivating histone deacetylase HDA promotes longevity by mobilizing trehalose metabolism.	PMID: 12459580, PMID: 33790287, PMID: 10512855, PMID: 15520384
VDR	Nuclear receptor	Yes	HC	36	9	48.0	1	Genomic instability	14	1	13	0	7	6	1	Agonism*	Reported; Lifespan extension	Pro-Longevity:<br>Gain-of-function (rh273/274) daf-12 (VDR) increased lifespan significantly while loss-of-function (rh61rh411) daf-12 decreased lifespan significantly.<br>Double mutation daf-2 (IGF-1) and daf-12 increased lifespan with a synergistic effect but single null mutation daf-12 shorten lifespan.<br>Deletion nhr-8(ok186) extended lifespan in *C. elegans*.<br>However, two other studied showed deletion nhr-	Agonism	Yes	Group 1	GenAge,SynergyAge	VDR agonists were investigational for cancer.<br>VDR knockout mice showed several aging related phenotypes, including poorer survival.	PMID: 16626392, PMID: 7789761, PMID: 19500727, PMID: 24655420, PMID: 25209682, PMID: 30125273
TERT	Transferase	Yes	HC	37	9	54.1	8	Altered intercellular communications, Cellular senescence, Genomic instability, Impaired proteostasis, Mitochondrial dysfunction, Retrotranspositions, Stem cell exhaustion, Telomere	3	1	2	6	7	6	1	Antagonism	Reported; Lifespan extension	Pro-Longevity:<br>TERT overexpression (K5-mTert) increased lifespan by 10% in mice.<br>Tert overexpressed cancer-resistant mice increased lifespan by 18%(SP53/TgTert) and 38%(Sp53/Sp16/SArfl Tg Tert mice)<br>Tert knock-in mice significantly increased lifespan, and miss Tert(TERT-ER) knock-in mice signigicantly decreased lifespan.	Agonism	No	-	GenAge	< 7 tissues (out of 47) dysregulated during aging<br>Overexpression of telomerase can inhibit aging but at the expense of increased tumorigenesis	PMID: 17876321, PMID: 23211840, PMID: 15688016 PMID: 19013273, PMID: 21113150
TLR4	Receptor	Yes	HC	38	8	25.3	8	Altered intercellular communications, Deregulated nutrient signaling, Epigenetic shift, Extracellular matrix stiffness, Genomic instability, Inflammation, Retrotranspositions, Stem cell exhaustion	16	10	6	6	8	3	5	Antagonism*	Not reported	No evidence	NA	Yes	Group 4	-	TLR4 is an important Pattern Recognition Receptor (PRR), which activates both innate and adaptive immune cells.<br>Its activation leads to inflammatory cytokine production which is responsible for activating the innate immune system.<br>Additional cytokine production (agonist) may not necessary for the healthy but reduced production (antagonist) may alter immune repsonse<br>The Toll-like receptor 4 (TLR4) signaling pathway is involved in many aspects of biological functions of AML cells, including the regulation of pro-inflammatory cytokine products, myeloid differentiation, and	PMID: 32508811, PMID: 30336978
NME1	Unclassified kinase	No	HC	39	8	39.4	2	Genomic instability, Impaired proteostasis	8	0	8	0	10	10	0	Antagonism	Reported; Lifespan extension	Anti-Longevity: *<br>ndk-1 (RNAi) increased lifespan >5% in *C. elegans* .	Antagonism	Yes	Group 1	GenAge	Antagonist for cancer, as our data showed NME1 upregulation in cancers.<br>However, NME1 is considered as (1) metastasis suppressor and (2) metastasis promotor.<br>NME1 depletion resulted in significantly more rapid migration of nueroblastoma cells<br>NME1 protects against neurotoxin-, α-Synuclein- and LRRK2-induced neurite degeneration in cell models of parkinson's disease.	PMID: 26620638, PMID: 30026594, PMID: 34623600, PMID: 28991262
FGF2	Growth factor	Yes	HC	40	8	43.9	7	Altered intercellular communications, Cellular senescence, Deregulated nutrient signaling, Extracellular matrix stiffness, Inflammation, Stem cell exhaustion, Telomere attrition	14	11	3	6	10	1	9	Antagonism*	Not reported	No evidence	NA	Yes	Group 4	-	FGF2 is a major pro-angiogenic factor during tumor angiogenesis but FGF2 (or bFGF) is neuroprotective for healthy aging.<br>It can improve motor function recovery, increase tyrosine hydroxylase positive neuron survival,<br>and upregulate the levels of neurotransmitters in the brain of a rat model of Parkinson's disease.	PMID: 30274251
CXCR4	GPCR	Yes	HC	41	8	44.0	5	Extracellular matrix stiffness, Genomic instability, Impaired proteostasis, Inflammation, Stem cell exhaustion	23	22	1	6	9	7	2	Antagonism	Not reported	No evidence	NA	Yes	Group 4	-	Antagonist for cancer. CXCR4 overexpression contributes to tumor growth, progression and metastasis.<br>CXCR4 gene deletion in young mesenchymal stem cells accelerates an aging phenotype including increased production of reactive oxygen species, DNA damage, senescence, and reduced proliferation.<br>In constrast, CXCL12/CXCR4 promotes inflammation. Targeting CXCR4 for antiaging may be suitable for aged adults only.	PMID: 26848769, PMID: 32418119
FASN	Acyltransferase	Yes	HC	42	8	48.0	3	Deregulated nutrient signaling, Epigenetic shift, Inflammation	18	1	17	1	7	6	1	Antagonism	Not reported	No evidence	NA	Yes	Group 4	-	Antagonist for cancer.<br>FANS inhibitor prevented the initiation of senescence induction in hematopoietic stem cells and reduced the effect of the activation of senescence on different age-associated diseases.<br>DEHP/DEP (increasing fasn-1 expression, and affecting other lipid metabolic genes) treated *C. elegans* lifespan decreased.	PMID: 30962418, PMID: 29020644
PDGFRB	Receptor kinase	Yes	HC	43	8	48.4	6	Cellular senescence, Deregulated nutrient signaling, Extracellular matrix stiffness, Genomic instability, Impaired proteostasis, Stem cell exhaustion	18	12	6	6	10	5	5	Antagonism	Not reported	No evidence	NA	Yes	Group 4	-	Antagonist for cancer.<br>PDGFR-β signaling in the cardiomyocyte may regulate angiogenesis in the heart in response to load-induced stress through several different mechanisms.<br>Imatinib mesylate inhibiting PDGFRB, ABL1, KIT, BCR reduced lifespan in *C. elegans* .	PMID: 20071776, PMID: 3201088

MDM2	Acyltransferase	Yes	HC	44	8	64.0	3	Cellular senescence, Genomic instability, Impaired proteostasis	13	9	4	7	7	6	1	Antagonism	Not reported	No evidence	NA	Yes	Group 4	-	Antagonist for cancer; oncoprotein Mdm2 in cancer<br>MDM2 is a negative regulator of p53, a tumor-suppressor gene.<br>Dominant-negative versions of *Drosophila* melanogaster p53 in adult neurons extends lifespan.<br>Taken together, antagonizing MDM2 may result in increased p53 activities, which in turn contribute to accelerated aging.<br>However, small-molecule MDM2 antagonists attenuate the senescence-associated secretory phenotype.<br>Ubiquitous overexpression of wild-type p53 in adult flies also shortened lifespan in females but increased life span in males.<br>MDM2 antagonist (MI-319) increased lifespan in mice with severe combined immunodeficiency.	PMID: 29402901, PMID: 16303568, PMID: 21779512, PMID: 19958544
TGFBR2	Receptor kinase	No	HC	45	7	35.4	5	Altered intercellular communications, Deregulated nutrient signaling, Extracellular matrix stiffness, Genomic instability, Stem cell exhaustion	12	4	8	7	8	2	6	Antagonism*	Reported; Lifespan extension	Anti-Longevity: *<br>daf-1 mutation in adults increased mean lifespan by up to 120% and maximum lifespan by up to 185% in *C. elegans*.	Antagonism	Yes	Group 1	GenAge	Tumor suppressor gene<br>Tgfbr2-deficient tumors enhanced the survival and reduced tumor weight	PMID: 17900898, PMID: 31609088
BUB1B	Protein kinase	No	HC	46	7	37.7	2	Genomic instability, Impaired proteostasis	15	2	13	4	11	11	0	Antagonism	Reported; Lifespan extension	Pro-Longevity: *<br>T23 mice (BUB1B overexpression) increased lifespan in mice;<br>Hypomorphic BUB1B mice decreased lifespan by 50% in mice.	Agonism	Yes	Group 2	GenAge	Tumor suppressor gene	PMID: 23242215, PMID: 15208629
CASP8	Peptidase	Yes	HC	47	7	39.4	6	Altered intercellular communications, Epigenetic shift, Genomic instability, Impaired proteostasis, Inflammation, [illegible]	13	5	8	7	11	11	0	Antagonism	Reported; Lifespan extension	Anti-Longevity: *<br>RNA interference (ced-3) in adulthood resulted in a 19% increase in mean lifespan in *C. elegans*.	Antagonism	Yes	Group 1	GenAge	Tumor suppressor gene<br>Caspase-8 may either potentiate or suppress tumor malignancy.<br>Upon activation, its main function is to promote apoptosis.	PMID: 17411345, PMID: 20817393
AURKB	Protein kinase	Yes	HC	48	7	43.6	5	Cellular senescence, Genomic instability, Inflammation, Stem cell exhaustion, Telomere attrition	11	1	10	0	11	11	0	Antagonism	Not reported	No evidence	NA	Yes	Group 4	-	Antagonist for cancer.<br>AURKA is involved in cell cycle checkpoing during healthy aging	
MAP2K1	Protein kinase	Yes	HC	49	7	43.6	3	Cellular senescence, Mitochondrial dysfunction, Stem cell exhaustion	20	0	20	10	9	6	3	Antagonism	Reported; Lifespan extension	Anti-Longevity:<br>Trametinib inhibiting MAP2K1 increased lifespan by 12% in *Drosophila.*	Antagonisum	Yes	Group 1	.ge,GenAge, Geroprotec	Antagonist for cancer.<br>Constitutive expression of MEK1 (MAP2K1) caused cells to senescence.	PMID: 20624915, PMID: 10558995, PMID: 26119340, PMID: 14701721
CREBBP	Acyltransferase	Yes	HC	50	7	44.1	6	Altered intercellular communications, Cellular senescence, Epigenetic shift, Extracellular matrix stiffness, Genomic instability, Impaired proteostasis	13	0	13	7	4	2	2	Antagonism*	Reported; Lifespan extension	Anti-Longevity: *<br>RNA interference increased mean lifespan up to 18% in *C. elegans* (dependent upon functional daf-16).;<br>Knockout mutants lived up to 33% longer in *C. elegans* (dependent upon functional daf-16).<br>cep-1 deletion (gk138) /(lg12501) increased lifespan in *C. elegans*.	Antagonism	Yes	Group 1	GenAge,SynergyAge	Tumor suppressor gene<br>Both TSG and oncogenetic roles have been reported<br>CREBBP inhibitor was investigated in colorectal adenocarcinoma (p2 withdrawn, NCT02413853)	PMID: 17895432, PMID: 19416129
TOP2A	Isomerase	Yes	HC	51	7	45.6	5	Cellular senescence, Epigenetic shift, Genomic instability, Impaired proteostasis, Stem cell exhaustion	17	1	16	0	11	11	0	Antagonism	Not reported	No evidence	NA	Yes	Group 4	-	Antagonist for cancer.<br>TOP2A is involved in cell cycle regulation during healthy aging.<br>Topoisomerases regulate the topological states of DNA and important for neuron proliferation	PMID: 7980433
MAPK1	CMGC kinase	Yes	HC	52	7	46.3	7	Altered intercellular communications, Cellular senescence, Genomic instability, Impaired proteostasis, Mitochondrial dysfunction, Stem cell exhaustion, Telomere [illegible]	27	0	27	9	7	4	3	Antagonism	Reported; Reduced lifespan onl	*Pro-Longevity:*<br>*Mpk-1 RNAi decreased lifespan in C. elegans*	NA	Yes	Group 3	GenAge	Antagonist for cancer.<br>Age-associated selective impairment in the MAPK signaling pathways in the aged brain.<br>Lifelong caloric restriction completely prevented the age-related decrease in basal brain ERK activity and diminished the age-related reduction of p38 MAPK activity.	PMID: 20624915, PMID: 10647963, PMID: 10558995
EP300	Acyltransferase	Yes	HC	53	7	50.4	8	Altered intercellular communications, Cellular senescence, Epigenetic shift, Extracellular matrix stiffness, Genomic instability, Impaired proteostasis, Inflammation, Mitochondrial dysfunction	23	0	23	6	6	4	2	Antagonism	Reported; Lifespan extension	Anti-Longevity: *<br>RNA interference (cep-1) increased mean lifespan up to 18% in *C. elegans* (dependent upon functional daf-16).<br>Knockout mutants lived up to 33% longer in *C. elegans* (dependent upon functional daf-16).<br>RNAi resulted in a mean and maximal lifespan increased by 20% and 10% respectively in *C. elegans*.	Antagonism	Yes	Group 1	GenAge	Tumor suppressor gene	PMID: 17895432, PMID: 17895432, PMID: 20346071
ERBB3	Receptor kinase	Yes	HC	54	7	51.3	4	Cellular senescence, Deregulated nutrient signaling, Genomic instability, Impaired proteostasis	11	3	8	5	11	9	2	Antagonism	Reported; Lifespan extension	Pro-Longevity:<br>Let-23 mutants (reduction-of-function) survive less robustly in middle adulthood than matched wild type controls (19% decrease of median lifespan, 8% decrease of maximum lifespan) (PMID: 20497132).<br>Let-23 mutants (gain-of-function) survive more robustly in middle adulthood (29% increase of median lifespan, 9% increase of maximum lifespan (PMID: 20497132)"	Agonism	Yes	Group 2	GenAge	Antagonist for cancer.<br>ERBB3 is necessary for maturation and myelination of oligodendrocytes, specialized glial cells that myelinate CNS axons	PMID: 35370556, PMID: 20497132
NRAS	Hydrolase	No	HC	55	7	54.4	0	-	25	0	25	9	9	9	0	Antagonism	Reported; Reduced lifespan onl	Anti-Longevity:<br>let-60 gain-of-function significantly reduced lifespan in *C. elegans*.	NA	No	-	GenAge	Target did not associate with any aging hallmarks	PMID: 16164423
KDM1A	Oxidoreductase	Yes	HC	56	7	54.9	6	Cellular senescence, Deregulated nutrient signaling, Epigenetic shift, Genomic instability, Impaired proteostasis, Stem cell exhaustion	27	1	26	0	10	10	0	Antagonism	Not reported	No evidence	NA	Yes	Group 4	-	Antagonist for cancer.<br>KDM1A, is a drug target of IMG-7289, lysine-specific demethylase 1 inhibitor, investigated for the treatment of leukemia in trial (NCT02842827).<br>Modulation of KDM1A with vafidemstat (KDM1A inhibitor) rescues memory deficit and behavioral alterations in mice	PMID: 32469975
PRKDC	Protein kinase	Yes	HC	57	7	55.1	9	Altered intercellular communications, Cellular senescence, Deregulated nutrient signaling, Epigenetic shift, Genomic instability, Impaired proteostasis, Inflammation, Stem cell exhaustion, Telomere [illegible]	29	0	29	0	11	11	0	Antagonism	Reported; Lifespan extension	Pro-Longevity:<br>mei-41 overexpression increased lifespan in *Drosophila*.<br>mei-41 mutated decreased lifespan in *Drosophila*.<br>Diminished lifespan and acute stress-induced death in DNA-PKcs-deficient mice with limiting telomeres.<br>However, a study demonstrated the anti-longevity of PRKDC in *C. elegans,* and atl-1 deletion [illegible]	Agonism	Yes	Group 2	-	Antagonist for cancer.<br>PRKDC is invovled in DNA double-strand break repair and innate immune system during healthy aging	PMID: 6411485, PMID: 14528032, PMID: 23434802, PMID: 17072335
FGFR2	Receptor kinase	Yes	HC	58	7	58.7	4	Altered intercellular communications, Extracellular matrix stiffness, Genomic instability, Stem cell exhaustion	16	4	12	6	9	2	7	Antagonism*	Not reported	No evidence	NA	Yes	Group 4	-	Antagonist for cancer but our data showed FGFR2 downregulation in cancer.<br>FGFR2 is important for cell division, cell maturation, formation of new blood vessels, wound healing, and bone growth and development	
EZH2	Methyltransferase	Yes	HC	59	7	59.3	4	Cellular senescence, Epigenetic shift, Inflammation, Stem cell exhaustion	9	3	6	9	11	11	0	Antagonism	Reported; Lifespan extension	Anti-Longevity:<br>Heterogeneous mutation at amino acid 63/731 increased lifespan by 71% - 76%;<br>while heterogenous mutation at amino acid 63 increased lifespan by 33% in *Drosophila*.<br>EZH2 mutants were long-lived due to increased resistance to oxidative stress and starvation in *Drosophila*.<br>RNAi mes-2 increased lifespan by 6.5% in *C. elegans*.	Antagonism	Yes	Group 1	GenAge,SynergyAge	EZH2 is considered as oncogene in cancer.<br>Inhibition of EZH2 attenuates neuroinflammation<br>Microglial EZH2 inhibition leads to neuroprotection after stroke in aged mice	PMID: 20018689, PMID: 32933418, PMID: 22212395, https://www.ahajournals.org/doi/10.1161/str.52.suppl 1.P759
PPARA	Nuclear receptor	Yes	HC	60	7	61.6	11	Altered intercellular communications, Deregulated nutrient signaling, Epigenetic shift, Extracellular matrix stiffness, Genomic instability, Impaired proteostasis, Inflammation, Mitochondrial dysfunction, Retrotranspositions, Stem [illegible]	22	1	21	2	8	1	7	Agonism	Reported; Lifespan extension	Pro-Longevity:<br>Clofibrate and Fenofibrate agonising PPARA extended lifespan in *C. elegans*.	Agonism	Yes	Group 1	DrugAge,Geroprotector	Agonist for cancer.<br>PPARγ agonists delay age-associated metabolic disease and extend longevity..<br>Bezafibrate agonising PPARA, PPARD and PPARG extended lifespan in *C. elegans.*<br>Bezafibrate, clofibrate, bezafibrate,<br>and fenofibrate failed to extend lifespan in *C. elegans* in absence of NHR-49	PMID: 33219735, PMID: 23603800

HRAS	Hydrolase	No	HC	61	7	62.9	4	Cellular senescence, Extracellular matrix stiffness, Genomic instability, Inflammation	17	10	7	10	8	7	1	Antagonism	Reported; Reduced lifespan only	Pro-Longevity:<br>let-60(n1046gf) mutation decreased lifespan in *C. elegans* .	NA	Yes	Group 3	GenAge	Antagonist for cancer.<br>Deregulation of the Egfr/Ras signaling pathway induces age-related brain degeneration in the *Drosophila*.<br>Expression of HRAS in lung fibroblasts resulted in a permanent G1 arrest, accompanied by accumulation of senescence associated factors, p53 and p16.	PMID: 12529440, PMID: 9054499, PMID: 16164423
ABL1	Tyrosine kinase	Yes	HC	62	7	70.7	9	Altered intercellular communications, Cellular senescence, Epigenetic shift, Extracellular matrix stiffness, Genomic instability, Impaired proteostasis, Inflammation, Mitochondrial [illegible]	16	7	9	7	9	5	4	Antagonism	Not reported	No evidence	NA	Yes	Group 4		Antagonist for cancer but ABL1 is important for DNA double-strand break repair during healthy aging.<br>Senolytic treatment reduces cell senescence. Quercetin, senolytic drug, inhibits multiple kinases including ABL1.<br>Pro-Longevity:<br>Imatinib mesylate inhibiting ABL1 reduced lifespan in *C. elegans*.	PMID: 3201088
SIRT1	Acyltransferase	Yes	HC	63	7	73.3	12	Altered intercellular communications, Cellular senescence, Deregulated nutrient signaling, Epigenetic shift, Extracellular matrix stiffness, Genomic instability, Impaired proteostasis, Inflammation, Mitochondrial dysfunction, Retrotranspositions, Stem cell exhaustion, Telomere attrition	15	2	13	3	8	1	7	Antagonism*	Reported; Lifespan extension	Pro-Longevity:<br>sir-2.1 overexpression increased lifespan significantly in *C. elegans* .<br>Brain overexpression SIRT1 increased lifespan by 11% in mice.<br>Overexpressed-SIRT1 mice were more metabolically active (i.e. reduction in bloodd cholesterol, insulin, and fasted glucose).<br>sir-2.1 deletion sligtly decreased lifespan in mice.<br>Moderate overexpression of Sir2 in the fat body increased lifespan by 12% in male *Drosophila* and 13% in female *Drosophila* .<br>Loss of dSir2 decreased lifespan by 5.9% and 26.1% in *Drosophila* .<br>Overexpression of dSir2 increased lifespan by 57% in *Drosophila* .<br>mTERT treatment increased sirt1 expression and extended lifespan in mice.<br>However, two studies showed SIRT1 overexpression did not affect lifespan in mice, and overexpression of dSir2 did not result in increased lifespan in *Drosophila* .<br>Two 14-3-3 proteins (SIR-2.1 binding partners) were required for the lifespan extension conferred by extra copies of sir-2.1 in *C. elegans* .<br>Overexpressing sir-2.1 confered a lifespan [illegible]	Agonism	Yes	Group 2	GenAge, SynergyAge	Antagonist for cancer, although our data showed that there were downregulation in 4 cancers.<br>SIRT1 can act as an oncogene or tumor suppressor in cancer<br>Inhibition of SIRT1 activity would be beneficial for cancer treatment.<br>SIRT1 could reduce p53-mediated apoptosis.<br>High levels of SIRT1 expression as a protective mechanism against disease-related conditions.	PMID: 11242085, PMID: 16280150, PMID: 20975665, PMID: 24011076, PMID: 17877786 PMID: 24020000, PMID: 30374331, PMID: 21938067, PMID: 23765091, PMID: 17159295, PMID: 15520384, PMID: 19173036, PMID: 16777605, PMID: 21909281, PMID: 34389865
PTPRC	Receptor phosphatas	Yes	HC	64	7	81.4	4	Altered intercellular communications, Genomic instability, Inflammation, Stem cell [illegible]	15	10	5	3	8	5	3	Antagonism*	Reported; reduced lifespan only	Pro-Longevity:<br>ptprc-/- decreased lifespan in mice.	NA	Yes	Group 3	-	Tumor suppressor gene<br>BC8 131I, antibody drug & PTPRC binding agent, was investigated in clincial trials for cancer treatment.	PMID: 30820040
TYMS	Methyltransferase	Yes	HC	65	6	33.2	3	Inflammation, Mitochondrial dysfunction, Stem cell exhaustion	14	8	6	0	11	11	0	Antagonism	Reported; Lifespan extension	Anti-Longevity:<br>Floxuridine (400 μM) extended lifespan in *C. elegans* , while a higher dose i.e. 5000 μM shortened their lifespans.	Antagonism	Yes	Group 1	DrugAge	Antagonist for cancer.<br>TYMS is involved in cell cycle regulation during healthy aging.	PMID: 153363
RAC1	Hydrolase	No	HC	66	6	38.5	6	Altered intercellular communications, Deregulated nutrient signaling, Epigenetic shift, Extracellular matrix stiffness, Genomic instability, Inflammation	16	1	15	9	10	9	1	Antagonism	Not reported	No evidence	NA	Yes	Group 4	-	Antagonist for cancer.<br>RAC1 is involved in adaptive immune system and innate immune system during healthy aging.	
SRC	Tyrosine kinase	Yes	HC	67	6	47.5	11	Altered intercellular communications, Cellular senescence, Deregulated nutrient signaling, Epigenetic shift, Extracellular matrix stiffness, Genomic instability, Impaired proteostasis, Inflammation, Mitochondrial dysfunction, Stem cell [illegible]	10	3	7	6	8	7	1	Antagonism	Not reported	Not reported	NA	Yes	Group 4	-	Antagonist for cancer. Dasatinib, SRC inhibitor, was used for cancer treatment.<br>Dasatinib was also considered as senolytic used to remove senescent cell.	
CDC25A	Esterase	No	HC	68	6	49.3	2	Genomic instability, Impaired proteostasis	16	1	15	1	11	11	0	Antagonism	Reported; Lifespan extension	Anti-Longevity:<br>cdc-25.3 (RNAi) significantly increased lifespan in *C. elegans* .	Antagonism	Yes	Group 1	GenAge	Antagonist for cancer. CDC25A is thought to be a proto-oncogene.<br>CDC25A is invovled for cell cycle regulation.	PMID: 16741121, PMID: 15021892
IL1B	Interleukin	Yes	HC	69	6	49.5	8	Altered intercellular communications, Deregulated nutrient signaling, Epigenetic shift, Extracellular matrix stiffness, Genomic instability, Impaired proteostasis, Inflammation, Telomere [illegible]	9	1	8	6	6	3	3	Antagonism	Not reported	No evidence	NA	Yes	Group 4	-	IL-1α and IL-1β signal upregulate the senescence-associated secretory phenotype in a cooperative manner.<br>IL-1β promotes the age-associated decline of beta cell function.	PMID: 30988157, PMID: 34746709
FGFR1	Receptor kinase	Yes	HC	70	6	49.8	5	Cellular senescence, Deregulated nutrient signaling, Extracellular matrix stiffness, Genomic instability, Telomere attrition	16	11	5	7	8	1	7	Antagonism*	Not reported	No evidence	NA	Yes	Group 4	-	FGFR1 antagonists such as pemigatinib approved for cancer (NCT03011372) but our data showed FGFR1 upregulation in cancers.<br>Mice with an attenuation of FGFR1 signalling develop diabetes with age and exhibit a decreased number of beta-cells,<br>and lower levels of FGFR1 in mice have also been related to craniofacial defects.	PMID: 12514106, PMID: 11130726
KIT	Receptor kinase	Yes	HC	71	6	51.8	6	Altered intercellular communications, Cellular senescence, Deregulated nutrient signaling, Genomic instability, Inflammation, Stem cell [illegible]	17	5	12	6	10	2	8	Antagonism*	Not reported	No evidence	NA	Yes	Group 4	-	Antagonist for cancer although our data showed downregulation in cancer.<br>Adult myocardium relies on c-kit expression to regenerate after injury and to counteract aging effects on cardiac structure and function.<br>Imatinib mesylate inhibiting PDGFRB, ABL1, KIT, BCR reduced lifespan in *C. elegans*.	PMID: 31275242, PMID: 32010883
RARB	Nuclear receptor	Yes	HC	72	6	54.8	2	Genomic instability, Stem cell exhaustion	14	6	8	0	10	1	9	Agonism	Not reported	No evidence	NA	Yes	Group 4	-	Agonist for cancer.<br>RAR agonist ameliorated the UV-induced damage to skin collagen fibers, and increased the collagen content in photoaged skin through RAR.	PMID: 28849147
MAPK8	CMGC kinase	Yes	HC	73	6	57.0	6	Cellular senescence, Epigenetic shift, Genomic instability, Impaired proteostasis, Mitochondrial dysfunction, Stem cell exhaustion	16	1	15	0	7	3	4	Antagonism	Reported; Lifespan extension	Pro-Longevity:<br>loss-of-funtion jnk-1 significantly decreased lifespan in *C. elegans*.<br>jnk-1 overpression (lpIn2) increased lifespan in *C. elegans*.<br>jnk-1 deficient mice (gk1) decreased lifespan.<br>Bsk RNAi decreased lifespan by 16.4% in male and 10.2% in female *Drosophila* .	Agonism	Yes	Group 2	GenAge, SynergyAge	Antagonist for cancer.<br>Targeting JNK by RNA interference and inhibitor inhibit the development of hepatocellular carcinoma.<br>MAPK8, also known as JNK1. Fruit flies with mutations that augment JNK signalling live longer.<br>Overexpression of JNK in roundworms also increases lifespan.<br>MAPK8 inhibitor was investigated in in myeloid leukemia (p1 terminated, NCT00126893)	PMID: 28012230, PMID: 15767565, PMID: 18832074, PMID: 20976250
CTSB	Peptidase	No	HC	74	6	58.8	3	Extracellular matrix stiffness, Genomic instability, Impaired proteostasis	17	0	17	1	11	8	3	Antagonism	Not reported	No evidence	NA	Yes	Group 4	-	Antagonist for cancer.<br>Inhibitors of cathepsin B improve memory and reduce beta-amyloid in transgenic Alzheimer disease mice.<br>Nucleus distribution of cathepsin B in senescent microglia promotes brain aging through degradation of sirtuins.	PMID: 18184658, PMID: 33049518
DNM2	Hydrolase	No	HC	75	6	61.2	5	Altered intercellular communications, Cellular senescence, Deregulated nutrient signaling, Extracellular matrix stiffness, Genomic instability	13	3	10	3	10	6	4	Antagonism	Not reported	No evidence	NA	Yes	Group 4	-	Tumor suppressor gene	
RHOA	Hydrolase	No	HC	76	6	63.5	5	Altered intercellular communications, Epigenetic shift, Genomic instability, Inflammation, Stem cell exhaustion	11	1	10	9	6	3	3	Antagonism	Not reported	No evidence	NA	Yes	Group 4	-	Antagonist for cancer.<br>RHOA is invovled in protecting against the progression of cardiac aging.	https://escholarship.org/uc/item/14g0843j

JAK2	Tyrosine kinase	Yes	HC	77	6	67.2	9	Altered intercellular communications, Cellular senescence, Deregulated nutrient signaling, Epigenetic shift, Extracellular matrix stiffness, Genomic instability, Inflammation, Retrotranspositions, Stem cell exhaustion	14	3	11	6	8	1	7	Antagonism*	Reported; Lifespan extension	Anti-Longevity:<br>Heterogenous gain-of-function decreased lifespan significantly in *Drosophila* .<br>Heterogenous loss-of-function increased lifespan significantlly increased in *Drosophila* .	Antagonism	Yes	Group 1	GenAge	Ruxolitinib, JAK2 antagonist was investigated for cancer treatment.<br>Inhibition of the JAK pathway partially restored age-related decline in coordination.	PMID: 22291607,<br>PMID: 26578790
AKT3	AGC kinase	Yes	HC	78	6	71.3	3	Cellular senescence, Mitochondrial dysfunction, Retrotranspositions	25	6	19	9	10	3	7	Antagonism*	Not reported	No evidence	NA	Yes	Group 4	-	AKT3 antagonist such as Capivasertib investigated for cancer but our data showed AKT3 downregulation in cancer.<br>Aging-induced Akt activation involves in aging-related pathologies and Aβ-induced toxicity	PMID: 31183966
PKM	Non-protein kinase	No	HC	79	6	73.2	5	Altered intercellular communications, Deregulated nutrient signaling, Epigenetic shift, Extracellular matrix stiffness, Mitochondrial dysfunction	16	1	15	0	10	10	0	Antagonism	Not reported	No evidence	NA	Yes	Group 4	-	Antagonist for cancer.<br>PKM is involved in innate immune system during healthy aging.<br>In addtion, loss of PKM2 impairs angiogenic sprouting and loss of endothelial PKM2 alters mitochondrial metabolism.	PMID: 30301887
PGR	Nuclear receptor	Yes	HC	80	5	22.8	2	Altered intercellular communications, Mitochondrial dysfunction	17	12	5	0	9	0	9	Agonism	Reported; Lifespan extension	Pro-Longevity:<br>Danazol agonising PGR & AR extended lifespan in *C. elegans*.	Agonism	Yes	Group 1	DrugAge,Geroprotector	PGR agonists were investigated for cancer treatment such as breast cancer, endometrial cancer, prostate cancer, liver cancer.<br>Progesterone has multiple non-reproductive functions in the central nervous system to regulate cognition, mood, inflammation, mitochondrial function, neurogenesis and regeneration, myelination and recovery from traumatic brain injury.	PMID: 18374402,<br>PMID: 24134630
NCOA3	Acyltransferase	No	HC	81	5	37.2	2	Epigenetic shift, Stem cell exhaustion	17	2	15	1	10	10	0	Antagonism	Not reported	No evidence	NA	Yes	Group 4	-	Antagonist for cancer. NOCA3 (known as AIB1 or RAC3) coactivates E2F1 and promotes breast cancer cell proliferation.<br>NCOA3 promotes bladder cancer cell proliferation through AKT and E2F1.<br>Inhibition of NCOA3 could prevent the development of fibrosis, and ameliorate pre-established fibrosis.<br>NOCA3 overexpression in the non-tumoral HEK293 cells inhibits the premature senescence induced by hydrogen peroxide or rapamycin. The mechanism involves (1) the inhibition of autophagy and (2) the increase in levels and nuclear localization of both the cell cycle suppressors p53/p21 and the longevity promoters FOXO1A, FOXO3A and SIRT1.<br>NCOA3 overexpression is required in order to maintain the telomerase activity.<br>NCOA3 is an inhibitor of senescence whose downregulation in aged individuals could be probably a tumor suppressor mechanism, preventing the clonal expansion of risky old cells from having damaged DNA.	PMID: 23511556,<br>PMID: 26469953
NCOA1	Acyltransferase	No	HC	82	5	43.6	5	Altered intercellular communications, Deregulated nutrient signaling, Epigenetic shift, Genomic instability, Mitochondrial dysfunction	23	6	17	1	8	1	7	Antagonism*	Not reported	No evidence	NA	Yes	Group 4	-	Antagonist for cancer. NCOA1 promotes angiogenesis in breast tumors.<br>Aging or ER antagonists were shown to downregulate NCOA1 in the hippocampus of female mice.<br>17β-estradiol treatment can upregulate NCOA1. Beta-Estradiol was shown to increase lifespan in *C. elegans* .<br>Taken together, NOCA1 agonist may delay aging.<br>NCOA1 is required for the anti-obesogenic effects of 17β-estradiol.	PMID: 26287601,<br>PMID: 24134630
ATM	Protein kinase	No	HC	83	5	44.4	10	Cellular senescence, Deregulated nutrient signaling, Extracellular matrix stiffness, Genomic instability, Impaired proteostasis, Inflammation, Mitochondrial dysfunction, Retrotranspositions, Stem [illegible]	13	11	2	7	7	4	3	Antagonism	Reported; Lifespan extension	Pro-Longevity:<br>Lifespan of Terc-/- Atm-/- significantly decreased comparing to Terc-/- atm+/+ late generation mice.<br>Mutations in ATM in late-generation TERC mutants with short telomeres resulted in signs of premature ageing<br>Anti-Longevity:<br>Deletion allele atm-1(gk186) increased lifespan by 20% in *C. elegans* .	nism / Antagon	Yes	Group 2	SynergyAge,GenAge	Tumor suppressor gene<br>Low dose of chloroquine (inhibiting multiple genes/proteins) activates ATM, promoted DNA damage clearance,<br>rescued age-related metabolic shift, and prolonged replicative lifespan in mice.	PMID: 12540856,<br>PMID: 19416129
EphA2	Receptor kinase	Yes	HC	84	5	47.6	5	Cellular senescence, Extracellular matrix stiffness, Genomic instability, Inflammation, Telomere attrition	8	5	3	4	10	4	6	Antagonism	Reported; Lifespan extension	Pro-Longevity:<br>vab-1 overexpression (dx31) increased lifespan by 20% in *C. elegans*.	Agonism	Yes	Group 2	GenAge	Antagonist for cancer.<br>EphA2 as a promoter of melanoma tumorigenicity	PMID: 19853560,<br>PMID: 19223760
HDAC2	Hydrolase	Yes	HC	85	5	48.6	7	Altered intercellular communications, Epigenetic shift, Extracellular matrix stiffness, Genomic instability, Impaired proteostasis, [illegible]	23	1	22	1	9	9	0	Antagonism	Not reported	No evidence	NA	Yes	Group 4	-	Antagonist for cancer. HDAC2 inhibitors, vorinostat and romidepsin were approved for the treatment of cutaneous T-cell lymphoma.<br>Inactivating histone deacetylase HDA promotes longevity by mobilizing trehalose metabolism.	PMID: 33790287
RARA	Nuclear receptor	Yes	HC	86	5	50.6	6	Altered intercellular communications, Cellular senescence, Epigenetic shift, Genomic instability, Inflammation, Stem cell exhaustion	13	10	3	0	8	4	4	Agonism	Not reported	No evidence	NA	Yes	Group 4	-	RARA agonist for cancer.<br>RAR agonist ameliorated the UV-induced damage to skin collagen fibers, and increased the collagen content in photoaged skin through RAR.	PMID: 28849147
AKT2	AGC kinase	Yes	HC	87	5	57.8	7	Altered intercellular communications, Deregulated nutrient signaling, Epigenetic shift, Genomic instability, Mitochondrial dysfunction, Retrotranspositions, [illegible]	17	5	12	9	7	4	3	Antagonism	Reported; Lifespan extension	Anti-Longevity:<br>AKT2 knockout mutants increased lifespan by 9.1% in mice.	Antagonism	Yes	Group 1	GenAge	Aging-induced Akt activation involves in aging-related pathologies and Aβ-induced toxicity	PMID: 28681509,<br>PMID: 31183966
DNMT3A	Methyltransferase	Yes	HC	88	5	60.0	3	Deregulated nutrient signaling, Epigenetic shift, Genomic instability	16	3	13	7	11	11	0	Antagonism	Not reported	No evidence	NA	Yes	Group 4	-	Constitutive loss of DNMT3A causes morbid obesity through misregulation of adipogenesis	PMID: 35635747
TNF	umour necrosis fact	Yes	HC	89	5	61.6	11	Altered intercellular communications, Cellular senescence, Deregulated nutrient signaling, Epigenetic shift, Extracellular matrix stiffness, Genomic instability, Impaired proteostasis, Inflammation, Mitochondrial dysfunction, Stem cell [illegible]	9	1	8	8	7	4	3	Antagonism	Not reported	No evidence	NA	Yes	Group 4	-	TNF antgonist were invesitagted for cancer treatment but our data showed TNF downregulation in cancer.<br>TNF-α antagonism rescues the effect of ageing on stroke; TNF-α/IFN-γ synergy amplifes senescence-associated inflammation.<br>TNF-deficient mice develop normally but are more susceptible to some infectious agents.	PMID: 34076259,<br>PMID: 35645319
PLAU	Peptidase	No	HC	90	5	62.2	2	Extracellular matrix stiffness, Impaired proteostasis	8	4	4	6	10	9	1	Antagonism	Reported; Lifespan extension	Pro-Longevity:<br>uPA overexpression (alphaMUPA) increased lifespan by 20% in mice.<br>Transgenic mice overexpressing urokinase-type plasminogen activator increased longevity.	Agonism	Yes	Group 2	GenAge	Antagonist for cancer.	PMID: 9060969,<br>PMID: 10638529
ITGAV	Receptor	Yes	HC	91	5	66.2	7	Altered intercellular communications, Cellular senescence, Deregulated nutrient signaling, Epigenetic shift, Extracellular matrix stiffness, Genomic instability, Retrotranspositions	21	7	14	5	10	7	3	Antagonism	Not reported	No evidence	NA	Yes	Group 4	-	Antagonist for cancer.<br>Silencing of ITGAV inhibited cell proliferation, invasion, and self-renewal of breast cancer cell lines.<br>ITGB5 served as a ligand for Cyr61, a molecule stimulating the production of IL-6, which is<br>considered an aging biomarker, via itgav/itgb5/Akt/NF-κB signaling pathway in rheumatoid arthritis	PMID: 32064162,<br>PMID: 17071038,<br>PMID: 22547695

APEX1	Esterase	Yes	HC	92	5	67.8	8	Cellular senescence, Epigenetic shift, Genomic instability, Impaired proteostasis, Inflammation, Mitochondrial dysfunction, Stem cell	27	0	27	1	10	9	1	Antagonism	Reported; Reduced lifespan onl	Pro-Longevity: exo-3 (RNAi) decreased lifespan by 20% in *C. elegans* .	NA	Yes	Group 3	GenAge	Antagonist for cancer. APEX1 is invovled in base excision repair during healthy aging	PMID: 20346071
UBE2C	Acyltransferase	No	HC	93	5	70.8	1	Impaired proteostasis	9	2	7	0	11	11	0	Antagonism	Not reported	No evidence	NA	Yes	Group 4	-	Antagonist for cancer. UBE2C is involved in cell cycle checkpoints during healthy aging	
GSTP1	Transferase	Yes	HC	94	5	71.0	7	Altered intercellular communications, Epigenetic shift, Extracellular matrix stiffness, Genomic instability, Inflammation, Mitochondrial	22	9	13	1	10	6	4	Antagonism	Reported; Lifespan extension	Pro-Longevity: Overexpression of gst-10 increased lifespan by around 22% in *C. elegans*. GSTP1 RNAi extended lifespan in *C. elegans* .	Agonism	Yes	Group 2	GenAge,Paper	GSTP1 antagonist such as ezatiostat was investigated for cancer treatment. GSTP1 overexpression protects against UV light damage.	PMID: 30043549, PMID: 16164425, PMID: 17157356
HMGB1	Chemokine	Yes	HC	95	5	71.0	7	Altered intercellular communications, Cellular senescence, Extracellular matrix stiffness, Genomic instability, Impaired proteostasis, Inflammation,	15	4	11	1	6	4	2	Antagonism	Not reported	No evidence	NA	Yes	Group 4	-	Antagonist for cancer; Inhibition of HMGB1 reduced proliferation in diffuse large B-cell lymphoma. HMGB1 mediates neuroinflammatory priming in the aged brain. Blocking the actions of HMGB1 appears to "desensitize" aged microglia to an immune challenge, thereby preventing exaggerated behavioral and neuroinflammatory responses following infection	PMID: 30988279, PMID: 27466339
PTK2	Tyrosine kinase	Yes	HC	96	5	76.8	9	Altered intercellular communications, Cellular senescence, Deregulated nutrient signaling, Extracellular matrix stiffness, Genomic instability, Impaired proteostasis, Inflammation, Retrotranspositions,	19	5	14	6	9	9	0	Antagonism	Not reported	No evidence	NA	Yes	Group 4	-	Antagonist for cancer. PTK2 is involved in innate immune system during healthy aging.	
DNMT3B	Methyltransferase	No	HC	97	4	37.5	1	Epigenetic shift	6	2	4	1	11	10	1	Antagonism	Not reported	No evidence	NA	No	Group 4	-	Antagonist for cancer as DNMT3B silencing suppresses migration and invasion by epigenetically promoting miR-34a in bladder cancer. DNMT3B plays a protective role against hepatocarcinogenesis caused by chronic inflammation via maintaining mitochondrial homeostasis.	PMID: 33221743, PMID: 33277576
FAS	Receptor	No	HC	98	4	39.3	3	Altered intercellular communications, Genomic instability,	24	23	1	8	10	2	8	Antagonism*	Reported; Reduced lifespan onl	Pro-Longevity Loss of function fas mutated decreased lifespan in mice.	NA	Yes	Group 3	-	Tumor suppressor gene	PMID: 26084385
RRM2	Oxidoreductase	Yes	HC	99	4	43.8	1	Genomic instability	14	2	12	2	11	11	0	Antagonism	Reported; Lifespan extension	Anti-Longevity: rnr-2 (RNAi) increased lifespan by 14.2% in *C. elegans* .	Antagonism	Yes	Group 1	GenAge	Antagonist for cancer. RRM2 is involved in DNA replication during healthy aging	PMID: 23144747
PTGS2	Oxidoreductase	Yes	HC	100	4	44.3	4	Altered intercellular communications, Extracellular matrix stiffness, Genomic instability, Inflammation	9	8	1	7	9	4	5	Antagonism*	Reported; Lifespan extension	Anti-Longevity: Aspirin inhibiting PTGS1 and PTGS2 extended lifespan in mice.	Antagonism	Yes	Group 1	DrugAge,Geroprotector	Increased senescence in PTGS2/COX2 transgenic mice. Cyclooxygenase-2 inhibitors modulate skin aging in a catalytic activity-independent manner.	PMID: 27750221, PMID: 22771771, PMID: 18631321
LYN	Tyrosine kinase	Yes	Novel	1	11	4.5	11	Altered intercellular communications, Cellular senescence, Deregulated nutrient signaling, Epigenetic shift, Extracellular matrix stiffness, Genomic instability, Impaired proteostasis, Inflammation, Mitochondrial dysfunction,	9	2	7	2	7	6	1	Antagonism	Not reported	Not reported	NA	Yes	Group 4	-	Lyn suppresses osteoclastogenesis in vitro and in vivo.	PMID: 19171907
PTPN11	Esterase	Yes	Novel	2	11	13.5	7	Altered intercellular communications, Cellular senescence, Deregulated nutrient signaling, Epigenetic shift, Extracellular matrix stiffness, Impaired proteostasis, Inflammation	21	1	20	8	10	7	3	Antagonism	Reported; reduced lifespan only	Pro-Longevity: ptp-2 RNAi decreased lifespan in *C elegans* .	NA	Yes	Group 3	-	Antagonist for cancer. PTPN11 is involved in adaptive immune system during healthy aging. In addition, the PTPN11 loss-of-function mutation Q510E-Shp2 causes hypertrophic cardiomyopathy by dysregulating mTOR signaling	PMID: 22058153, PMID: 16547100
POLE	Transferase	Yes	Novel	3	11	13.9	2	Cellular senescence, Genomic instability	18	3	15	1	10	10	0	Antagonism	Not reported	No evidence	NA	Yes	Group 4	-	Tumor suppressor gene	
PKMYT1	Protein kinase	No	Novel	4	11	14.5	0	-	16	1	15	0	10	10	0	Antagonism	Not reported	No evidence	NA	No	-		Target did not associate with any aging hallmarks	
HCK	Tyrosine kinase	Yes	Novel	5	11	14.9	4	Extracellular matrix stiffness, Genomic instability, Impaired proteostasis, Inflammation	17	5	12	2	11	6	5	Antagonism	Not reported	No evidence	NA	Yes	Group 4	-	Antagonist for cancer.	
POLA1	Transferase	Yes	Novel	6	11	18.8	2	Cellular senescence, Genomic instability	23	4	19	0	8	8	0	Antagonism	Reported; Reduced lifespan onl	Pro-Longevity: Y47D3A.29 RNAi decreased lifespan by 25.68% in *C. elegans* .	NA	Yes	Group 3	GenAge	Antagonist for cancer. POLA1 is involved in cell cycle regulation and DNA replication initiation.	PMID: 18059442
HDAC10	Hydrolase	Yes	Novel	7	11	19.7	5	Cellular senescence, Epigenetic shift, Genomic instability, Impaired proteostasis, Mitochondrial dysfunction	15	14	1	0	5	4	1	Antagonism	Not reported	No evidence	NA	Yes	Group 4		Antagonist for cancer but HDAC10 was demonstrated to be a tumor suppresser in some types of cancer. Highly tumorigenic and stem-like lung adenocarcinoma cells were increased in Hdac10-deleted tumors compared with Hdac10 wild-type tumors. HDAC10 expression is associated with DNA mismatch repair gene and is a predictor of good prognosis in colon carcinoma Reduced expression of HDAC10 contributes to tumor progression in NSCLC.	PMID: 32540961, PMID: 29085502
IMPDH2	Oxidoreductase	Yes	Novel	8	11	23.2	0	-	5	1	4	0	10	5	5	Antagonism	Reported; No effects on lifespar	Anti-Longevity: Mycophenolic acid inhibiting IMPDH1 and IMPDH2 increased lifespan by 6.08% in *Drosophila* without significance. Mycophenolic acid inhibiting IMPDH1 and IMPDH2 slightly increased lifespan by 6.08% in *C. elegans* without significance.	NA	No	-	-	Target did not associate with any aging hallmarks < 7 tissues (out of 47) dysregulated during aging	PMID: 33008901
PSMD14	Peptidase	Yes	Novel	9	11	23.9	3	Cellular senescence, Genomic instability, Impaired proteostasis	21	1	20	1	11	10	1	Antagonism	Reported; Reduced lifespan onl	Pro-Longevity: post-developmental RNAi of rpt-1 decreased lifespan by 19.3%. rpn-11 RNAi decreased lifespan by 34.3% in *C. elegans* .	NA	Yes	Group 3	GenAge, SynergyAge	Antagonist for cancer. PSMD14 is invovled in cell cycle checkpoint.	PMID: 23144747, PMID:17392428
FES	Tyrosine kinase	No	Novel	10	11	28.4	1	Inflammation	16	13	3	7	10	3	7	Antagonism*	Not reported	No evidence	NA	Yes	Group 4	-	Overexpression of FES could inhibit cell proliferation, migration, and invasion of osteosarcoma cells. However, FES is considered as proto-oncogene, tyrosine kinase, and our data showed FES downregulation in 3 cancers. Taken together, its expression dysregulation is inconsistent with its mechanism of action in cancer. FES kinase activity is also dispensable for hematopoiesis.	PMID: 322556211, PMID: 10523632
PSMB5	Peptidase	Yes	Novel	11	11	32.4	2	Genomic instability, Impaired proteostasis	27	0	27	1	8	8	0	Antagonism	Reported; Lifespan extension	Pro-Longevity: pbs-5 overexpression increase 25% lifespan in *C. elegans* . pbs-5 RNAi decreased lifespan by 29.95% in *C. elegans* .	Agonism	Yes	Group 2	GenAge	Antagonist for cancer. PSMB5 is involved in cell cycle checkpoint during healthy aging.	PMID: 25395451, PMID:17392428
SMO	GPCR	Yes	Novel	12	11	43.5	3	Genomic instability, Impaired proteostasis, Stem cell exhaustion	17	12	5	2	7	3	4	Antagonism	Not reported	No evidence	NA	Yes	Group 4	-	Antagonist for cancer but SMO inhibition promotes aging. SMO has shown to be critical for the hedgehog signal transduction on the cell membrane. Hedgehog signaling is dysregulated in old hepatocytes, and this accelerates aging. Deleting SMO in young hepatocytes before partial hepatectomy prevented hedgehog pathway activation after partial hepatectomy and inhibited regeneration. In addition, hedgehog inhibition promoted telomere shortening and mitochondrial dysfunction in hepatocytes.	PMID: 34984806
MCM8	Hydrolase	No	Novel	13	10	23.8	3	Cellular senescence, Genomic instability, Impaired proteostasis	11	4	7	0	10	10	0	Antagonism	Not reported	No evidence	NA	Yes	Group 4	-	Antagonist for cancer. MCM8 is invovled in cell cycle checkpoint during healthy aging.	

PSMB2	Peptidase	Yes	Novel	14	10	23.9	1	Impaired proteostasis	26	0	26	1	10	10	0	Antagonism	Reported; Reduced lifespan only	Pro-Longevity: pbs-4 RNAi decreased lifespan by 30.92% in *C. elegans* .	NA	Yes	Group 3	SynergyAge	Antagonist for cancer. PSMB2 is invovled in cell cycle checkpoint during healthy aging.	PMID:17392428
PRIM1	Transferase	Yes	Novel	15	10	27.0	1	Genomic instability	21	2	19	0	9	9	0	Antagonism	Not reported	No evidence	NA	Yes	Group 4	-		
FGR	Tyrosine kinase	Yes	Novel	16	10	31.7	5	Cellular senescence, Deregulated nutrient signaling, Extracellular matrix stiffness, Inflammation, Mitochondrial dysfunction	18	10	8	2	7	3	4	Antagonism*	Not reported	No evidence	NA	Yes	Group 4		FGR inhibitor, such as dasatinib was investigated for cancer, although our data showed FGR downregulation in cancers. Dasatinib and quercetin restore α-Klotho in mice and humans, which may amplify protection against aging and age-related diseases.	PMID: 35292270
TOP2B	Isomerase	Yes	Novel	17	10	34.7	1	Epigenetic shift	24	0	24	0	5	4	1	Antagonism*	Not reported	No evidence	NA	Yes	Group 4	-	TOP2B inhibitor such as Etoposide approved for cancer, although our data showed TOP2B downregulation in cancer TOP2B is key decatenating enzyme that alters DNA topology by binding to two double-stranded DNA molecules.	
PSMA4	Peptidase	Yes	Novel	18	10	38.7	1	Impaired proteostasis	22	1	21	1	10	10	0	Antagonism	Not reported	No evidence	NA	Yes	Group 4	-	Antagonist for cancer. PSMA4 is invovled in cell cycle checkpoint during healthy aging.	
GNB1	Hydrolase	No	Novel	19	10	39.3	0	-	28	2	26	6	9	7	2	Antagonism	Not reported	No evidence	NA	No	-	-	Target did not associate with any aging hallmarks	
PSMA3	Peptidase	Yes	Novel	20	10	39.7	1	Impaired proteostasis	19	0	19	1	9	9	0	Antagonism	Reported; No effects on lifespan	Anit-Longevity: pas-7 RNAi slightly increased lifespan by 1.93% without significance in *C. elegans*.	NA	Yes	Group 4	SynergyAge	Antagonist for cancer. PSMA3 is involved in cell cycle checkpoint during healthy aging.	PMID:17392428
KDM2A	Oxidoreductase	No	Novel	21	10	45.9	3	Cellular senescence, Epigenetic shift, Genomic instability	12	1	11	0	8	7	1	Antagonism	Not reported	No evidence	NA	Yes	Group 4	-	Antagonist for cancer. KDM2A promotes stemness and angiogenesis of breast cancer. KDM2A deficiency in macrophages enhances thermogenesis to protect mice against HFD-induced obesity.	PMID: 27029061, PMID: 33462408
UBE2T	Acyltransferase	No	Novel	22	9	21.7	2	Cellular senescence, Impaired proteostasis	20	2	18	0	11	11	0	Antagonism	Not reported	No evidence	NA	Yes	Group 4	-	Antagonist for cancer. UBE2T is invovled in DNA repair (fanconi anemia pathway) during healthy aging.	
ITGB5	Receptor	Yes	Novel	23	9	24.2	2	Altered intercellular communications, Extracellular matrix stiffness	17	7	10	5	7	6	1	Antagonism	Reported; Reduced lifespan only	Pro-Longevity: Post-developmental RNAi of pat-3 shortens lifespan of wild type *C. elegans*.	NA	Yes	Group 3	GenAge	ITGB5 is a TGF-β activator. TGF-β signaling was shown to repress body size as well as lifespan *in vivo* . ITGB5 served as a ligand for Cyr61, a molecule stimulating the production of IL-6, which is considered an aging biomarker, via itgav/itgb5/Akt/NF-κB signaling pathway in rheumatoid arthritis.	PMID: 29070608, PMID: 17071038, PMID: 22547695, PMID: 23144747
WWP1	Acyltransferase	No	Novel	24	9	34.7	1	Impaired proteostasis	25	6	19	0	8	7	1	Antagonism	Reported; Lifespan extension	Pro-Longevity: wwp-1 overexpression increased lifespan by 20% in *C. elegans* . wwp-1 RNAi decreased lifespan by 9% in *C. elegans* . WWP1 homolog in *C. elegans* was found to increase life expectancy in response to dietary restriction	Agonism	Yes	Group 2	GenAge,SynergyAge	Antagonist for cancer. PTEN tumor suppressor for cancer treatment can be reactivated through inhibition of a MYC-WWP1 inhibitory pathway, unleashing tumor suppressive activity WWP1 negatively regulates osteoblast function by inhibiting osteoblast differentiation and migration. WWP1 contributes to shifts in matrix proteolytic profiles and a myocardial aging phenotype with diastolic heart. Inducing WWP1 expression caused LVH and preserved systolic function but impaired diastolic dysfunction, consistent with the phenotype of heart failure with a preserved ejection fraction.	PMID: 19553937, PMID: 18006689, PMID: 32822210, PMID: 31097636, PMID: 23553732
ITGA9	Receptor	No	Novel	25	9	46.7	2	Altered intercellular communications, Extracellular matrix [illegible]	24	3	21	4	8	2	6	Agonism	Not reported	No evidence	NA	Yes	Group 4	-	ITGA9 suppressed hepatocellular carcinoma metastasis.	PMID: 29951557
PPP5C	Esterase	No	Novel	26	9	48.2	5	Cellular senescence, Epigenetic shift, Genomic instability, Impaired proteostasis, Inflammation	22	1	21	2	7	7	0	Antagonism	Not reported	No evidence	NA	Yes	Group 4	-	Antagonist for cancer. PPP5C is invovled in DNA double-strand break repair during healthy aging.	
ANAPC2	Acyltransferase	No	Novel	27	9	54.6	1	Impaired proteostasis	12	1	11	0	8	5	3	Antagonism	Not reported	No evidence	NA	Yes	Group 4	-	Antagonist for cancerr. ANAPC2 is involved in cell cycle checkpoint during healthy aging. The anaphase promoting complex is required for memory function in mice	PMID: 21191042
MST1R	Receptor kinase	Yes	Novel	28	9	59.2	2	Inflammation, Telomere attrition	9	4	5	0	10	6	4	Antagonism	Not reported	No evidence	NA	Yes	Group 4	-	MST1R kinase accelerates pancreatic cancer progression via effects on both epithelial cells and macrophages. MST1R inhibitor prevents bone osteolysis.	PMID: 30967626, PMID: 28248933
KAT6A	Acyltransferase	No	Novel	29	9	59.9	3	Cellular senescence, Epigenetic shift, Genomic instability	13	4	9	0	6	4	2	Antagonism	Not reported	No evidence	NA	Yes	Group 4		KAT6A belongs to histone acetyltransferase (HAT). Stability of HATs and histone deacetylases activities is necessary to maintain normal cellular functions or otherwise leads to aging. KAT6A was found to regulate Nrf2/ARE signaling pathway and inhibit ROS accumulation in bone marrow-derived mesenchymal stem cells from the old, thus promoting proliferation, colony formation, and osteogenic differentiation of OBMSCs. KAT6A could promote osteogenesis of OBMSCs. However, for cancer, KAT6A inhibitor WM-1119 repressed the cell proliferation of sorafenib-resistant HCC cells, while overexpression of KAT6A could reverse the effect in the cells.	PMID: 19818845, PMID: 33541408, PMID: 34808502
UBE2M	Acyltransferase	No	Novel	30	9	60.1	2	Genomic instability, Impaired proteostasis	13	3	10	0	9	8	1	Antagonism	Not reported	No evidence	NA	Yes	Group 4	-	Antagonist for cancer. UBE2M is invovled in adaptive immune system during healthy aging.	
MAPKAPK	Protein kinase	Yes	Novel	31	8	35.0	4	Cellular senescence, Genomic instability, Stem cell exhaustion, Telomere attrition	22	1	21	2	11	11	0	Antagonism	Not reported	No evidence	NA	Yes	Group 4	-	Antagonsit for cancer. MAPKAPK5 is also considered as tumor suppressor serine/threonine-protein kinase involved in mTORC1 signaling.	
ADRA2C	GPCR	Yes	Novel	32	8	43.6	1	Altered intercellular communications	11	3	8	0	9	3	6	Agonism	Not reported	No evidence	NA	Yes	Group 4	-	Our data showed ADRA2C downregulation in multiple cancers. Dexmedetomidine, Clonidine, and Brimonidine, being ADRA2C agonists, were used for cancers in clincial trials. Guanfacine hydrochloride agonising ADRA2A, ADRA2B, and ADRA2C extended lifespan in *C. elegans*.	PMID: 32632241, PMID: 30201375, PMID: 24134630
ITK	Tyrosine kinase	Yes	Novel	33	8	45.1	2	Altered intercellular communications, Inflammation	15	13	2	2	6	3	3	Antagonism	Not reported	No evidence	NA	Yes	Group 4	-	ITK inhibitor was suggested for cancer treatment but our data showed ITK downregulation in cancers ITK is important for adaptive immune system during healthy aging. btk29a ficP null mutation decreased lifespan in *Drosophila* . Btk29A is the sole Tec family member in *Drosophila* , including itk.	PMID: 23672610, PMID: 16023106
BLK	Tyrosine kinase	Yes	Novel	34	8	48.0	6	Altered intercellular communications, Cellular senescence, Deregulated nutrient signaling, Epigenetic shift, Genomic instability, Inflammation	9	4	5	0	9	4	5	Antagonism*	Not reported	No evidence	NA	Yes	Group 4	-		
RBCK1	Acyltransferase	No	Novel	35	8	48.9	4	Epigenetic shift, Genomic instability, Impaired proteostasis, Inflammation	17	11	6	0	10	10	0	Antagonism	Not reported	No evidence	NA	Yes	Group 4	-	Antagonist for cancer. Knockdown RBCK1 reduced tumorigenicity in renal cancer *in vivo* . RBCK1 depletion increases p53 protein levels and p53 target genes, and RBCK1 interacts with PTEN (tumor suppressor geen) and promotes PTEN degradation in K48-linked ubiquitination.	PMID: 30874541, PMID: 35174471
PFKP	Unclassified kinase	No	Novel	36	8	50.5	0	-	17	4	13	2	9	8	1	Antagonism	Not reported	No evidence	NA	No	-	-	Target did not associate with any aging hallmarks	
RPS6KA4	AGC kinase	No	Novel	37	8	56.9	4	Epigenetic shift, Genomic instability, Impaired proteostasis, Inflammation	18	0	18	4	10	9	1	Antagonism	Not reported	No evidence	NA	Yes	Group 4	-	Antagonist for cancer. RPS6KA4 is a potentially valuable molecule for understanding HCC tumorigenesis. Increased RPS6KA4 might be a promising prognostic factor for low survival of hepatocellular carcinoma. Depletion of S6k1 resembles gene expression patterns of caloric restriction or prolongs lifespan by pharmacological activation of AMPK in mice.	PMID: 35232347, PMID: 19797661
ATAD2	Hydrolase	No	Novel	38	8	63.5	0	-	16	2	14	0	11	11	0	Antagonism	Not reported	No evidence	NA	No	-		Target did not associate with any aging hallmarks	
PSMB3	Peptidase	Yes	Novel	39	7	23.4	1	Impaired proteostasis	19	1	18	1	10	9	1	Antagonism	Reported; Reduced lifespan only	Pro-Longevity: pbs-3 RNAi decreased lifespan by 31.88% in *C. elegans*.	NA	Yes	Group 3	SynergyAge	Antagonist for cancer. PSMB3 is involved in cell cycle checkpoint during healthy aging.	PMID:17392428
PSMB6	Peptidase	Yes	Novel	40	7	33.4	1	Impaired proteostasis	21	0	21	1	8	4	4	Antagonism	Not reported	No evidence	NA	Yes	Group 4		Antagonist for cancer. PSMB6 is invovled in cell cycle checkpoint during healthy aging.	
PTPRF	Receptor phosphatas	No	Novel	41	7	33.4	1	Stem cell exhaustion	26	4	22	3	9	8	1	Antagonism	Not reported	No evidence	NA	Yes	Group 4	-	Our data showed PTPRF upregulation in mulitple cancers, suggesting antagonist for cancer treatment, but PTPRF as a novel tumor suppressor through deactivation of ERK1/2 signaling in gastric adenocarcinoma, and PPARγ inhibits breast cancer progression by upregulating PTPRF expression	PMID: 30464527, PMID: 31799666
PSMC3	Hydrolase	Yes	Novel	42	7	34.1	1	Impaired proteostasis	19	1	18	1	9	9	0	Antagonism	Reported; Reduced lifespan only	Pro-Longevity: rpt-5 RNAi decreased lifespan by 38.65% in *C. elegans* .	NA	Yes	Group 3	SynergyAge	Antagonist for cancer. PSMC3 is invovled for cell cycle checkpoint during healthy aging.	PMID:17392428

PRPF19	Acyltransferase	No	Novel	43	7	35.9	3	Cellular senescence, Genomic instability, Impaired proteostasis	27	1	26	0	10	9	1	Antagonism	Reported; Lifespan extension	Pro-Longevity: Ubiquitous overexpression of dPrp19 increased lifespan of female in *Drosophila* while inconsistent lifespan extention in male.	Agonism	Yes	Group 2	GenAge	Antagonist for cancer. PRPF19 is invovled for nucleotide excision repair during healthy aging.	PMID: 28649423
RPS6KA2	AGC kinase	Yes	Novel	44	7	37.7	3	Altered intercellular communications, Genomic instability, Impaired proteostasis	20	1	19	2	10	2	8	Antagonism*	Not reported	No evidence	NA	Yes	Group 4	-	Agonist for cancer. Re-expression of RPS6KA2 in ovarian cancer cell lines suppressed colony formation. In UCI101 cells, RPS6KA2 decreased proliferation, caused G1 arrest, and enhanced apoptosis. However, genetic as well as pharmacological inhibition of RPS6KA2 by the inhibitor BI-D1870 acted synergistically with erlotinib on tumor cell survival	PMID: 27028868, PMID: 16878154, PMID: 24403857
PSMC4	Hydrolase	Yes	Novel	45	7	43.4	1	Impaired proteostasis	18	0	18	1	10	10	0	Antagonism	Not reported	No evidence	NA	Yes	Group 4	-	Antagonist for cancer. PSMC4 is involved in cell cycle checkpoint during healthy aging.	
PSMC2	Hydrolase	Yes	Novel	46	7	44.7	1	Impaired proteostasis	21	1	20	1	8	8	0	Antagonism	Reported; Reduced lifespan only	Pro-Longevity: rpt-1 RNAi decreased lifespan by 36.23% in *C.elegans.*	NA	Yes	Group 3	GenAge,SynergyAge	Antagonist for cancer. PSMC2 is invovled in cell cycle checkpoint during healthy aging.	PMID:17392428
GANAB	Glycosylase	No	Novel	47	7	46.7	0	-	31	1	30	0	9	9	0	Antagonism	Not reported	No evidence	NA	No	-	-	Target did not associate with any aging hallmarks	
MAPKAPK	Protein kinase	No	Novel	48	7	46.9	1	Inflammation	26	4	22	4	9	6	3	Antagonism	Not reported	No evidence	NA	Yes	Group 4	-	Antagonist for cancer JNK1/2/3 are the upstream of MK3. While the role of MAPKAPK3/MK3 in aging is unknown, overexpression of MAPK8/JNK in roundworms also increases [illegible]	
IMPDH1	Oxidoreductase	Yes	Novel	49	7	47.4	0	-	15	3	12	0	10	9	1	Antagonism	Reported; No effects on lifespan	Anti-Longevity: Mycophenolic acid inhibiting IMPDH1 and IMPDH2 increased lifespan by 6.08% in *Drosophila* without significance. Mycophenolic acid inhibiting IMPDH1 and IMPDH2 slightly increased lifespan by 6.08% in *C. elegans* without significance.	Antagonism	No	-	DrugAge	Target did not associate with any aging hallmarks	PMID: 33008901
PPP4C	Esterase	No	Novel	50	7	48.7	4	Cellular senescence, Epigenetic shift, Genomic instability, Inflammation	25	4	21	1	9	9	0	Antagonism	Reported; reduced lifespan only	Pro-Longeviry: RNAi PP4-1 decreased lifespan in *Drosophila* .	NA	Yes	Group 3	-	Antagonist for cancer. PPP4C is involved in DNA double-strand break repair during healthy aging.	PMID: 34452932
RHOH	Hydrolase	No	Novel	51	7	50.4	3	Epigenetic shift, Genomic instability, Inflammation	10	6	4	5	7	5	2	Antagonism	Not reported	Not reported	NA	Yes	Group 4	-	Tumor suppressor gene	
PPP1CB	Esterase	No	Novel	52	7	53.9	0	-	15	5	10	4	7	2	5	Antagonism*	Not reported	No evidence	NA	No	-	-	Target did not associate with any aging hallmarks	
ADGRE5	GPCR	No	Novel	53	7	55.6	2	Altered intercellular communications, Inflammation	10	1	9	0	8	4	4	Antagonism	Not reported	No evidence	NA	Yes	Group 4	-	Antagonist for cancer. ADGRE5 is invovled in innate immune system during healthy aging.	
PSMA5	Peptidase	Yes	Novel	54	7	57.0	1	Impaired proteostasis	26	0	26	1	9	8	1	Antagonism	Reported; Reduced lifespan only	Pro-Longevity: pas-5 RNAi decreased lifespan by 34.78% in *C. elegans* .	NA	Yes	Group 3	-	Antagonist for cancer. PSMA5 is invovled in cell cycle checkpoint during healthy aging.	PMID:17392428
PIP5K1C	Non-protein kinase	No	Novel	55	7	57.6	3	Altered intercellular communications, Cellular senescence, Deregulated nutrient signaling	17	2	15	2	8	3	5	Antagonism*	Not reported	No evidence	NA	Yes	Group 4	-	Adipocyte-specific deletion of PIP5K1c reduces diet-induced obesity and insulin resistance by increasing energy expenditure Loss of Pip5k1c in mesenchymal stem cells leads to osteopenia by impairing bone remodeling	PMID: 34996482, PMID: 35090892
ITGB6	Receptor	Yes	Novel	56	7	61.7	4	Altered intercellular communications, Extracellular matrix stiffness, Genomic instability, Inflammation	11	3	8	3	6	4	2	Antagonism	Reported; reduced lifespan only	Pro-Longevity: Post-developmental RNAi of pat-3 shortens lifespan of wild type *C. elegans.*	NA	Yes	Group 3	GenAge	Antagonist for cancer. Our data showd ITGB6 upregulation in cancer. ITGB6 is a TGF-β activator. TGF-β signaling was shown to repress body size as well as lifespan *in vivo* .	PMID: 29070608, PMID: 23144747
S1PR1	GPCR	Yes	Novel	57	7	62.0	0	-	9	6	3	3	8	0	8	Antagonism*	Not reported	No evidence	NA	No	-	-	S1PR1 is highly expressed in several tumor types (based on literature). Targeting S1PR1 activity in endothelial cells (ECs) in the setting of anti-VEGFR2 therapy is a potentially attractive strategy to treat VEGFR2 refractory tumors. Target did not associate with any aging hallmarks	
FRK	Tyrosine kinase	Yes	Novel	58	7	64.6	1	Inflammation	19	1	18	0	10	8	2	Antagonism	Not reported	No evidence	NA	Yes	Group 4	-	Antagonist for cancer. FRK is invovled in innate immune system during healthy aging.	
CDC34	Acyltransferase	No	Novel	59	7	69.7	2	Genomic instability, Impaired proteostasis	14	9	5	0	9	6	3	Antagonism	Not reported	No evidence	NA	Yes	Group 4	-	Antagonist for cancer. CDC34 is involved in adaptive immune system during healthy aging.	
CSF2RB	Immunoglobulin	Yes	Novel	60	6	37.3	1	Inflammation	12	3	9	3	6	2	4	Antagonism*	Not reported	Not reported	NA	Yes	Group 4	-	Upregulation of CSF2RB was found in 3 cancers. Upregulation of CSF2RB may increase the production of Interleukin-3, Interleukin-5 and GM-CSF, leading to inflammation	
VRK1	Protein kinase	No	Novel	61	6	37.5	1	Cellular senescence	19	1	18	0	10	10	0	Antagonism	Reported; Lifespan extension	Pro-Longevity: VRK1 RNAi decreased lifespan by 36% in *C. elegans* . VRK1 extended lifespan by activation of AMPK via phosphorylation in *C. elegans.*	Agonism	Yes	Group 2	Age, SynergyAge,pape	Antagonist for cancer.	PMID: 18006689, PMID: 32937443
HLTF	Acyltransferase	No	Novel	62	6	41.7	3	Cellular senescence, Epigenetic shift, Impaired proteostasis	20	5	15	0	9	9	0	Antagonism	Not reported	No evidence	NA	Yes	Group 4	-	Antagonist for cancer, but loss of HLTF function promotes intestinal carcinogenesis	PMID: 22452792
STK26	Protein kinase	No	Novel	63	6	44.7	1	Genomic instability	20	0	20	0	10	7	3	Antagonism	Not reported	No evidence	NA	Yes	Group 4	-	Antagonist for cancer, but depletion of STK24 (or MST4) in mice promoted gastric tumorigenesis.	PMID: 32271880
PI4KA	Non-protein kinase	No	Novel	64	6	46.3	0	-	20	1	19	0	6	2	4	Antagonism*	Not reported	No evidence	NA	No	-	-	Overexpression of PI4KA is associated with undifferentiated status and poor prognosis of human hepatocellular carcinoma. Target did not associate with any aging hallmarks	PMID: 24393405
PSMC5	Hydrolase	Yes	Novel	65	6	46.3	1	Impaired proteostasis	19	3	16	1	8	7	1	Antagonism	Reported; Reduced lifespan only	Pro-Longevity: rpt-6 RNAi decreased lifespan by 38.16% in *C. elegans* .	NA	Yes	Group 3	SynergyAge	Antagonist for cacer. PSMC5 is involved in cell cycle checkpoint during healthy aging	PMID:17392428
CPSF3	Esterase	No	Novel	66	6	46.7	0	-	21	0	21	0	9	9	0	Antagonism	Not reported	No evidence	NA	No	-	-	Target did not associate with any aging hallmarks	
RPS6KB2	AGC kinase	Yes	Novel	67	6	47.3	2	Impaired proteostasis, Telomere attrition	12	1	11	7	6	5	1	Antagonism	Reported; Lifespan extension	Anti-Longevity: Dominant-negative mutant increased lifespan by 22% in *Drosophila.* Constitutively active mutant decreased lifespan by 34% in *Drosophila* . RNA interference reducing S6K (rsks-1) mRNA levels by about two-fold extended mean lifespan 13-47% and maximum lifespan by up to 60% in *C. elegans* . Deletion mutants also lived longer. RNAi resulted in a 22% maximum lifespan increase in *C. elegans* . In the rsks-1 (ok1255) mutant background, inhibition of ifg-1 led to a further extension of 40% in maximum lifespan.	Antagonism	Yes	Group 1	GenAge	Antagonist for cancer. Drug target of MSC-2363318A and LY-2780301 investigated in clincial trial (NCT01971515, NCT02018874 and NCT01115751) for cancer treatment.	PMID: 15186745, PMID: 17266679, PMID: 17266680
CTSV	Peptidase	No	Novel	68	6	50.0	3	Extracellular matrix stiffness, Impaired proteostasis, Inflammation	12	2	10	0	10	9	1	Antagonism	Not reported	No evidence	NA	Yes	Group 4		Antagonist for cancer - CTSV promotes bladder cancer progression, and our data showed its upregulation in cancer. CTSV is involved in innate immune system, adaptive immune system and collagen formation for healthy aging.	PMID: 35443863
KSR1	Tyrosine kinase-like	No	Novel	69	6	50.2	1	Impaired proteostasis	14	7	7	3	6	1	5	Antagonism*	Not reported	No evidence	NA	Yes	Group 4	-	Antagonist for cancer. Evidences suggest antagonist for cancer treatment: (1) tumor-bearing KSR1 deficiency mice showed significant improvement in all-cause morbidity, and (2) the growth of MT-driven mammary tumor was moderately slowed in ksr1-/- mice, although our data showed KSR1 downregulation in cancer . KSP1 is positively regulate MAPK signaling in the context of constitutively active RAS. RasV12 failed to induce p53, p19ARF, p16INK4a, and p15INK4b expression in KSR1−/− primary mouse embryo fibroblasts and increased proliferation instead of causing growth arrest. Reintroduction of wild-type KSR1 rescued RasV12-induced senescence.	PMID: 29596465, PMID: 12874031, PMID: 16507997
UBE2L6	Acyltransferase	No	Novel	70	6	52.8	1	Impaired proteostasis	10	8	2	0	8	6	2	Antagonism	Not reported	No evidence	NA	Yes	Group 4	-	Antagonist for cancer. UBE2L6 is involved in adaptive immune system and DNA repair during healthy aging	
PSMB4	Peptidase	Yes	Novel	71	6	54.0	3	Impaired proteostasis, Inflammation, Mitochondrial dysfunction	19	1	18	1	8	8	0	Antagonism	Reported; Reduced lifespan only	Pro-Longevity: pbs-7 RNAi decreased lifespan by 34.78% in *C. elegans* .	NA	Yes	Group 3	SynergyAge	Antagonist for cacer but PSMB4 is important for cell cycle checkpoint during healthy aging	PMID: 17392428
PSMA1	Peptidase	Yes	Novel	72	6	55.3	2	Impaired proteostasis, Inflammation	22	0	22	1	9	9	0	Antagonism	Reported; Reduced lifespan only	Pro-Longevity: pas-6 RNAi decreased lifespan by 31.88% in *C. elegans* .	NA	Yes	Group 3	SynergyAge	Antagonist for cacer. PSMA1 is involved in cell cycle checkpoint during healthy aging.	PMID:17392428

DDX39A	Hydrolase	No	Novel	73	6	58.8	0	-	12	8	4	0	11	11	0	Antagonism	Reported; Reduced lifespan only	Pro-Longevity: Hel-1 downregulation resulted in a 9.3% decrease in lifespan compared to wild type in *C. elegans* . This effect was even more pronounced in daf-2 mutants which showed a 26.9% decrease in lifespan. The results suggested that hel-1 promoted longevity	NA	No	-	GenAge	Target did not associate with any aging hallmarks	PMID: 26195740
BAZ1B	Tyrosine kinase	No	Novel	74	6	64.0	2	Cellular senescence, Epigenetic shift	24	3	21	0	9	9	0	Antagonism	Not reported	No evidence	NA	Yes	Group 4	-	Antagonist for cancer. BAZ1B is invovled in DNA double-strand break repair during healthy aging.	
TTK	Protein kinase	No	Novel	75	6	70.3	1	Genomic instability	20	1	19	0	11	11	0	Antagonism	Not reported	No evidence	NA	Yes	Group 4	-		
CANT1	Hydrolase	No	Novel	76	6	71.0	3	Epigenetic shift, Genomic instability, Inflammation	26	0	26	0	10	8	2	Antagonism	Reported; Reduced lifespan only	Pro-Longevity: apy-1 RNAi decreased lifespan in *C. elegans* .	NA	Yes	Group 3	GenAge	Antagonist for cancer. CANT1 is invovled in innate immune system during healthy aging.	PMID: 18216284
TRAF7	Acyltransferase	No	Novel	77	6	72.7	3	Altered intercellular communications, Genomic instability, Impaired proteostasis	17	0	17	1	10	10	0	Antagonism	Not reported	No evidence	NA	Yes	Group 4	-	Tumor suppressor gene	
HIPK1	CMGC kinase	No	Novel	78	5	28.8	3	Altered intercellular communications, Cellular senescence, Genomic instability	18	1	17	0	8	2	6	Antagonism*	Reported; Reduced lifespan only	Pro-Longevity: hpk-1 RNAi decreased lifespan by 35% in *C. elegans* .	NA	Yes	Group 3	GenAge	Our data showed downregulation in cancer, suggesting agonist for cancer treatment. However, oncogenically transformed HIPK1 −/− mouse embryonic fibroblasts were more susceptible than transformed HIPK1 +/+ cells to apoptosis induced by DNA damage, and carcinogen-treated HIPK1 −/− mice developed fewer and smaller skin tumors than HIPK1 mice. HIPK1 drives p53 activation to limit colorectal cancer cell growth	PMID: 23676219, PMID: 12702766, PMID: 18006689
PSMA2	Peptidase	Yes	Novel	79	5	34.0	1	Impaired proteostasis	20	0	20	1	10	8	2	Antagonism	Not reported	No evidence	NA	Yes	Group 4	-	Antagonist for cacer. PSMA2 is invoved in cell cycle checkpoint during healthy aging	
PPP2CB	Esterase	Yes	Novel	80	5	36.4	6	Altered intercellular communications, Epigenetic shift, Genomic instability, Impaired proteostasis, Inflammation, Mitochondrial dysfunction	17	3	14	6	9	1	8	Antagonism*	Reported; reduced lifespan only	Pro-Longevity: let 92 RNAi decreased lifespan in *C. elegans* .	NA	Yes	Group 3	-	Antagonist for cancer. LB-100, PPP2CB inhibitor, was investigated for the treatment of glioblastoma and oligodendroglioma (phase II). LB-100 targets PPP2CB, PPP5C and PPP2CA. LB-100 exhibits anti-cancer activity via its chemo- and radio-sensitizing properties. However, our data showed downregulation in PPP2CB in cancer was inconsistent with the evidence that inhiiting PPP2CB exhibits ati-cancer activities Besides, PPP2CB is important for cell cycle checkpoint and adaptive immune system during healthy aging.	PMID: 25897893, PMID: 33315870
GAK	Protein kinase	No	Novel	81	5	38.2	1	Impaired proteostasis	12	9	3	0	9	6	3	Antagonism	Reported; reduced lifespan only	Pro-Longevity: aux RNAi decreased lifespan in *Drosophila* .	NA	Yes	Group 3	-	Antagonist for cancer. GAK knockdown by siRNA decreased cell proliferation in osteosarcoma. GAK ablation in mice causes kidney failure due to calpain activation	PMID: 20881269, PMID: 33208557, PMID: 28147270
TEC	Tyrosine kinase	Yes	Novel	82	5	40.8	3	Extracellular matrix stiffness, Inflammation, Stem cell exhaustion	8	5	3	1	9	5	4	Antagonism*	Reported; Reduced lifespan only	Pro-Longevity: btk29a ficP null mutation decreased lifespan in *Drosophila.* Btk29A is the sole Tec family member in *Drosophila.*	NA	Yes	Group 3	-	TEC kinase stabilized PLK4 to promote liver cancer metastasis.	PMID: 16023106, PMID: 34637843
UBA2	Acyltransferase	No	Novel	83	5	44.8	1	Impaired proteostasis	18	2	16	0	8	8	0	Antagonism	Not reported	No evidence	NA	Yes	Group 4	-	UBA2 is a heterodimer, acting as an E1 ligase for SUMO1, SUMO2, SUMO3, and probably SUMO4. Antagonist for cancer. UBA2 could promote cell migration and invasion through Wnt/β-catenin signaling in gastric cancer. UBA2 knockdown and inhibiting SAE1/UBA2-mediated SUMOylation resulted in reduced glycolysis. SUMOylation of IkBa has as a strong inhibitory effect on NF-κB-dependent transcription, and SUMOylation of p53 prevents its proteasome degradation.	PMID: 30479464, PMID: 28088449, PMID: 31788344, PMID: 32938830
RAB1B	Hydrolase	No	Novel	84	5	53.4	2	Impaired proteostasis, Mitochondrial dysfunction	24	0	24	0	8	5	3	Antagonism	Not reported	No evidence	NA	Yes	Group 4	-	RAB1B is invovled in cell cycle regulation during healthy aging.	
PLXNA2	Receptor	No	Novel	85	5	58.6	0	-	12	2	10	0	8	2	6	Antagonism*	Not reported	No evidence	NA	No	-	-	Target did not associate with any aging hallmarks	
PSMB7	Peptidase	Yes	Novel	86	5	63.0	1	Impaired proteostasis	18	1	17	1	7	7	0	Antagonism	Reported; Reduced lifespan only	Pro-Longevity: pbs-2 RNAi decreased lifespan by 31.88% in *C. elegans* .	NA	Yes	Group 3	SynergyAge	Antagonist for cancer. PSMB7 is involved in cell cycle checkpoint during healthy aging	PMID: 17392428
KMT2B	Methyltransferase	No	Novel	87	5	65.2	0	-	15	2	13	0	8	7	1	Antagonism	Not reported	No evidence	NA	No	-	-	Target did not associate with any aging hallmarks	
TRIM37	Acyltransferase	No	Novel	88	5	76.8	5	Altered intercellular communications, Epigenetic shift, Genomic instability, Impaired proteostasis, Inflammation	29	1	28	0	8	8	0	Antagonism	Not reported	No evidence	NA	Yes	Group 4	-	Antagonist for cancer. TRIM37 is involved in adaptive immune system during healthy aging.	
MTHFD2	Oxidoreductase	No	Novel	89	4	23.3	3	Deregulated nutrient signaling, Epigenetic shift, Mitochondrial dysfunction	9	1	8	0	8	8	0	Antagonism	Reported; Lifespan extension	Pro-Longevity: Ubiquitous Nmdmc overexpression increased lifespan by 220% in *Drosophila* . Nmdmc fat body specific overexpression increased lifespan by 23% in male *Drosophila* and 14% in female *Drosophila* .	Agonism	Yes	Group 2	GenAge	Antagonist for cancer. MTHFD2 is involved in metabolism of water-soluble vitamins and cofactors during healthy aging	PMID: 26319556
TAOK1	Protein kinase	No	Novel	90	4	41.3	2	Cellular senescence, Genomic instability	23	2	21	2	6	5	1	Antagonism	Not reported	No evidence	NA	Yes	Group 4	-	Antagonist for cancer. TAOK1 is invovled for cell cycle checkpoints during healthy aging	
ARL2	Hydrolase	No	Novel	91	4	49.5	1	Mitochondrial dysfunction	13	4	9	0	9	4	5	Antagonism	Not reported	No evidence	NA	Yes	Group 4	-	Antagonism or agonisim depends on cancer types. Inconsistent dysregulation (upregulate and downregulate) in different cancers. ARL2 inhibits the proliferation, migration and tumorigenicity of glioma cells. ARL2 was first reported to behave as a tumor suppressor in breast cancer. In contrast, miR-214 down-regulates ARL2 and suppresses growth and invasion of cervical cancer cells. ARL2 is required for homologous recombination repair in colon cancer stem cells.	PMID: 29843637, PMID: 32426352, PMID: 28137590, PMID: 35567502
HELZ2	Hydrolase	No	Novel	92	4	50.5	0	-	12	2	10	0	9	9	0	Antagonism	Not reported	No evidence	NA	No	-		Target did not associate with any aging hallmarks	
PIP5K1A	Non-protein kinase	No	Novel	93	4	50.8	3	Cellular senescence, Deregulated nutrient signaling, Extracellular matrix stiffness	9	3	6	1	9	9	0	Antagonism	Not reported	No evidence	NA	Yes	Group 4	-		
CLOCK	Acyltransferase	No	Novel	94	4	53.0	7	Altered intercellular communications, Cellular senescence, Deregulated nutrient signaling, Epigenetic shift, Genomic instability, Impaired proteostasis, Inflammation	31	3	28	0	5	3	2	Antagonism*	Reported; Reduced lifespan only	Pro-Longevity: The average lifespan of Clock-/- mice is reduced by 15% compared with wild type mice, while maximum lifespan is reduced by more than 20%.	NA	Yes	Group 3	GenAge	Age-related changes of clock gene expression promote declining autophagy levels	PMID: 21149897, PMID: 27574892, PMID: 21566258
PSMC1	Hydrolase	Yes	Novel	95	4	55.0	1	Impaired proteostasis	30	0	30	0	10	9	1	Antagonism	Not reported	No evidence	NA	Yes	Group 4	-	Antagonist for cacer. PSMC1 is invovled in cell cycle checkpoint during healthy aging	
USP11	Peptidase	No	Novel	96	4	56.3	1	Impaired proteostasis	14	9	5	0	11	6	5	Antagonism	Not reported	No evidence	NA	Yes	Group 4	-	Antagonist for cancer. USP11 is involved in regulating p53 stability by deubiquitinating p53.	PMID: 25471832
PSMB1	Peptidase	Yes	Novel	97	4	56.8	1	Impaired proteostasis	12	1	11	1	6	6	0	Antagonism	Reported; No effects on lifespan	No evidence on modulating longevity: pbs-6 RNAi did not change lifespan in *C. elegans* .	NA	Yes	Group 4	SynergyAge	Antagonist for cacer. PSMB1 is involved in cell cycle checkpoint during healthy aging	PMID: 17392428
GALNT2	Glycosyltransferase	No	Novel	98	4	57.0	0	-	20	0	20	0	8	7	1	Antagonism	Not reported	No evidence	NA	No	-	-	Target did not associate with any aging hallmarks	
RPS6KA6	AGC kinase	Yes	Novel	99	4	57.5	4	Cellular senescence, Genomic instability, Impaired proteostasis, Mitochondrial dysfunction	19	5	14	2	8	2	6	Antagonism*	Not reported	No evidence	NA	Yes	Group 4	-	p90RSK inhibitor, TAS0612, was investigated for neoplasm in phase I trial, but our data showed downregulation of RPS6KA6 in cancers. Depletion of S6k1 resembles gene expression patterns of caloric restriction or prolongs lifespan by pharmacological activation of AMPK in mice. S6K1 regulates glucose metabolism via feedback regulation of insulin receptor substrate 1 to improve glucose tolerance and insulin sensitivity in liver-specific and systematic S6k1-deficient mice.	PMID: 19797661, PMID: 22493495, PMID: 15306821
HERC5	Acyltransferase	No	Novel	100	4	59.3	2	Impaired proteostasis, Inflammation	7	3	4	0	8	2	6	Antagonism*	Not reported	No evidence	NA	Yes	Group 4	-	Knockdown of HERC5 significantly induced the expression of p53, p21 and Bax/Bcl-2 in HCC cells, resulting in apoptosis augment. HERC5 was over-expressed in both HCC tissue samples and cell	PMID: 28919514

[1] Two novelty settings (high-confidence setting and novel setting) were applied for target identification
[2] Top 100 AI-derived targets for each cancer were ranked by PandaOmics, yielding a list of 301 high-confidence and 319 novel targets (ranked as topm 100 in at least 1 cancer) across 11 cancers. Based on the occurrence and average ranking, the overall top-100 AI-derived common high-confidence and novel cancer targets were identified, yielding a list of 200 common cancer targets.
[3] The association of targets with 12 aging hallmarks were based on Gene Ontology terms
[4] The association of targets with 10 cancer hallmarks were based on CHG database and COSMIC.
[5] Targeting the gene that can increase the susceptibility to cancer or metastasis was marked with asterisk. The effects of IGF1, and ATM on longevity was inconclusive (i.e. inconsistent evidence showed their anti-longevity and pro-longevity activities). The effect of target on longevity upon drug treatment is considered given drug targets ≤2 genes.
[6] Generally, upregulation in expression leads to proposed antagonist for cancer; downregulation in expression leads to proposed agonist for cancer. * The therapeutic approach is proposed based on target's mechanism of action
[7] Target wheel is shown in Figure 4

Table S7. Potential dual-purpose candidates for delaying aging and anti-cancer treatment

Group-4 targets[1]	Clinical trial status	Novelty	Occurence in any overlapped biological processes[2]	Therapeutic approach for cancer treatment[3]	Predicted therapeutic approach for healthy anti-aging	Potential dual-purpose candidatess	Role in healthy aging	References
ADRA2C	Yes	Novel	✓	Agonism	Agonism	✓	Guanfacine hydrochloride agonisting ADRA2A, ADRA2B and ADRA2C extended lifepan in *C. elegans* .	PMID: 24134630
AKT3	Yes	HC	✓	Antagonism*	Antagonism	✓	Aging-induced Akt activation involves in aging-related pathologies and Aβ-induced toxicity	PMID: 31183966
CSF2RB	Yes	Novel	✓	Antagonism*	Antagonism	✓	Upregulation of CSF2RB may increase the production of Interleukin-3, Interleukin-5 and GM-CSF, leading to inflammation	-
FASN	Yes	HC	✓	Antagonism	Antagonism	✓	FANS inhibitor prevented the initiation of senescence induction in hematopoietic stem cells and reduced the effect of the activation of senescence on different age-associated diseases.<br>DEHP/DEP (increasing fasn-1 expression, and affecting other lipid metabolic genes) treated C. elegans lifespan decreased.	PMID: 30962418,<br>PMID: 29020644
FGR	Yes	Novel	✓	Antagonism*	Antagonism	✓	FGR inhibitor, such as dasatinib was investigated for cancer, although our data showed FGR downregulation in cancers.<br>Dasatinib and quercetin restore α-Klotho in mice and humans, which may amplify protection against aging and age-related diseases.	PMID: 35292270
HCK	Yes	Novel	✓	Antagonism	Antagonism	✓	Constitutive activation of the SRC family kinase Hck results in spontaneous pulmonary inflammation and an enhanced innate immune response.	PMID: 12208875
HDAC2	Yes	HC	✓	Antagonism	Antagonism	✓	Inactivating histone deacetylase HDA promotes longevity by mobilizing trehalose metabolism.	PMID: 33790287
HMGB1	Yes	HC	✓	Antagonism	Antagonism	✓	HMGB1 mediates neuroinflammatory priming in the aged brain.<br>Blocking the actions of HMGB1 appears to "desensitize" aged microglia to an immune challenge,<br>thereby preventing exaggerated behavioral and neuroinflammatory responses following infection	PMID: 30988279,<br>PMID: 27466339
IL1B	Yes	HC	✓	Antagonism	Antagonism	✓	IL-1α and IL-1β signal upregulate the senescence-associated secretory phenotype in a cooperative manner.<br>IL-1β promotes the age-associated decline of beta cell function	PMID: 30988157,<br>PMID: 34746709
ITGAV	Yes	HC	✓	Antagonism	Antagonism	✓	ITGB5 served as a ligand for Cyr61, a molecule stimulating the production of IL-6, which is<br>considered an aging biomarker, via itgav/itgb5/Akt/NF-κB signaling pathway in rheumatoid arthritis	PMID: 32064162,<br>PMID: 17071038,<br>PMID: 22547695
KDM1A	Yes	HC	✓	Antagonism	Antagonism	✓	KDM1A, is a drug target of IMG-7289, lysine-specific demethylase 1 inhibitor, investigated for the treatment of leukemia in trial (NCT02842827).<br>Modulation of KDM1A with vafidemstat (KDM1A inhibitor) rescues memory deficit and behavioral alterations in mice	PMID: 32469975
KDM2A	No	Novel	✓	Antagonism	Antagonism	✓	KDM2A deficiency in macrophages enhances thermogenesis to protect mice against HFD-induced obesity.	PMID: 27029061,<br>PMID: 33462408
KRAS	Yes	HC	✓	Antagonism	Antagonism	✓	Genetic inhibition of Ras was found to extend lifespan.<br>Adult-onset administration of the drug trametinib inhibiting MAP2K1 and MAP2K2, a highly specific inhibitor of Ras-Erk-ETS signaling, extended lifespan in *Drosophila*.	PMID: 29276789,<br>PMID: 26119340
LYN	Yes	Novel	✓	Antagonism	Antagonism	✓	Lyn suppresses osteoclastogenesis in vitro and in vivo.	PMID: 19171907
MET	Yes	HC	✓	Antagonism	Antagonism	✓	MET activates RAS and AKT signaling.	PMID: 28677234
MMP2	Yes	HC	✓	Antagonism	Antagonism	✓	Aging is associated with increased matrix metalloproteinase-2 activity in the human aorta<br>MMP2 was considered as classsical senescence-associated secretory phenotype (SASP).	PMID: 15831360
MST1R	Yes	Novel	✓	Antagonism	Antagonism	✓	MST1R inhbito prevents bone osteolysis.	PMID: 30967626,<br>PMID: 28248933
RARA	Yes	HC	✓	Agonism	Agonism	✓	RAR agonist ameliorated the UV-induced damage to skin collagen fibers, and increased the collagen content in photoaged skin through RAR.	PMID: 28849147
RARB	Yes	HC	✓	Agonism	Agonism	✓	RAR agonist ameliorated the UV-induced damage to skin collagen fibers, and increased the collagen content in photoaged skin through RAR.	PMID: 28849147
RPS6KA2	Yes	Novel	✓	Antagonism*	Antagonism	✓	Involved PI3K/AKT/MTOR signaling	-
RPS6KA4	No	Novel	✓	Antagonism	Antagonism	✓	Depletion of S6k1 resembles gene expression patterns of caloric restriction or prolongs lifespan by pharmacological activation of AMPK in mice.	PMID: 35232347,<br>PMID: 19797661
RPS6KA6	Yes	Novel	✓	Antagonism*	Antagonism	✓	Depletion of S6k1 resembles gene expression patterns of caloric restriction or prolongs lifespan by pharmacological activation of AMPK in mice.<br>S6K1 regulates glucose metabolism via feedback regulation of insulin receptor substrate 1 to improve glucose tolerance and insulin sensitivity in liver-specific and systematic S6k1-deficient mice.	PMID: 19797661,<br>PMID: 22493495,<br>PMID: 15306821
SRC	Yes	HC	✓	Antagonism	Antagonism	✓	Dasatinib targeting SRC was considered as senolytic used to remove senescent cell.	-
ABL1	Yes	HC	✓	Antagonism	Not suitable for antagonsim	x	ABL1 is important for DNA double-strand break repair during healthy aging.<br>Senolytic treatment reduces cell senescence. Quercetin, senolytic drug, inhibits multiple kinases including ABL1.<br>Imatinib mesylate inhibiting ABL1 reduced lifespan in *C. elegans*.	PMID: 3201088
ADGRE5	No	Novel	✓	Antagonism	Not suitable for antagonsim	x	ADGRE5 is invovled in innate immune system during healthy aging.	Reactome
ANAPC2	No	Novel	✓	Antagonism	Not suitable for antagonsim	x	ANAPC2 is involved in cell cycle checkpoint during healthy aging.<br>The anaphase promoting complex is required for memory function in mice	PMID: 21191042
AURKA	Yes	HC	✓	Antagonism	Not suitable for antagonsim	x	AURKA is involved in cell cycle regulation during healthy aging	Reactome
AURKB	Yes	HC	✓	Antagonism	Not suitable for antagonsim	x	AURKA is involved in cell cycle checkpoing during healthy aging	Reactome
BAZ1B	No	Novel	✓	Antagonism	Not suitable for antagonsim	x	BAZ1B is invovled in DNA double-strand break repair during healthy aging.	Reactome
BLK	Yes	Novel	✓	Antagonism*	Conditional	x	BLK is a pro-senescence kinase.	PMID: 26583757
CDC34	No	Novel	✓	Antagonism	Not suitable for antagonsim	x	CDC34 is involved in adaptive immune system during healthy aging.	Reactome
CDK6	Yes	HC	✓	Antagonism	Not suitable for antagonsim	x	CDK6 is involved in cell cycle regulation during healthy aging	Reactome
CXCR4	Yes	HC	✓	Antagonism	Conditional<br>(Agonism for young,<br>Antagonism for old)	x	CXCR4 gene deletion in young mesenchymal stem cells accelerates an aging phenotype including increased production of reactive oxygen species, DNA damage, senescence, and reduced proliferation.<br>In constrast, CXCL12/CXCR4 promotes inflammation. Targeting CXCR4 for antiaging may be suitable for aged adults only.	PMID: 26848769,<br>PMID: 32418119

DNM2	No	HC	✓	Antagonism	Not suitable for antagonsim	x	Tumor suppressor gene	-
DNMT1	Yes	HC	✓	Antagonism	Agonism	x	DNMT1 maintains genomic methylation stabiluty and insufficient DNA methylation affects healthy aging and promotes age-related health problems<br>No difference in longevity was observed between Dnmt1-deficient mice and normal controls.	PMID: 22704347,<br>PMID: 16510855
DNMT3A	Yes	HC	✓	Antagonism	Agonism	x	Constitutive loss of DNMT3A causes morbid obesity through misregulation of adipogenesis	PMID: 35635747
DNMT3B	No	HC	✓	Antagonism	Agonism	x	DNMT3B plays a protective role against hepatocarcinogenesis caused by chronic inflammation via maintaining mitochondrial homeostasis.	PMID: 33221743,<br>PMID: 33277576
FES	No	Novel	✓	Antagonism*	Not suitable for antagonsim	x	FES kinase activity is also dispensable for hematopoiesis.	PMID:<br>322556211,<br>PMID: 10523632
FGF2	Yes	HC	✓	Antagonism*	Not suitable for antagonsim	x	FGF2 (or bFGF) is neuroprotective for healthy aging.<br>It can improve motor function recovery, increase tyrosine hydroxylase positive neuron survival,<br>and upregulate the levels of neurotransmitters in the brain of a rat model of Parkinson’s disease.	PMID: 30274251
FGFR1	Yes	HC	✓	Antagonism*	Not suitable for antagonsim	x	Mice with an attenuation of FGFR1 signalling develop diabetes with age and exhibit a decreased number of beta-cells,<br>and lower levels of FGFR1 in mice have also been related to craniofacial defects.	PMID: 12514106,<br>PMID: 11130726
FGFR2	Yes	HC	✓	Antagonism*	Not suitable for antagonsim	x	FGFR2 is important for cell division, cell maturation, formation of new blood vessels, wound healing, and bone growth and development	-
FRK	Yes	Novel	✓	Antagonism	Not suitable for antagonsim	x	FRK is invovled in innate immune system during healthy aging.	Reactome
HLTF	No	Novel	✓	Antagonism	Not suitable for antagonsim	x	Loss of HLTF function promotes intestinal carcinogenesis	PMID: 22452792
ITK	Yes	Novel	✓	Antagonism	Not suitable for antagonsim	x	ITK is important for adaptive immune system during healthy aging.<br>btk29a ficP null mutation decreased lifespan in *Drosophila* .<br>Btk29A is the sole Tec family member in *Drosophila* , including itk.	PMID: 23672610,<br>PMID: 16023106
KAT6A	No	Novel	✓	Antagonism	Agonism	x	KAT6A belongs to histone acetyltransferase (HAT). Stability of HATs and histone deacetylases activities is necessary to maintain normal cellular functions or otherwise leads to aging.<br>KAT6A was found to regulate Nrf2/ARE signaling pathway and inhibit ROS accumulation in bone marrow-derived mesenchymal stem cells from the old,<br>thus promoting proliferation, colony formation, and osteogenic differentiation of OBMSCs. KAT6A could promote osteogenesis of OBMSCs.	PMID: 19818845,<br>PMID: 33541408,<br>PMID: 34808502
KDR	Yes	HC	✓	Antagonism*	Not suitable for antagonsim	x	KDR antagonists such as Ramucirumab, Cabozantinib approved for cancer treatment but our data showed KDR downregulation in cancers.<br>KDR may be needed for normal angiogenesis, to ensure developing or healing tissues receive an adequate supply of nutrients.	-
KIT	Yes	HC	✓	Antagonism*	Agonism	x	Adult myocardium relies on c-kit expression to regenerate after injury and to counteract aging effects on cardiac structure and function.<br>Imatinib mesylate inhibiting PDGFRB, ABL1, KIT, BCR reduced lifespan in *C. elegans.*	PMID: 31275242,<br>PMID: 32010883
KSR1	No	Novel	✓	Antagonism*	Agonsim	x	KSP1 is positively regulate MAPK signaling in the context of constitutively active RAS. Genetic inhibition of Ras was found to extend lifespan.<br>RasV12 failed to induce p53, p19ARF, p16INK4a, and p15INK4b expression in KSR1−/− primary mouse embryo fibroblasts and increased proliferation instead of causing growth arrest.<br>Reintroduction of wild-type KSR1 rescued RasV12-induced senescence.	PMID: 29596465,<br>PMID: 12874031,<br>PMID: 16507997
MAPKAPK3	No	Novel	✓	Antagonism	Agonism	x	JNK1/2/3 are the upstream of MK3.<br>While the role of MAPKAPK3/MK3 in aging is unknown, overexpression of MAPK8/JNK in roundworms also increases lifespan.	-
MAPKAPK5	Yes	Novel	✓	Antagonism	Not suitable for antagonsim	x	MAPKAPK5 is considered as tumor suppressor serine/threonine-protein kinase involved in mTORC1 signaling.	-
MCM8	No	Novel	✓	Antagonism	Not suitable for antagonsim	x	MCM8 is involved in cell cycle checkpoint and DNA replication during healthy aging.	Reactome
MDM2	Yes	HC	✓	Antagonism	Not suitable for antagonsim	x	MDM2 is a negative regulator of p53, a tumor-suppressor gene. Dominant-negative versions of *Drosophila* melanogaster p53 in adult neurons extends lifespan.<br>Taken together, antagonizing MDM2 may result in increased p53 activities, which in turn contribute to accelerated aging.<br>However, small-molecule MDM2 antagonists attenuate the senescence-associated secretory phenotype.	-
NCOA1	No	HC	✓	Antagonism*	Agonism	x	Aging or ER antagonists were shown to downregulate NCOA1 in the hippocampus of female mice.<br>17β-estradiol treatment can upregulate NCOA1. Beta-Estradiol was shown to increase lifespan in *C. elegans* .<br>Taken together, NOCA1 agonist may delay aging.<br>NCOA1 is required for the anti-obesogenic effects of 17β-estradiol.	PMID: 26287601,<br>PMID: 24134630
NCOA3	No	HC	✓	Antagonism	Agonism	x	NCOA3 overexpression is required in order to maintain the telomerase activity.<br>NCOA3 is an inhibitor of senescence whose downregulation in aged individuals could be probably a tumor suppressor mechanism, preventing the clonal expansion of risky old cells from having damaged DNA.	PMID: 26469953
NR3C1	Yes	HC	✓	Agonism	Antagonism	x	Cyproterone acetate inhibiting AR, PGR & NR3C1 (approved for human use) extended lifespan in *C. elegans* .	PMID: 24134630
PDGFRB	Yes	HC	✓	Antagonism	Not suitable for antagonsim	x	PDGFR-β signaling in the cardiomyocyte may regulate angiogenesis in the heart in response to load-induced stress through several different mechanisms.<br>Imatinib mesylate inhibiting PDGFRB, ABL1, KIT, BCR reduced lifespan in *C. elegans* .	PMID: 20071776,<br>PMID: 3201088
PKM	No	HC	✓	Antagonism	Not suitable for antagonsim	x	PKM is involved in innate immune system during healthy aging.<br>In addtion, loss of PKM2 impairs angiogenic sprouting and loss of endothelial PKM2 alters mitochondrial metabolism.	PMID: 30301887
PLK1	Yes	HC	✓	Antagonism	Not suitable for antagonsim	x	PLK1 is involved in cell cycle regulation and DNA checkpoint during healthy aging;<br>PLK1 promotes autophagy.	PMID: 28102733,<br>PMID: 26597721
POLE	Yes	Novel	✓	Antagonism	Not suitable for antagonsim	x	Tumor suppressor gene	-
PPP5C	No	Novel	✓	Antagonism	Not suitable for antagonsim	x	PPP5C is invovled in DNA double-strand break repair during healthy aging.	Reactome

PTK2	Yes	HC	✓	Antagonism	Not suitable for antagonsim	x	PTK2 is involved in innate immune system during healthy aging.	Reactome
RAC1	No	HC	✓	Antagonism	Not suitable for antagonsim	x	RAC1 is involved in adaptive immune system and innate immune system during healthy aging.	-
RBCK1	No	Novel	✓	Antagonism	Not suitable for antagonsim	x	RBCK1 depletion increases p53 protein levels and p53 target genes, and<br>RBCK1 interacts with PTEN (tumor suppressor geen) and promotes PTEN degradation in K48-linked ubiquitination.<br>Dominant-negative versions of Drosophila melanogaster p53 in adult neurons extends lifespan.	PMID: 30874541,<br>PMID: 35174471
RHOA	No	HC	✓	Antagonism	Agonism	x	RHOA is invovled in protecting against the progression of cardiac aging.	https://escholarship.org/uc/item/14g0843j
RHOH	No	Novel	✓	Antagonism	Not suitable for antagonsim	x	Tumor suppressor gene	-
SMARCA4	No	HC	✓	Antagonism	Not suitable for antagonsim	x	Tumor suppressor gene	-
SMO	Yes	Novel	✓	Antagonism	Not suitable for antagonsim	x	SMO inhibition promotes aging.<br>SMO has shown to be critical for the hedgehog signal transduction on the cell membrane. Hedgehog signaling is dysregulated in old hepatocytes, and this accelerates aging.<br>Deleting SMO in young hepatocytes before partial hepatectomy prevented hedgehog pathway activation after partial hepatectomy and inhibited regeneration.<br>In addition, hedgehog inhibition promoted telomere shortening and mitochondrial dysfunction in hepatocytes.	PMID: 34984806
STK26	No	Novel	✓	Antagonism	Not suitable for antagonsim	x	Depletion of STK24 (or MST4) in mice promoted gastric tumorigenesis.	PMID: 32271880
TAOK1	No	Novel	✓	Antagonism	Not suitable for antagonsim	x	TAOK1 is invovled for cell cycle checkpoints during healthy aging	Reactome
TLR4	Yes	HC	✓	Antagonism*	Conditional	x	TLR4 is an important Pattern Recognition Receptor (PRR), which activates both innate and adaptive immune cells.<br>Its activation leads to inflammatory cytokine production which is responsible for activating the innate immune system.<br>Additional cytokine production (agonist) may not necessary for the healthy but reduced production (antagonist) may alter immune repsonse<br>The Toll-like receptor 4 (TLR4) signaling pathway is involved in many aspects of biological functions of AML cells, including the regulation of pro-inflammatory cytokine products, myeloid differentiation, and survival of AML cells.	PMID: 32508811,<br>PMID: 30336978
TNF	Yes	HC	✓	Antagonism	Conditional<br>(Antagonism for old)	x	TNF-α antagonism rescues the effect of ageing on stroke; TNF-α/IFN-γ synergy amplifes senescence-associated inflammation.<br>TNF-deficient mice develop normally but are more susceptible to some infectious agents.	PMID: 34076259,<br>PMID: 35645319
TOP2A	Yes	HC	✓	Antagonism	Not suitable for antagonsim	x	TOP2A is involved in cell cycle regulation during healthy aging.<br>Topoisomerases regulate the topological states of DNA and important for neuron proliferation.	PMID: 7980433
TRAF7	No	Novel	✓	Antagonism	Not suitable for antagonsim	x	Tumor suppressor gene	-
TRIM37	No	Novel	✓	Antagonism	Not suitable for antagonsim	x	TRIM37 is involved in adaptive immune system during healthy aging.	Reactome
TTK	No	Novel	✓	Antagonism	Agonism	x	Using bone-targeting recombinant adeno-associated virus 9 (rAAV9) to enhance Bmal1 or Ttk might have a therapeutic effect on senile osteoporosis and delays bone repair in aging mice.	https://doi.org/10.1016/j.omtn.2023.02.014
UBE2C	No	HC	✓	Antagonism	Not suitable for antagonsim	x	UBE2C is involved in cell cycle checkpoints during healthy aging	Reactome
UBE2L6	No	Novel	✓	Antagonism	Not suitable for antagonsim	x	UBE2L6 is invovled in adaptive immune system during healthy aging.	Reactome
UBE2M	No	Novel	✓	Antagonism	Not suitable for antagonsim	x	UBE2M is invovled in adaptive immune system during healthy aging.	Reactome
VEGFA	Yes	HC	✓	Antagonism	Not suitable for antagonsim	x	Counteracting age-related VEGF signaling insufficiency promotes healthy aging and extends lifespan.	PMID:34326210
ARL2	No	Novel	-	Antagonism	-	-	-	-
CTSB	No	HC	-	Antagonism	-	-	-	-
CTSV	No	Novel	-	Antagonism	-	-	-	-
HDAC10	Yes	Novel	-	Antagonism	-	-	-	-
HERC5	No	Novel	-	Antagonism*	-	-	-	-
ITGA9	No	Novel	-	Agonism	-	-	-	-
PIP5K1A	No	Novel	-	Antagonism	-	-	-	-
PIP5K1C	No	Novel	-	Antagonism*	-	-	-	-
PRIM1	Yes	Novel	-	Antagonism	-	-	-	-
PSMA2	Yes	Novel	-	Antagonism	-	-	-	-
PSMA3	Yes	Novel	-	Antagonism	-	-	-	-
PSMA4	Yes	Novel	-	Antagonism	-	-	-	-
PSMB1	Yes	Novel	-	Antagonism	-	-	-	-
PSMB6	Yes	Novel	-	Antagonism	-	-	-	-
PSMC1	Yes	Novel	-	Antagonism	-	-	-	-
PSMC4	Yes	Novel	-	Antagonism	-	-	-	-
PTPRF	No	Novel	-	Antagonism	-	-	-	-
RAB1B	No	Novel	-	Antagonism	-	-	-	-
TOP2B	Yes	Novel	-	Antagonism*	-	-	-	-
UBA2	No	Novel	-	Antagonism	-	-	-	-
UBE2T	No	Novel	-	Antagonism	-	-	-	-
USP11	No	Novel	-	Antagonism	-	-	-	-

[1] Common cancer targets from group 4 (Figure 5; Table S6)

[2] Any occurrence in the 90 overlapped biological processes enriched with 51 targets (gorup 1 and group 2) contributed to lifespan extension
[3] Generally, upregulation in expression leads to proposed antagonist for cancer; downregulation in expression leads to proposed agonist for cancer. * The therapeutic approach is proposed based on target's mechanism of action (see Table S6 for details)

Table S8. Gene Ontology terms commonly enriched by lifespan-extending (Group 1 and Group 2) and Group 4 targets

ID	Description	GeneRatio	pvalue	p.adjust	geneID
GO:0046777	protein autophosphorylation	19/101	5.32E-19	2.96E-16	LYN/SRC/ABL1/PTK2/KDR/AURKA/KIT/PDGFRB/AURKB/FGFR1/FGR/FGFR2/HCK/MAPKAPK5/TAOK1/FES/MAPKAPK3/STK26/TTK
GO:0007169	transmembrane receptor protein tyrosine kinase signaling pathway	15/101	1.52E-15	5.64E-13	LYN/SRC/PTK2/KDR/BLK/KIT/PDGFRB/FGFR1/FGR/FGFR2/HCK/MET/MST1R/FRK/FES
GO:0014068	positive regulation of phosphatidylinositol 3-kinase signaling	10/101	3.51E-11	7.79E-09	SRC/TNF/PTK2/KDR/VEGFA/FGF2/KIT/PDGFRB/FGFR1/FGR
GO:0043410	positive regulation of MAPK cascade	12/101	2.95E-10	5.45E-08	TNF/KDR/VEGFA/FGF2/HMGB1/KIT/PDGFRB/FGFR1/FGFR2/ADRA2C/TRAF7/KSR1
GO:0030335	positive regulation of cell migration	13/101	1.45E-09	1.61E-07	LYN/PTK2/IL1B/KDR/VEGFA/ITGAV/KIT/MMP2/PDGFRB/CXCR4/FGR/SMO/RHOA
GO:0016570	histone modification	9/101	2.55E-08	2.06E-06	AURKA/KDM1A/AURKB/NCOA1/PKM/RPS6KA4/KAT6A/BAZ1B/NCOA3
GO:0043536	positive regulation of blood vessel endothelial cell migration	6/101	5.85E-08	3.82E-06	KDR/VEGFA/FGF2/HMGB1/FGFR1/AKT3
GO:0018105	peptidyl-serine phosphorylation	10/101	9.05E-08	5.02E-06	SRC/AURKA/MAPKAPK5/PLK1/RPS6KA6/AKT3/RPS6KA2/RPS6KA4/MAPKAPK3/TTK
GO:0043065	positive regulation of apoptotic process	12/101	1.77E-07	8.55E-06	SRC/TNF/ABL1/HMGB1/MMP2/PDGFRB/TOP2A/RPS6KA2/RARB/DNM2/NCOA1/RBCK1
GO:0033674	positive regulation of kinase activity	7/101	1.87E-07	8.63E-06	KDR/KIT/PDGFRB/FGFR1/FGFR2/MET/MST1R
GO:0035556	intracellular signal transduction	14/101	2.23E-07	9.65E-06	LYN/SRC/BLK/KIT/PDGFRB/MAPKAPK5/RPS6KA6/AKT3/RPS6KA2/ITK/RAC1/RPS6KA4/TAOK1/MAPKAPK3
GO:0051897	positive regulation of protein kinase B signaling	8/101	3.00E-07	1.23E-05	SRC/TNF/PTK2/VEGFA/FGF2/FGFR1/MET/MST1R
GO:0043525	positive regulation of neuron apoptotic process	6/101	3.95E-07	1.56E-05	TNF/ABL1/NR3C1/RHOA/CDC34/UBE2M
GO:0048661	positive regulation of smooth muscle cell proliferation	6/101	7.95E-07	2.76E-05	TNF/TLR4/FGF2/MMP2/PDGFRB/FGFR2
GO:1904707	positive regulation of vascular associated smooth muscle cell proliferation	5/101	2.03E-06	6.45E-05	SRC/TNF/FGF2/MMP2/DNMT1
GO:0006954	inflammatory response	12/101	6.86E-06	1.76E-04	LYN/TNF/IL1B/TLR4/HMGB1/KIT/CXCR4/HCK/FASN/RAC1/RPS6KA4/ADGRE5
GO:0071222	cellular response to lipopolysaccharide	8/101	6.98E-06	1.76E-04	SRC/TNF/ABL1/IL1B/TLR4/HMGB1/RARA/RHOA
GO:0042127	regulation of cell population proliferation	8/101	1.51E-05	3.36E-04	TNF/ABL1/PTK2/KIT/CDK6/DNMT1/FES/KSR1
GO:0070372	regulation of ERK1 and ERK2 cascade	4/101	1.75E-05	3.73E-04	LYN/IL1B/FGFR2/TRAF7
GO:0010629	negative regulation of gene expression	9/101	2.11E-05	4.34E-04	TNF/KDR/VEGFA/FGF2/AURKA/RARA/SMO/DNMT1/ANAPC2
GO:0045785	positive regulation of cell adhesion	5/101	3.09E-05	5.56E-04	SRC/TNF/VEGFA/ITGAV/RHOA
GO:1901224	positive regulation of NIK/NF-kappaB signaling	5/101	4.19E-05	7.15E-04	TNF/IL1B/TLR4/RHOA/RBCK1
GO:0071300	cellular response to retinoic acid	5/101	4.84E-05	8.14E-04	LYN/TNF/HDAC2/RARA/FGFR2
GO:0032496	response to lipopolysaccharide	7/101	5.01E-05	8.22E-04	TNF/IL1B/TLR4/HDAC2/FGFR2/CSF2RB/MAPKAPK3
GO:0043687	post-translational protein modification	4/101	5.04E-05	8.22E-04	AURKB/RPS6KA4/BAZ1B/UBE2M
GO:0071560	cellular response to transforming growth factor beta stimulus	5/101	5.19E-05	8.35E-04	SRC/ABL1/HDAC2/FGFR2/NR3C1
GO:0051091	positive regulation of DNA-binding transcription factor activity	6/101	6.72E-05	1.07E-03	TNF/IL1B/KDM1A/KIT/SMARCA4/TRIM37
GO:0045429	positive regulation of nitric oxide biosynthetic process	4/101	9.48E-05	1.37E-03	TNF/IL1B/TLR4/DNM2
GO:1901796	regulation of signal transduction by p53 class mediator	4/101	9.48E-05	1.37E-03	AURKA/AURKB/MAPKAPK5/KAT6A
GO:0050679	positive regulation of epithelial cell proliferation	5/101	9.94E-05	1.41E-03	KDR/VEGFA/FGF2/FGFR2/SMO
GO:0042542	response to hydrogen peroxide	4/101	1.49E-04	1.82E-03	SRC/MMP2/PDGFRB/STK26
GO:0009410	response to xenobiotic stimulus	8/101	1.60E-04	1.93E-03	LYN/SRC/TNF/ABL1/HDAC2/MMP2/DNMT3A/RHOA
GO:0001525	angiogenesis	8/101	1.82E-04	2.12E-03	PTK2/KDR/VEGFA/FGF2/ITGAV/MMP2/PDGFRB/FGFR2
GO:0043491	protein kinase B signaling	4/101	2.24E-04	2.51E-03	TNF/IL1B/KDR/FGF2
GO:1902895	positive regulation of miRNA transcription	4/101	2.24E-04	2.51E-03	TNF/FGF2/NR3C1/SMARCA4
GO:0051092	positive regulation of NF-kappaB transcription factor activity	6/101	2.99E-04	3.10E-03	TNF/IL1B/TLR4/TRIM37/RBCK1/RPS6KA4
GO:0071230	cellular response to amino acid stimulus	4/101	3.68E-04	3.65E-03	TNF/MMP2/DNMT3A/DNMT1
GO:0009612	response to mechanical stimulus	4/101	4.47E-04	4.33E-03	SRC/TNF/MMP2/RHOA
GO:0045471	response to ethanol	5/101	4.49E-04	4.33E-03	TNF/RARA/FGFR2/DNMT3A/RHOA
GO:0001570	vasculogenesis	4/101	5.37E-04	5.01E-03	KDR/VEGFA/ITGAV/SMO
GO:0042531	positive regulation of tyrosine phosphorylation of STAT protein	4/101	5.37E-04	5.01E-03	LYN/TNF/HDAC2/KIT
GO:0030155	regulation of cell adhesion	4/101	5.69E-04	5.22E-03	ABL1/PTK2/CXCR4/FES
GO:0008283	cell population proliferation	7/101	6.98E-04	6.16E-03	SRC/FGF2/KIT/AURKB/KRAS/SMO/RARB
GO:0007094	mitotic spindle assembly checkpoint signaling	3/101	7.04E-04	6.16E-03	AURKB/PLK1/TTK
GO:0050673	epithelial cell proliferation	4/101	7.14E-04	6.19E-03	KDR/FGF2/KIT/SMO
GO:0051301	cell division	9/101	7.20E-04	6.19E-03	AURKA/AURKB/CDK6/NR3C1/PLK1/RHOA/CDC34/ANAPC2/UBE2C
GO:0048565	digestive tract development	3/101	8.53E-04	7.12E-03	KIT/FGFR2/SMO
GO:0042327	positive regulation of phosphorylation	3/101	1.11E-03	8.57E-03	LYN/KDR/VEGFA
GO:0009314	response to radiation	3/101	1.31E-03	9.95E-03	TNF/KIT/MMP2
GO:0014070	response to organic cyclic compound	5/101	1.35E-03	1.02E-02	LYN/TNF/KDM1A/PDGFRB/CXCR4
GO:0007568	aging	5/101	2.05E-03	1.36E-02	FGF2/MMP2/PDGFRB/AURKB/DNMT3A
GO:0014823	response to activity	3/101	2.46E-03	1.51E-02	TNF/MMP2/CXCR4
GO:0033628	regulation of cell adhesion mediated by integrin	2/101	2.54E-03	1.51E-02	LYN/PTK2
GO:0051239	regulation of multicellular organismal process	2/101	2.54E-03	1.51E-02	TNF/PTK2
GO:0001666	response to hypoxia	5/101	2.63E-03	1.54E-02	TNF/VEGFA/MMP2/CXCR4/RHOA
GO:0007173	epidermal growth factor receptor signaling pathway	3/101	2.78E-03	1.60E-02	SRC/ABL1/PTK2
GO:0042789	mRNA transcription by RNA polymerase II	3/101	2.78E-03	1.60E-02	RARA/NCOA1/HLTF
GO:0043124	negative regulation of I-kappaB kinase/NF-kappaB signaling	3/101	2.78E-03	1.60E-02	ABL1/RHOA/RHOH

GO:0030324	lung development	4/101	2.82E-03	1.61E-02	KDR/VEGFA/FGF2/FGFR2
GO:0060644	mammary gland epithelial cell differentiation	2/101	2.95E-03	1.64E-02	FGF2/SMO
GO:0010718	positive regulation of epithelial to mesenchymal transition	3/101	2.95E-03	1.64E-02	PTK2/IL1B/HDAC2
GO:0031648	protein destabilization	3/101	3.12E-03	1.71E-02	SRC/PLK1/MDM2
GO:0006351	DNA-templated transcription	7/101	3.15E-03	1.71E-02	RARA/NR3C1/DNMT1/RARB/PPP5C/KAT6A/KDM2A
GO:0071168	protein localization to chromatin	2/101	3.39E-03	1.77E-02	PLK1/MCM8
GO:0048146	positive regulation of fibroblast proliferation	3/101	3.49E-03	1.81E-02	ABL1/PDGFRB/CDK6
GO:0050727	regulation of inflammatory response	4/101	3.60E-03	1.86E-02	LYN/TNF/TLR4/HCK
GO:0090398	cellular senescence	3/101	3.89E-03	1.94E-02	ABL1/MAPKAPK5/KAT6A
GO:0000086	G2/M transition of mitotic cell cycle	3/101	4.10E-03	2.02E-02	AURKA/PLK1/DNM2
GO:0034097	response to cytokine	3/101	4.31E-03	2.08E-02	MMP2/RARA/MAPKAPK3
GO:0071456	cellular response to hypoxia	4/101	4.80E-03	2.21E-02	SRC/VEGFA/DNMT3A/MDM2
GO:0030889	negative regulation of B cell proliferation	2/101	4.88E-03	2.21E-02	LYN/BLK
GO:0036211	protein modification process	4/101	5.10E-03	2.30E-02	ABL1/CDC34/UBE2M/UBE2L6
GO:0033189	response to vitamin A	2/101	5.44E-03	2.38E-02	RARA/DNMT3A
GO:2000811	negative regulation of anoikis	2/101	5.44E-03	2.38E-02	SRC/PTK2
GO:0010468	regulation of gene expression	7/101	5.54E-03	2.39E-02	CDK6/DNMT3A/MDM2/SMO/DNMT1/SMARCA4/DNMT3B
GO:0070301	cellular response to hydrogen peroxide	3/101	5.99E-03	2.53E-02	SRC/ABL1/HDAC2
GO:0043154	negative regulation of cysteine-type endopeptidase activity involved in apo	3/101	6.54E-03	2.73E-02	SRC/TNF/VEGFA
GO:0002223	stimulatory C-type lectin receptor signaling pathway	2/101	6.63E-03	2.74E-02	LYN/SRC
GO:0000082	G1/S transition of mitotic cell cycle	3/101	6.82E-03	2.81E-02	CDK6/POLE/CDC34
GO:0001932	regulation of protein phosphorylation	3/101	7.71E-03	3.16E-02	LYN/TNF/PTK2
GO:0060045	positive regulation of cardiac muscle cell proliferation	2/101	7.92E-03	3.21E-02	FGF2/FGFR2
GO:0032091	negative regulation of protein binding	3/101	1.00E-02	3.85E-02	AURKA/KDM1A/AURKB
GO:0048714	positive regulation of oligodendrocyte differentiation	2/101	1.01E-02	3.85E-02	HDAC2/CXCR4
GO:0008285	negative regulation of cell population proliferation	7/101	1.09E-02	4.11E-02	LYN/TNF/IL1B/FGF2/RARA/CDK6/RPS6KA2
GO:0032148	activation of protein kinase B activity	2/101	1.24E-02	4.43E-02	SRC/ADRA2C
GO:0035924	cellular response to vascular endothelial growth factor stimulus	2/101	1.24E-02	4.43E-02	KDR/VEGFA
GO:0043200	response to amino acid	2/101	1.24E-02	4.43E-02	LYN/RHOA
GO:0071480	cellular response to gamma radiation	2/101	1.24E-02	4.43E-02	KDM1A/MDM2
GO:0010592	positive regulation of lamellipodium assembly	2/101	1.33E-02	4.70E-02	RAC1/DNM2
GO:0097421	liver regeneration	2/101	1.41E-02	4.88E-02	TNF/AURKA

Table S9. Description for scores and filters on PandaOmics.

Category	Score/ Filter	Description
Omics AI scores	Network Neighbors	This score utilizes several graph-based methods applied to the protein-protein interaction network enriched with differentially expressed/methylated genes. The score explores direct network neighbors interacting with a given gene. A target will be scored higher if there are more network neighbors with significant differences in expression or methylation levels.
	Casual Inference	This score is based on the causal inference of transcription factors. It estimates the number of genes associated with the disease progression/treatment, controlled by a similar set of transcription factors to a given gene. It uses a manually curated regulatory network and known drug targets to predict potential disease-modifying transcription factors.
	Pathways	The score combines several approaches to pathway analysis. First, iPANDA algorithm is used to examine the involvement of a given gene in pathway activation patterns based on a collection of gene expression datasets of interest (activation/inhibition of each pathway is examined separately). Next, all the pathways from the library are merged into a single network, which is examined from the perspective of signal propagation by a number of methods. The final score indicates how a given gene affects individual pathways' activation/inhibition, and whether it possesses the ability to affect multiple pathways at once.
	Interactome Community	This score utilizes several graph-based methods applied to the protein-protein interaction network enriched with active drug targets, GWAS hits and differentially expressed/methylated genes.
	Relevance	This score is based on open data from the OpenTargets resource, in particular, Drugs score. Drugs score aggregates general information about all drugs and their stages of clinical trials that are associated with a specific gene (protein, molecular target) for a disease of interest. The more drugs that have a specific gene as a molecular target, especially in the late phases of clinical trials, the higher the Drugs score for that gene.
	Expression	This score takes into account differential gene expression, protein abundance or methylation level defined by a collection of datasets of interest. Machine learning-based models are used to normalize available omics data across multiple samples from various datasets.

	Heterogeneous Graph Walk	This score is a guided random walk-based approach that is applied to a heterogeneous graph (i.e., a graph containing different types of nodes). The model learns node representations and then finds the gene nodes similar to the reference disease node. First, the "walks" are sampled with a predefined meta-path, i.e., the fixed sequence of node types in a walk, e.g. 'gene'-'disease'-'gene.' The node degree controls the probability of transition between the nodes while sampling. Following that, the AI model learns the representation of each node based on the resulting corpus of walks. The similarity between the specific disease and all available genes produces a ranked list of genes. The top genes from this list are predicted to be promising target hypotheses.
	Matrix Factorization	This score is based on a collaborative filtering algorithm, which is widely used in recommender systems. First, well-known gene-disease associations from the PandaOmics database are converted to a sparse binary matrix.This matrix is then decomposed into two low-rank matrices that consist of latent factors for genes and diseases. The algorithm uses graph regularization based on a fast kNN search to account for the intraclass similarity between the nodes of a similar type. Recomputing the original interaction matrix from latent factors provides the scores for unobserved interactions; thus, gene ranking is obtained.
Text-based AI scores	Evidence	This score is calculated as the weighted average of Trend and Attention score. Higher values indicate both attention growth and a high volume of research.
	Attention Score	This score measures the overall attention to the target at all times. PandaOmics calculates the total number of mentions of a gene in various texts across all time periods. Both disease-agnostic and disease-specific mentions are counted. Text corpus used for analysis includes scientific publications, grants, patents and clinical trials.
Financial Scores	Grant Funding	Total grant funding to investigate the given gene at all times. Please note, that the distribution of total funding is skewed with mean value approx. $8.9 million, but the median value is only $1.7 million. Also 5% of entries have funding above $30 million.
KOL Scores	Credible Attention Index	This score represents the total number of publications devoted to the target gene in journals with an impact factor more than 10. This corresponds to the top 3% of all scientific journals.
	Impact factor	This score measures the average impact factor of the journals where particular gene-disease association is published. The Impact Factor of each journal is weighted to the number of publications mentioning gene-disease association published in this journal.

Druggability Filter	Small Molecules	Indicates accessibility of a protein by small molecules<br><br>Red - A protein does not belong to a druggable class and does not have small molecules associated<br>Yellow - A protein belongs to a druggable class and does not have small molecules associated<br>Green - A protein is associated with small molecules according to public sources and the PandaOmics manually curated database
	Antibodies	Indicates the ability to use antibodies to hit the target<br><br>Red - It is not membrane secreted protein (Protein atlas database)<br>Yellow - A membrane secreted protein (Protein atlas database)<br>Green - A membrane secreted protein (Protein atlas database) with associated antibodies in TCRD database
	Safety	Potential safety of a target and potential adverse effect<br><br>Red - A protein is an essential gene and did not pass any clinical trials (Clinical trials and TTD)<br>Yellow - Default status<br>Green - A protein is a conditional essential gene or is not essential and has clinical trials going on or passed (Clinical trials and TTD)
	Novelty	Indicates the overall scientific community interest to the target, based on the volume of related publications proposed by the proprietary AI engine<br><br>Red - More than 168 publications on pharmacognitive<br>Yellow - Between 50 and 168 publications on pharmacognitive<br>Green - Less than 49 publications on pharmacognitive
Target Familes	Enzyme	Druggable
	Epigenetic enzyme	Druggable
	Epigenetic nonenzyme	Not druggable
	Generic protein	Not druggable
	Ion channel	Druggable
	Kinase	Druggable
	Nuclear receptor	Druggable
	Receptor	Druggable
	Secretory protein	Druggable

	Transcription coregulator	Not druggable
	Transcription factor	Not druggable
	Transportor	Druggable
